# Bioactive small compounds effectively inhibit ChREBP overexpression to treat NAFLD and T2DM: A computational drug development approach

**DOI:** 10.1016/j.heliyon.2025.e42477

**Published:** 2025-02-10

**Authors:** Hiron Saraj Devnath, Maisha Maliha Medha, Md Naharul Islam, Partha Biswas, Debasree Sen Oisay, Arafat Hossain, Rubaet Sharmin Ema, Md Mohaimenul Islam Tareq, Mimi Golder, Md Nazmul Hasan, Biswajit Biswas, Samir Kumar Sadhu

**Affiliations:** a Pharmacy Discipline, Khulna University, Khulna, 9208, Bangladesh; b Laboratory of Pharmaceutical Biotechnology and Bioinformatics, Department of Genetic Engineering and Biotechnology, Jashore University of Science and Technology, Jashore, 7408, Bangladesh; c Biochemistry and Molecular Biology Department, Life Science Faculty, Bangabandhu Sheikh Mujibur Rahman Science and Technology University, Gopalgonj, 8100, Bangladesh; d Department of Pharmacy, Faculty of Biological Science and Technology, Jashore University of Science and Technology, Jashore, 7408, Bangladesh

**Keywords:** T2DM, NAFLD, ChREBP-targeted therapy, Insulin resistance, Molecular dynamic simulation

## Abstract

A glucose-dependent carbohydrate-signaling gene regulator named Carbohydrate response element binding protein (ChREBP), has recently been discovered as a major metabolic regulator of enzymes involved in the progression of non-alcoholic fatty liver disease (NAFLD) and type-II diabetes mellitus (T2DM). As a result, this research is aimed to identify natural small molecules as drug candidates that target the ChREBP in order to counter aggressive NAFLD and T2DM. A comprehensive *in silico* drug design strategy was implemented to find possible inhibitors of the targeted protein. A site-specific molecular docking approach was used to screen 20 FDA approved anti-diabetic drugs and 494 phytochemicals from the natural sources against the ChREBP, and the top ten compounds were selected for further studies based on their binding affinities. The ADME and toxicity profiles of the selected ten drug compounds demonstrated their efficacy and safety. The result of the MD simulations of the protein–ligand complex structures indicated their stability and potential activity. A comprehensive data screening process following docking, ADMET properties, and MD simulation approaches, five compounds (dieckol, isocorilagin, stachyurin, stachysetin and thonningianin A) with favorable values against the targeted ChREBP were demonstrated which indicates their strong potential as promising and effective drug candidates for the treatment of NAFLD and T2DM.

## Graphical abstract

**Figure:**
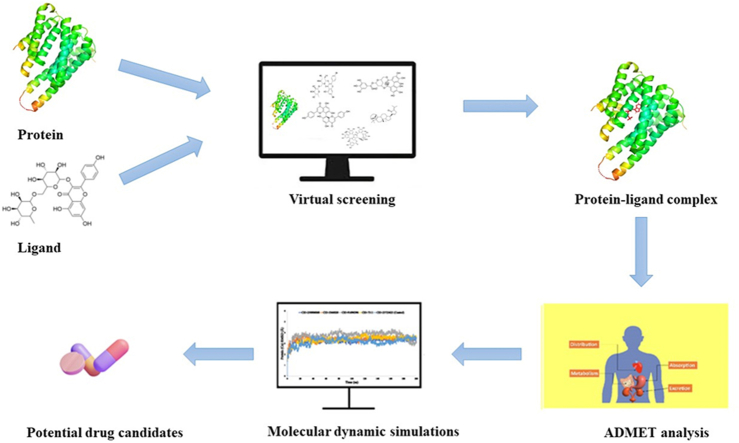

## 1 Introduction

Excessive sugar intake and defective glucose sensing in liver cells contribute to metabolic diseases like type 2 diabetes (T2DM) and nonalcoholic fatty liver disease (NAFLD). Carbohydrate response element binding protein (ChREBP), a transcription factor, senses intracellular carbohydrates and activates genes involved in *de novo* lipogenesis (DNL), increasing fatty acid synthesis and triglyceride storage in liver cells, leading to hepatic steatosis. This lipid accumulation is often associated with T2DM, with 75 % of T2DM patients having NAFLD and 80 % higher liver fat content compared to non-diabetics, indicating a high risk of fatty liver complications and NASH [1]. As of 2020, there are no FDA-approved pharmacological therapies for NAFLD, highlighting a significant unmet need. Research focuses on targeting the insulin-sensitizing pathway to develop treatments. Key molecular targets in NAFLD development include ChREBP, free fatty acids (FFAs), glucagon-like peptide 1 (GLP-1), interleukins (IL), nuclear factor κB (NF-κB), peroxisome proliferator-activated receptor γ (PPAR-γ), reactive oxygen species (ROS), sterol regulatory element binding protein (SREBP-1c), and tumor necrosis factor (TNF-α) [2]. ChREBP plays a crucial role in converting carbohydrates into lipids and storing energy as triglycerides in liver cells. Targeting ChREBP and its downstream effects offers potential for developing treatments for NAFLD and T2DM [[3], [4], [5], [6]].

As the NAFLD is closely linked to insulin resistance, while lipid buildup in insulin-sensitive tissues leads to lipotoxicity and insulin resistance, lipid synthesis via DNL remains high during insulin resistance, potentially creating a cycle of lipid-induced insulin resistance. Interestingly, hepatic triglyceride deposition may buffer more harmful lipid species, potentially preventing insulin resistance and other systemic issues [6]. DNL is crucial in the development of fatty liver disease, especially NAFLD. In NAFLD, DNL is activated, leading to excessive fat production and lipid accumulation in the liver. DNL synthesizes fatty acids and triglycerides from non-lipid sources, primarily in response to high carbohydrate levels or insulin signaling. This process is regulated by transcriptional and allosteric mechanisms, involving key transcription factors like SREBP1c and ChREBP Glucose influx and insulin signaling activate SREBP1c and ChREBP. Normally, SREBP1c is bound to SCAP and INSIG1 in the endoplasmic reticulum, preventing its nuclear translocation. Upon insulin signaling, INSIG1 dissociates, allowing SREBP1c to be cleaved by proteases in the Golgi apparatus, releasing its active form to enter the nucleus. Similarly, ChREBP, anchored to the 14-3-3 protein, becomes phosphorylated and enters the nucleus. In the nucleus, both SREBP1c and ChREBP bind to promoter regions of lipogenic genes like FAS, SCD1, and ACC, initiating their transcription and promoting lipogenesis. Inhibiting SREBP1c and ChREBP reduces the expression of these lipogenic genes, thereby decreasing lipogenesis. AMPK (AMP-activated protein kinase), a critical regulator of metabolic processes in diseases like NAFLD, suppresses DNL by inhibiting both SREBP1c and ChREBP, thus reducing lipogenesis and helping to counteract lipid accumulation in the liver [3].

DM is the third leading cause of morbidity and mortality globally, with 415 million affected in 2015, projected to rise to 140 million in Southeast Asia by 2040 [7]. Insulin resistance is implied to be the key pathological principle of T2DM, owing to the postprandial changes in hepatic glucose metabolism such as increased post-absorptive glucose production and diminished glucose uptake [8,9]. The liver control carbohydrate metabolism by regulation of glucose breakdown and triglyceride synthesis through a system of enzymes and kinases in order to provide substrates such as acetyl CoA for fatty acids [10,11]. Following an excess of carbohydrate ingestion, these liver enzymes get activated either by dephosphorylation allosteric mechanisms in short term or gene transcription as a long-term response [[12], [13], [14]]. The enzymes catalyzing the biosynthesis of fatty acids, namely acetyl CoA carboxylase (ACC), fatty acid synthase (FAS), and livertype pyruvate kinase (L-PK) have increased mRNA levels in diets favoring lipogenesis [15,16]. Recently, a glucose-dependent carbohydrate-signaling gene regulator called ChREBP well known as MLXIPL or MondoB, has emerged as a central metabolic regulator of enzymes involved in glycolysis and lipogenesis [17,18]. It is a basic helix-loop-helix (b/HLH) leucine-zipper (LZ) transcription factor made up of 864 amino acids and with a molecular weight of 94600 Da [19]. Liver is one of the main expression site of ChREBP as lipogenesis is highly active in the liver [4]. In response to glucose, hepatocytes containing ChREBP moves to and from the cytoplasm and nucleus [20]. ChREBP expression is also seen in significantly in small intestine, pancreatic islets, skeletal muscle and less so in the kidney and the brain [18,21]. The majority of ChREBP target genes are involved in metabolism, insulin signaling, and tumorigenesis as identified through Chromatin immunoprecipitation sequencing (ChIP-seq) analysis of genome-wide ChREBP binding [22]. The consensus sequence CAYGYGnnnnnCRCRTG has been identified as the most common site for ChREBP binding by precise motif analysis [23].

Detailed process of ChREBP regulation is still unknown but its transactivation partially takes place by phosphorylation and dephosphorylation [24], even though this theory has been recently challenged. This ChREBP stimulation happens in two levels: (i) glucose metabolites-induced nucleur translocation from cytosol and (ii) co-factor coordinated activation of target DNA binding transcription [25,26]. Under fasting conditions, inactivation of ChREBP transactivity takes place through phosphorylation by protein kinase A and AMP-activated protein kinase and ChREBP is translocated in the cytoplasm [27]. During carbohydrate feeding, as increased levels of glucose intermediate metabolites possibly bind to specific ChREBP motifs, a probable allosteric conformational change in ChREBP takes place and it initializes target gene transcription [28]. The transcription of target genes is activated by ChREBP in a glucose-dependent manner by forming heterodimers with its obligatory interaction partner Max-like factor X (Mlx), translocating into nucleus and binding to carbohydrate-response elements (ChoRE) sequences in the promoters of the glycolytic (L-pk), lipogenic (Acc, Fasn, Scd1, and Elovl6) and other (Fgf21 and Txnip) genes [[28], [29], [30]]. The relatively abundant and stable Mlx enhances DNA binding of ChREBP [31] and ChoRE is required for glucose-dependent transcription of genes encoding glycolytic and lipogenic enzymes in hepatocytes and adipose cells [32] ChREBP recognizes the livertype pyruvate kinase (L-PK) promoter ChoRE by the presence of two E-box–like sequences (C[C/A]CG[G/T]G) separated by 5 nucleotides [33].

Insulin sensitivity readily improves upon liver fat content reduction as excess liver fat causes insulin resistance [34,35]. It has been found that the effects of the metabolic syndrome such as obesity, fatty liver, insulin resistance, glucose intolerance can be decreased by complete ChREBP inhibition can as seen in ob/ob mice [27]. Defects in insulin secretion is one of the main characteristics of T2DM, which was seen to improve in ChREBP- silenced MIN6 cells and deteriorate in ChREBP-overexpressed INS1 cells [29]. ChREBP also causes reduced fatty acid oxidation under chronic hyperglycemia by repressing lipid-activated transcription factor PPAR-α which possibly results in lipid accumulation seen in b cells and their consequent failure [29,36]. Furthermore, a dramatic increase in hepatic ChREBP expression in Type 2 Diabetes patients demonstrates how ChREBP has a role in development of IR and T2DM [28]. Hence, it is imperative to understand the ChREBP-mediated transcriptional control of glycolytic and lipogenic gene expression in order to develop novel strategies for treatment and prevention of hepatic insulin resistance and T2DM [37].

Currently, an increasing number of studies have focused on herbal extracts or natural products, and many of these studies have discovered herbal products with potent effects against NAFLD and T2DM. Herbal medicines have gathered growing attention as potential therapeutic agents to prevent and treat non-alcoholic fatty liver fisease (NAFLD) and type 2 diabetes mellitus (T2DM) due to their high efficacy and low risk of side effects [[38], [39], [40], [41]]. Medicinal plants are widely recommended by the WHO for their availability, affordability, and social acceptance, particularly in underdeveloped countries where conventional treatments may be less accessible. Modern research now focuses on isolating specific compounds from plants to better understand their mechanisms and therapeutic efficacy, moving away from crude extracts toward targeted, compound-based approaches.

Although there have been many treatment strategies for T2DM and NAFLD, very few candidates are available in clinical practice. In this regard, ChREBP can be an effective target for preventing or treating this metabolic disease. In this research, we performed an *in-silico* screening to find potential ChREBP inhibitors from the pool of FDA approved antidiabetic drugs and established antidiabetic compounds obtained through literature review. The best scoring compounds were evaluated for their pharmacodynamic and pharmacokinetic properties along with toxicity level analysis for opportunities in the treatment of NAFLD and T2DM.

## 2 Literature search and selection of criteria

To conduct this research, data were collected by searching literatures from Pubmed, Google scholar. The following keywords are used to search the literatures: antidiabetic, compounds, plants, *in vitro*, *in vivo* etc. Compounds selected (Supplementary Tables 1 and 2) in this study have shown either *in silico*, *in vitro* or *in vivo*, antidiabetic property.

## 3 Materials and methods

### 3.1 Protein preparation

Protein's PDB file was downloaded from Protein Data Bank. Protein was then purified, and polar hydrogens were added by using Discovery Studio Visualizer 64-bit version 20.1.0.19295 (Systèmes 2020). Afterwards, SwissPDB viewer software version 4.1.0 was used for energy minimization of the protein. The protein model was validated using procheck server (https://saves.mbi.ucla.edu/).

### 3.2 Active sites determination

**Fig. 1:**
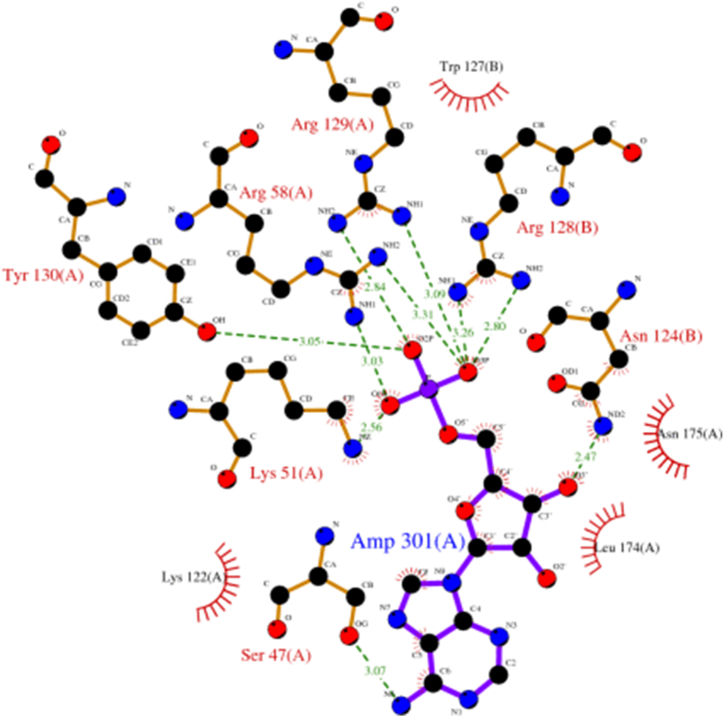
The 2D interactions among the protein active site residues and the involving ligand AMP.

The PDB conformation 5F74 corresponds to a well-characterized structure of the target protein, which has been experimentally resolved and validated through X-ray crystallography. AMP inhibits the nuclear translocation of ChREBP by allosterically enhancing its interaction with 14-3-3 proteins, rather than through the activation of AMPK. As a result, AMP, in combination with ketone bodies, limits lipogenesis by preventing the nuclear localization of ChREBP, thereby retaining it in the cytoplasm during periods of ketosis [42]. Additionally, AMP's binding region is well-conserved across related species, making it a suitable target for docking studies aimed at identifying potential inhibitors. Active site residues were determined by using Ligplot plus software version 2.2.4. The 2D interactions among the protein active site residues and the involving ligand AMP was depicted in Fig. 1.

### 3.3 Ligand preparation

Ligands were downloaded in SDF format with the help of PubChem. Most of the ligand's structure was 3D. A few compounds were drawn using Chemdraw professional 15.0.0. The 2D structures of some compounds were converted into 3D using Chemdraw professional 15.0.0. Energy minimization of the ligands was done using Pyrx Autodock Vina 4.2.

### 3.4 Molecular docking and visualization

PyRx was used to perform the molecular docking studies between the compounds and the protein. Site specific molecular docking was performed by selecting the following Ser47(A), Arg58(A), Arg129(A), Tyr130(A), Lys51(A), Lys122(A), Leu174(A), Asn175(A), Trp127(B), Arg128(B) and Asn124(B) amino acids. The grid box centre value was x = 15.33, y = 40.96 and z = 8.57. The best docking poses were shown in Supplementary Table 3.

### 3.5 Selection of the ligands for further process

**Fig. 2:**
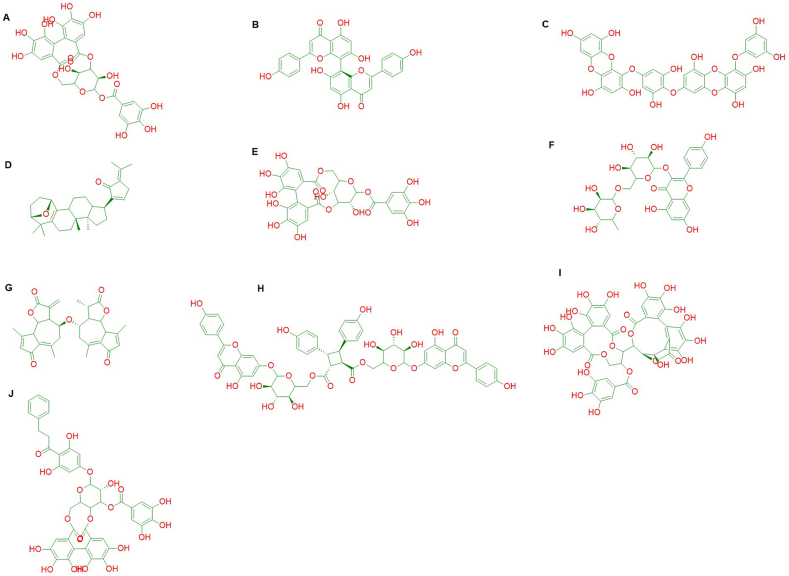
Chemical structures of the selected best 10 candidates: A = Corilagin, B = Cupressuflavone, C = Dieckol, D = Gypensapogenin A, E = Isocorilagin, F = Kaempferol-3-rutinoside, G = Lactucain A, H = Stachysetin, I = Stachyurin and J = Thonningianin A.

The binding affinity of the native ligand of ChREBP was found −7.4 kcal/mol. However, corilagin, stachyurin, dieckol, isocorilagin, thonningianin, lactucain, gypensapogenin, stachysetin, kaempferol-3-rutinoside and cupressuflavone showed the better binding affinities −10.2 kcal/mol, −10.4 kcal/mol, −10.8 kcal/mol, −10.6 kcal/mol, −11.3 kcal/mol, −10.7 kcal/mol, −10.2 kcal/mol, −10.4 kcal/mol, −10.1 kcal/mol, and −9.9 kcal/mol respectively compared to the native ligand and FDA approved anti-diabetic drugs represented by Supplementary Table 1. Among all the compounds, top ten compounds were selected for further *in silico* analysis depending on the binding affinities. ChemDraw Professional 15.0 is used to draw the structure of the compounds (Fig. 2A–J) where letters were assigned to indicate the specific compound as followed A = Corilagin, B = Cupressuflavone, C = Dieckol, D = Gypensapogenin A, E = Isocorilagin, F = Kaempferol-3-rutinoside, G = Lactucain A, H = Stachysetin, I = Stachyurin and J = Thonningianin A.

### 3.6 ADME/T, drug-likeliness, and medicinal chemistry evaluation

Pharmacokinetic properties were evaluated using SwissADME web tool (http://www.swissadme.ch/), a server providing robust predictive models for physicochemical properties, pharmacokinetics, drug-likeness as well as medicinal chemistry friendliness [43]. It was used to determine the bioavailability score of the ligands, hydrogen bonds acceptor and donor, topological polar surface area, molar refractivity, Lipinski's rule of five [44] and Ghose violation. Excretion of the ligands were predicted using the pkCSM online server (https://biosig.lab.uq.edu.au/pkcsm/prediction). Ligands toxicity was examined through the online servers preADMET and pkCSM, that predicts ames test, max tolerated dose, hERG I inhibitor, oral rat acute toxicity, oral rat chronic toxicity, hepatotoxicity, skin sensitization and many other related properties.

### 3.7 Reevaluation of docking score and binding energy calculation

For reevaluating the docking score, we have used the XP (extra precision) and molecular docking by Maestro (Schrödinger Release 2021-2: Maestro, Schrödinger, LLC, New York, NY, 2020-3). Firstly, we have prepared the protein by the protein preparation wizard. The following criteria's such as the assign bond orders, use CCD database, add hydrogen, create zero-order bonds to metals, create disulfide bonds, filled missing side chains and loops by using prime, fixed cap termini, and deleted waters beyond 5 Å from heat groups, generating heats states of pH 7.0±2.0 using Epik were performed for preparation of the selected protein. Then we prepared the selected ligands and generated a receptor grid of the protein for site specific docking to the targeted site of the natural ligand. After completing all the criteria, we have performed the docking for reevaluating the docking score. The molecular mechanics generalized born and surface area solvation (MMGBSA) technique in Maestro- Schrödinger (https://www.schrodinger.com/products/maestro) was used to analyze the relative binding affinity of the control and candidate ligands against the selected protein receptor.

### 3.8 Protein-ligand interaction analysis

Learning about the potential interactions between protein-ligand complexes is a key element of the drug development process, as it aids in the identification of hits to leads as prospective drug candidates. Interaction analysis also aids in determining the location of tiny molecules within proteins and their activity on biological networks. The accurate discovery of protein-ligand interactions is critical in medication development and disease therapy. As a result, the interaction between the ten chemicals chosen and the desired 5F74 protein was investigated using the BIOVIA Discovery Studio Visualizer tools. The complex structure analysis revealed many bonding interactions such as hydrogen bonds (Conventional H-B, Carbon H-B, and Pi-Donor H-B), electrostatic (Pi Anion), and hydrophobic (Alkyl, Pi-Alkyl, Pi-Pi T-shaped, and Pi-Sigma) between the protein and ligand.

### 3.9 Molecular dynamics simulation (MDS) analysis

The docked complexes were subjected to perform the MDS using YASARA [45,46] package with the aid of the AMBER14 force field [47]. The cubic simulation cells were generated using the TIP3P solvation model with a periodic boundary condition [48]. The complexes underwent an initial cleaning process, followed by optimization, and the hydrogen bond networks were aligned. The simulation cells were expanded by 20 Å in all directions from the complexes in each case. pH 7.4, 310K, and 0.9 % NaCl were established as the physiological conditions of the simulation cells. The first energy minimizations were performed using steepest gradient methods using the simulated annealing approach (5000 cycles) [49,50]. The long-range electrostatic interactions were computed using the Particle Mesh Ewald method with a cutoff radius of 8.0 Å [51,52]. The simulation systems were set with a time step of 2.0fs. The simulation trajectories were saved at regular intervals of 100 ps. The simulations were performed for 100 ns using the constant pressure and Berendsen thermostat. Trajectories were analyzed to calculate the root mean square deviations (RMSD), root mean square fluctuations (RMSF), radius of gyrations (Rg), solvent accessible surface area (SASA), and hydrogen bond [[53], [54], [55]].

## 4 Results

### 4.1 Protein structure validation

**Fig. 3:**
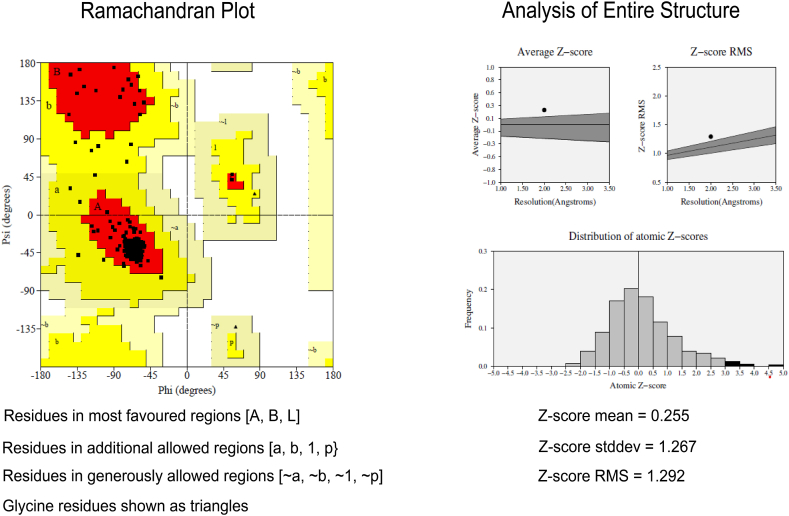
Ramachandran's Map of the modeled protein & distribution of atomic Z scores, average Z score and Z score RMS. A, B, L = Residues in most favored regions, a. b. 1, p = Residues in additional allowed regions, ∼a, ∼b, ∼1, ∼p = Residues in generously allowed regions.

The Ramachandran plot (Fig. 3) reveals the phi-psi torsion angles for all residues in the structure. The main chain parameters plotted are Ramachandran plot quality, peptide bond planarity, main chain hydrogen bond energy, Alpha carbon tetrahedral distortion, Bad nonbonded interactions and over-all G factor. In the Ramachandran plot analysis, the residues were classified based on its regions in the quadrangle. In the graph, the red regions denote the most allowed regions and the yellow regions indicate allowed regions. Glycine is indicated by triangles and other residues are indicated by squares. The result demonstrated that the modeled structure for ChREBP has 94 % residue in the allowed region. The model found was the best one having maximum core region and less disallowed region with minimum energy. The arrangement of the main chain bond lengths as well as bond angles were found to be within the limits for these proteins. Such particulars assigned by Ramachandran plot indicates a good quality of the protein model. The result of PROCHECK analysis was shown in Table 1.

**Table 1:** Ramachandran plot calculation and comparative analysis of the protein model computed with the PROCHECK server.

Server	Protein model	ChREBP
		5f74
PROCHECK	Residues in most favored regions	94.0 %
	Residues in additional allowed regions	6.0 %
	Residues in generously allowed regions	0.0 %
	Residues in disallowed regions	0.0 %

The average Z score, Z score RMS and distribution of atomic Z scores is represented in Fig. 3. The RMS Z-score of the protein under consideration as found in PROVE of SAVES server was 1.292, indicating good model quality. The analysis revealed Z-scores standard deviation was almost equal to 1, which suggests high model quality. The predicted structure conformed well to the stereochemistry, indicating reasonably good quality.

### 4.2 ADME/T, drug-likeliness, and medicinal chemistry evaluation of the ten top candidates

Physicochemical property: The SwissADME server was used in this section to predict the molecular and physicochemical characteristics including molecular formula, molecular weight, number of heavy atoms, number of aromatic heavy atoms, fraction csp3, number of rotatable bonds, number of H-bond acceptors, number of H-bond donors, molar refractivity and TPSA of the 10 candidates (Table 2).

**Table 2:** Predicted physicochemical properties of 10 best candidates.

Compound lists	A	B	C	D	E	F	G	H	I	J
**Molecular Formula**	C_27_H_22_ O_18_	C_30_H_18_ O_10_	C_36_H_22_ O_18_	C_30_H_42_ O_2_	C_27_H_22_ O_18_	C_27_H_30_ O_15_	C_30_H_32_ O_7_	C_60_H_52_ O_24_	C_41_H_28_ O_26_	C_42_H_34_ O_21_
**Molecular Weight (g/mol)**	634.45	538.46	742.55	434.65	634.45	594.52	504.57	1157.04	936.65	874.71
**No. of Heavy Atoms**	45	40	54	32	45	42	37	84	67	63
**No. of Aromatic Heavy Atoms**	18	32	36	0	18	16	0	44	30	30
**Fraction Csp3**	0.22	0.00	0.00	0.77	0.22	0.44	0.53	0.27	0.15	0.19
**No. of Rotatable Bonds**	3	3	6	1	3	6	2	16	4	9
**No. of H-bond Acceptors**	18	10	18	2	18	15	7	24	26	21
**No. of H-bond Donors**	11	6	11	0	11	9	0	12	16	12
**Molar Refractivity**	141.85	146.97	181.42	132.72	141.85	139.36	135.33	290.10	212.50	210.24
**TPSA (Å**^**2**^**)**	310.66	181.80	287.14	26.30	310.66	249.20	95.97	392.70	455.18	357.19

#### 4.2.1 Bioavailability radar

**Fig. 4:**
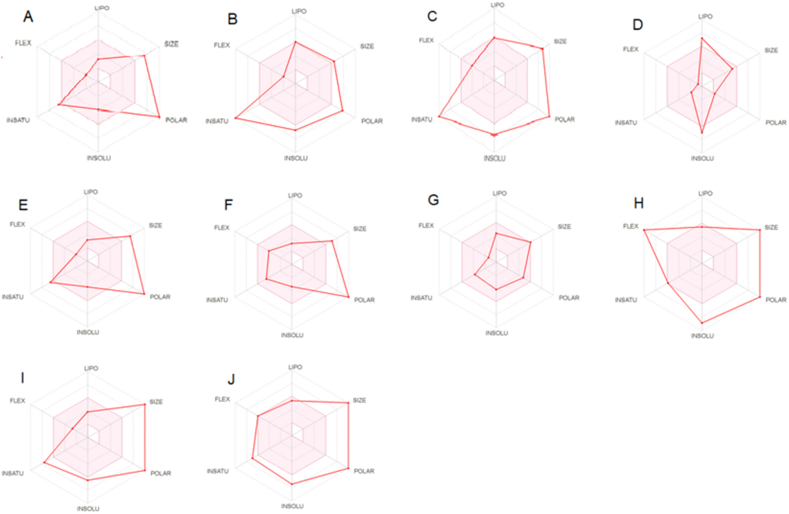
Bioavailability radar of the selected compounds: A = Corilagin, B = Cupressuflavone, C = Dieckol, D = Gypensapogenin A, E = Isocorilagin, F = Kaempferol-3-rutinoside, G = Lactucain A, H = Stachysetin, I = Stachyurin and J = Thonningianin A.

The compounds D and G both showed a bioavailability score of 55 %, whereas other compounds had the score of 17 %. Moreover, the bioavailability score and radar shape of 10 candidates represented in Table 3 & Fig. 4(A–J).

**Table 3:** Predicted bioavailability score of 10 best candidates.

Compound Lists	Bioavailability Score
A	0.17
B	0.17
C	0.17
D	0.55
E	0.17
F	0.17
G	0.55
H	0.17
I	0.17
J	0.17

#### 4.2.2 Lipophilicity

Among the 10 compounds, the compounds B, C and G had Consensus LogP values within this range, with their respective values being 3.5, 3.39 and 3.19 (Table 4).

**Table 4:** Lipophilic properties of the selected compounds.

Compound lists	Log *P*_o/w_ (iLOGP)	Log *P*_o/w_ (XLOGP3)	Log *P*_o/w_ (WLOGP)	Log *P*_o/w_ (MLOGP)	Log *P*_o/w_ (SILICOS-IT)	Consensus Log *P*_o/w_
A	1.24	0.07	−0.3	−2.42	−2.15	−0.71
B	2.44	5.04	5.13	0.25	4.61	3.5
C	2.66	4.87	7.62	0.04	1.75	3.39
D	4.68	7.1	7.35	5.7	6.78	6.32
E	1.24	0.07	−0.3	−2.42	−2.15	−0.71
F	2.79	0.02	−1.39	−3.43	−1.64	−0.73
G	3.25	2.17	3.75	2.75	4.05	3.19
H	2.33	4.04	3.44	−2.54	2.41	1.94
I	1.06	1.24	1.01	−3.23	−1.78	−0.34
J	2.74	3.83	3.02	−1.51	0.81	1.78

#### 4.2.3 Water solubility

The water solubility of all ten compounds ranges from insoluble to soluble. Compounds F and G exhibit moderate to good solubility, while compounds A, B, C, D, E, H, I, and J show poor solubility (Table 5). A more negative solubility value corresponds to lower solubility.

**Table 5:** Predicted water solubility parameters of 10 best candidates.

Compound Lists	Log *P*_o/w_ (iLOGP)	Log *P*_o/w_ (XLOGP3)	Log *P*_o/w_ (WLOGP)	Log *P*_o/w_ (MLOGP)	Log *P*_o/w_ (SILICOS-IT)	Consensus Log *P*_o/w_
A	−3.92	Soluble	−6.15	Poorly Soluble	−0.51	Soluble
B	−6.75	Poorly Soluble	−8.6	Poorly Soluble	−8.7	Poorly Soluble
C	−7.61	Poorly Soluble	−10.63	Insoluble	−6.32	Poorly Soluble
D	−6.94	Poorly Soluble	−7.47	Poorly Soluble	−6.66	Poorly Soluble
E	−3.92	Soluble	−6.15	Poorly Soluble	−0.51	Soluble
F	−3.42	Soluble	−4.81	Moderately Soluble	−0.88	Soluble
G	−4.2	Moderately Soluble	−3.82	Soluble	−4.82	Moderately Soluble
H	−8.89	Poorly Soluble	−11.99	Insoluble	−9.08	Poorly Soluble
I	−6.5	Poorly Soluble	−10.4	Insoluble	−2.26	Soluble
J	−7.43	Poorly Soluble	−11.03	Insoluble	−5.1	Moderately Soluble

#### 4.2.4 Pharmacokinetics

In Table 6, for pharmacokinetics prediction, the gastrointestinal (GI) absorption rate was obtained higher for the compound G, while lower for all other compounds. All the compounds were found to be unable to cross the BBB barrier. The compounds A, E, F, G, H, I and J showed p-glycoprotein substrate activity, but the compounds B, C and D did not show any such activity. Among all the compounds, only the compounds C, D and H were identified as inhibitors of CYP2C9. All the compounds were observed to be non-inhibitors of CYP-1A2, CYP-2C19, CYP-2D6 and CYP-3A4. In case of skin permeation (log Kp, cm/s), higher negative value was obtained for Compound I (−11.13) and Compound H (−10.49) followed by Compound A and Compound E (−10.12), Compound F (−9.91), Compound J (−8.92), Compound G (−7.84), Compound C (−7.37), Compound B (−6.01) and lower for Compound D (−3.91), respectively.

**Table 6:** Predicted absorption, distribution, and metabolism parameters of 10 best candidates.

Compound Lists	A	B	C	D	E	F	G	H	I	J
**GI absorption**	Low	Low	Low	Low	Low	Low	High	Low	Low	Low
**BBB permeant**	No	No	No	No	No	No	No	No	No	No
**P-gp substrate**	Yes	No	No	No	Yes	Yes	Yes	Yes	Yes	Yes
**CYP-1A2**	No	No	No	No	No	No	No	No	No	No
**CYP-2C19**	No	No	No	No	No	No	No	No	No	No
**CYP-2C9**	No	No	Yes	Yes	No	No	No	Yes	No	No
**CYP-2D6**	No	No	No	No	No	No	No	No	No	No
**CYP-3A4**	No	No	No	No	No	No	No	No	No	No
**Log Kp (cm/s)**	−10.12	−6.01	−7.37	−3.91	−10.12	−9.91	−7.84	−10.49	−11.13	−8.92

#### 4.2.5 Drug likeness

From Table 7, we see that the compound G follows the Lipinski (Pfizer), Veber (GSK), Egan (Pharmacia) and Muegge (Bayer) filters and the compound D follows the Lipinski (Pfizer) and Veber (GSK) filters among all the compounds.

**Table 7:** Predicted drug likeliness parameters of 10 best candidates.

Compound Lists	Lipinski	Ghose	Veber	Egan	Muegge
**A**	No; 3 violations: MW>500, NorO>10, NHorOH>5	No; 2 violations: MW>480, MR> 130	No; 1 violation: TPSA>140	No; 1 violation: TPSA>131.6	No; 4 violations: MW>600, TPSA> 150, H-acc>10, H-don>5
**B**	No; 2 violations: MW>500, NHorOH>5	No; 2 violations: MW>480, MR>130	No; 1 violation: TPSA>140	No; 1 violation: TPSA>131.6	No; 3 violations: XLOGP3>5, TPSA> 150, H-don>5
**C**	No; 3 violations: MW>500, NorO>10, NHorOH>5	No; 4 violations: MW>480, MLOGP>5.6, MR>130, #atoms>70	No; 1 violation: TPSA>140	No; 2 violations: WLOGP>5.88, TPSA>131.6	No; 5 violations: MW>600, TPSA> 150, #rings>7, H-acc>10, H-don>5
**D**	Yes; 1 violation: MLOGP>4.15	No; 3 violations: MLOGP>5.6, MR>130, #atoms>70	Yes	No; 1 violation: WLOGP>5.88	No; 1 violation: XLOGP3>5
**E**	No; 3 violations: MW>500, NorO>10, NHorOH>5	No; 2 violations: MW>480, MR>130	No; 1 violation: TPSA>140	No; 1 violation: TPSA>131.6	No; 4 violations: MW>600, TPSA> 150, H-acc>10, H-don>5
**F**	No; 3 violations: MW>500, NorO>10, NHorOH>5	No; 4 violations: MW>480, WLOGP<−0.4, MR>130, #atoms>70	No; 1 violation: TPSA>140	No; 1 violation: TPSA>131.6	No; 3 violations: TPSA> 150, H-acc>10, H-don>5
**G**	Yes; 1 violation: MW>500	No; 2 violations: MW>480, MR>130	Yes	Yes	Yes
**H**	No; 3 violations: MW>500, NorO>10, NHorOH>5	No; 3 violations: MW>480, MR>130, #atoms>70	No; 2 violations: Rotors>10, TPSA>140	No; 1 violation: TPSA>131.6	No; 6 violations: MW>600, TPSA> 150, #rings>7, Rotors>15, H-acc>10, H-don>5
**I**	No; 3 violations: MW>500, NorO>10, NHorOH>5	No; 3 violations: MW>480, MR>130, #atoms>70	No; 1 violation: TPSA>140	No; 1 violation: TPSA>131.6	No; 5 violations: MW>600, TPSA> 150, #rings>7, H-acc>10, H-don>5
**J**	No; 3 violations: MW>500, NorO>10, NHorOH>5	No; 3 violations: MW>480, MR>130, #atoms>70	No; 1 violation: TPSA>140	No; 1 violation: TPSA>131.6	No; 4 violations: MW>600, TPSA> 150, H-acc>10, H-don>5

#### 4.2.6 Excretion

The total clearance of the compounds was found to be in the range between −2.333 (compound H) and 0.66 (compound B), indicating that the compound H will be retained for longer in the body and compound B will be excreted more rapidly. The compounds G and I were the only compounds predicted to be OCT2 substrates (Table 8).

**Table 8:** Prediction of Excretion parameters of 10 best candidates.

Compound lists	Total clearance	Renal OCT2 substate
A	0.401	No
B	0.66	No
C	0.412	No
D	0.146	No
E	0.401	No
F	−0.115	No
G	0.207	Yes
H	−2.333	No
I	0.191	Yes
J	−1.95	No

#### 4.2.7 Toxicity

All the compounds except B, C, H and I were found to be non-carcinogenic. *Salmonella typhimurium* reverse mutation assay (AMES) toxicity is a screening test to analyze whether a compound causes any mutation in bacteria *Salmonella typhimurium*. All the compounds were AMES negative, and no hepatotoxicity or skin sensitivity were observed for any of the compounds. The lethal dose (LD_50_) in rats was also analyzed (Table 9).

**Table 9:** Prediction of ADME/Toxicity parameters of 10 best candidates using PreADMET and pkCSM.

Toxicity Parameters	Toxic risk by PreADMET^1^ and pkCSM^2^									
	A	B	C	D	E	F	G	H	I	J
**Acute oral toxicity (rat- LD**_**50**_**) mol/kg**	2.492^2^	2.478^2^	2.482^2^	1.977^2^	2.492^2^	2.438^2^	2.067^2^	2.482^2^	2.482^2^	2.482^2^
**Chronic oral toxicity (rat)** **(log mg/kg_bw/day)**	5.95^2^	3.146^2^	6.402^2^	1.909^2^	5.95^2^	5.598^2^	2.058^2^	7.446^2^	10.22^2^	9.266^2^
**Max. tolerated dose** in **human (log mg/kg/day)**	0.438^2^	0.425^2^	0.438^2^	−0.202^2^	0.438^2^	0.418^2^	−0.466^2^	0.438^2^	0.438^2^	0.438^2^
**Carcinogenicity – mouse**	No^1^	No^1^	No^1^	No^1^	No^1^	No^1^	No^1^	Yes^1^	Yes^1^	No^1^
**Carcinogenecity - rat**	No^1^	Yes^1^	Yes^1^	No^1^	No^1^	No^1^	No^1^	Yes^1^	Yes^1^	No^1^
**Ames's test**	No^1,2^	No^1,2^	No^1,2^	No^1,2^	No^1,2^	No^1,2^	No^1,2^	No^1,2^	No^1,2^	No^1,2^
**Ames (TA100_10RLI)**	No^1^	No^1^	No^1^	No^1^	No^1^	No^1^	No^1^	No^1^	No^1^	No^1^
**Ames (TA100_NA)**	No^1^	No^1^	No^1^	No^1^	No^1^	No^1^	No^1^	No^1^	No^1^	No^1^
**Ames (TA1535_10RLI)**	No^1^	No^1^	No^1^	No^1^	No^1^	No^1^	No^1^	No^1^	No^1^	No^1^
**Ames (TA1535_NA)**	No^1^	No^1^	No^1^	No^1^	No^1^	No^1^	No^1^	No^1^	No^1^	No^1^
**hERG I inihibition**	Ambiguous^1^	Ambiguous^1^	Ambiguous^1^	Med risk^1^	Ambiguous^1^	Ambiguous^1^	Ambiguous^1^	Med risk^1^	Med risk^1^	High risk^1^
	No^2^	No^2^	No^2^	No^2^	No^2^	No^2^	No^2^	No^2^	No^2^	No^2^
**hERG II inihibition**	Yes^2^	Yes^2^	Yes^2^	Yes^2^	Yes^2^	Yes^2^	No^2^	Yes^2^	Yes^2^	Yes^2^
**Hepatotoxicity**	No^2^	No^2^	No^2^	No^2^	No^2^	No^2^	No^2^	No^2^	No^2^	No^2^
**Skin sensitization**	No^2^	No^2^	No^2^	No^2^	No^2^	No^2^	No^2^	No^2^	No^2^	No^2^
***T.Pyriformis* toxicity**	0.285^2^	0.285^2^	0.285^2^	0.366^2^	0.285^2^	0.285^2^	0.293^2^	0.285^2^	0.285^2^	0.285^2^

### 4.3 Protein-ligand interaction analysis

Thonningianin had its best docking value (−11.3 kcal/mol), forming just four conventional hydrogen bonds along with Lys51, Arg129 and Asp126 collaborating with the active sites. Hydrophobic interactions connect Lys51, Leu174, Phe119, as well as Trp127. In terms of the ten potential medications, the ligand cupressuflavone had the lowest binding energy (−9.9 kcal/mol), producing three conventional hydrogen bonds via the macromolecule ChREBP residues as well as five hydrophobic connections with the remaining residues of the protein Ile120, Trp127, Trp127 as well as Lys51 (Supplementary Table 3).

**Fig. 5:**
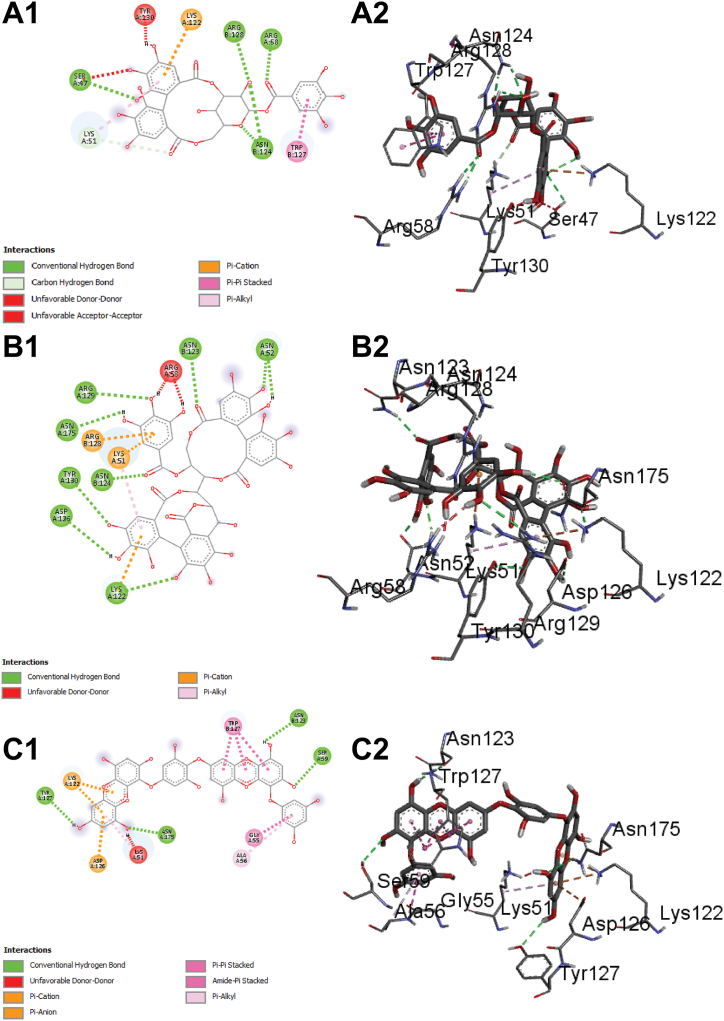

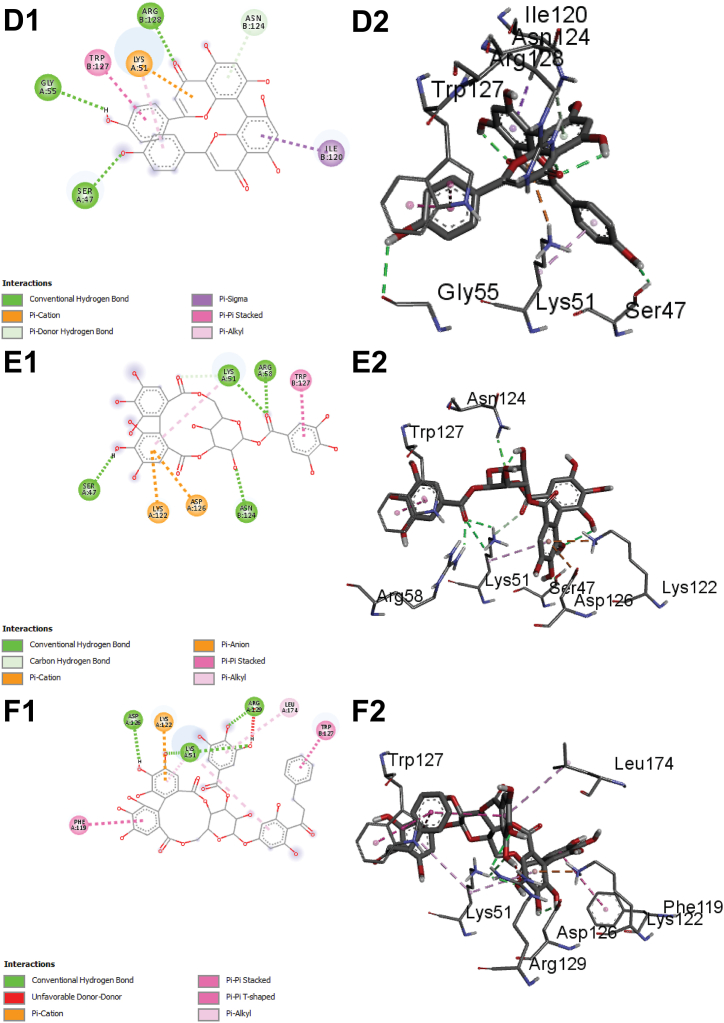

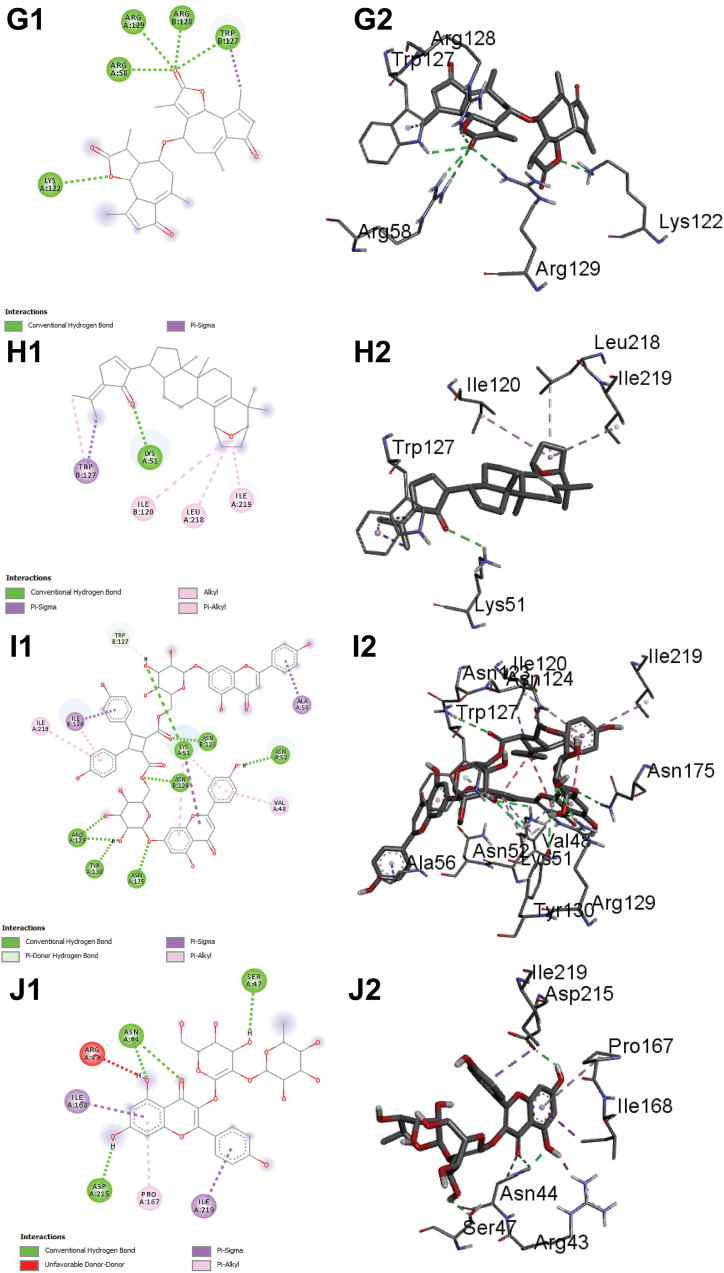
The interaction of all the selected ligand compounds with ChREBP protein. The left-sided figures exhibited the 3D structure, while the right-sided figures exhibited the 2D structure of the protein–ligand complex (A–C). Here, (A1 and A2) represented the 3D and 2D interaction of Corilagin, (B1 and B2)-compound Stachyurin, (C1 and C2)-compound Dieckol, (D1 and D2)-compound Cupressuflavone, and (E1 and E2)-compound Isocorilagin (F1 and F2)-compound Thonningianin A, (G1 and G2)-compound Lactucain, (H1 and H2)-compound Gypensapogenin A and (I1 and I2)-compound Stachysetin, (J1 and J2)-compound Kaempferol-3-rutinoside in interaction with the ChREBP protein complex.

Although gypensapogenin possesses lower binding affinity compared to isocorilagin, it includes more hydrogen bonds while interacting with the ChREBP. In addition to hydrogen bonds, hydrophobic bonds also contribute to the durability of the drug-ligand complex, referring an essential component of drug design techniques. Additionally, dieckol, lactucain, stachysetin, stachyurin, corilagin and kaempferol-3-rutinoside had noticeable binding affinities towards ChREBP (−10.8, −10.7, −10.4 and −10.4, −10.2, and −10.1 kcal/mol respectively) formed several hydrogen bonds and hydrophobic interactions (Fig. 5 A – C and Supplementary Table 3).

### 4.4 Reevaluation of docking score and binding energy calculation

The ten final selected compounds among total ligand library, their docking score range was −11.51 to −2.27, thonningianin, isocorilagin, stachysetin, stachyurin and dieckol have been shown the highest binding score compared to the other selected compounds. For all the ligands tested, all of the MMGBSA binding scores obtained were negative values in the high teens (Table 10). The higher the negative value of MMGBSA binding energy and the better the complex. Dieckol has the highest binding energy compared to other compounds. Overall ranking of binding energy was dieckol > stachysetin > stachyurin > kaempferol-3-rutinoside > corilagin > thonningianin A > isocorilagin > gypensapengenin A > cupressuflavone > lactucain A.

**Table 10:** Reevaluation of docking affinities and calculation of binding free energy (MMGBSA).

S.N.	Protein ID	Compound Name	Docking Affinity kcal/mol	Binding Free Energy (MMGBSA) kcal/mol
01	5F74	A	−2.27	−54.18656261
02		B	−5.75	−44.87631482
03		C	−10.31	−67.153184
04		D	−3.75	−46.67813874
05		E	−11.47	−50.37465542
06		F	−8.11	−54.34426573
07		G	−3.17	−40.97432398
08		H	−10.71	−61.03151821
09		I	−10.61	−55.35326775
10		J	−11.51	−51.46435531

### 4.5 MDS analysis

**Fig. 6:**
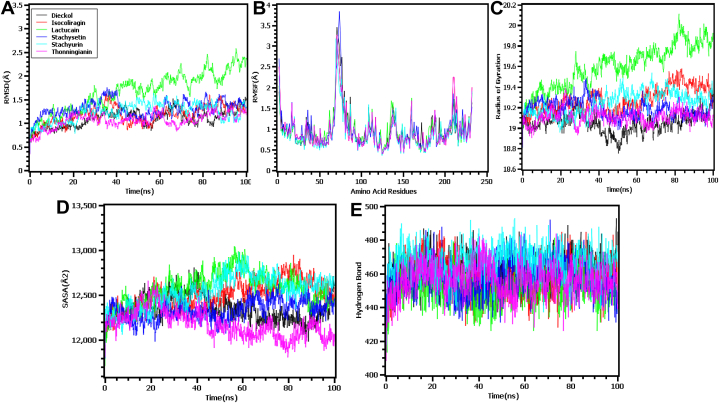
(A) The RMSD values for each ligand with the targeted receptor. (B) The RMSF values showing the atomic flexibility in terms of position. (C) Rg reflecting the compactness of all the protein. (D) SASA showing the surface area of all the provided molecules. (E) The number of H-bonds generated during the simulation.

In molecular dynamics simulation, the six top compounds, namely as the dieckol, isocorilagin, stachyurin, stachysetin and thonningianin with higher binder energy were selected to evaluate the stability of the complex and one compound with the lowest binding energy i.e., lactucain, was also taken for comparative study that indicates the significance of MMGBSA before simulation studies. After that, the molecular dynamics simulation study was carried out to investigates the structural deviations of the docked complexes across the simulation trajectories. The root mean square deviations of the C-alpha atoms obtained from the simulated complexes were explored to understand the conformational variations of the complexes. Fig. 6 (A) indicated the complexes that had initial upper trend in RMSD which might be responsible for the initial flexible nature of the complexes, but all of the complexes reached steady state after 20 ns times and had lower degree of deviations across the simulations periods which correlates with the structural stability. The complex lactucain had higher RMSD after 40 ns times and higher degree of deviations were observed till the rest of the times compared to the other complexes which indicates comparative flexible nature of the complexes. As a result, the complexes' RMSD was less than 2.5 Å throughout the simulated periods, indicating their stable nature.

Fig. 6 (D) shows that the complex isocoliragin, lactucain, stachyurin had a greater SASA, indicating that the complexes increased the surface area when the ligand bound. The remaining complexes exhibited stability and maintained lesser degrees of variations. The simulated trajectories reveal that the radius of gyration is an indicator of the dynamic character of the complexes. A larger Rg value corresponds to a greater mobility of the complexes, while a lower Rg value corresponds to a more stable nature of the complexes. Fig. 6 (C) shows that lactucain complexes have a larger Rg compared to other complexes, indicating their flexible character. The remaining complexes exhibited a lesser degree of departures, indicating the inherent stability of these complexes.

Fig. 6 (E) demonstrates that the complexes exhibit a stable trend in hydrogen bond patterning. An analysis was conducted on the root mean square variations of the complexes in order to gain insight into the flexibility of the amino acid residues. According to Fig. 6 (B), the maximal residues displayed a RMSF below 2.5 Å, indicating that the complexes were in a stable state.

Among the top five compounds, dieckol exhibited the lowest Binding Free Energy (MMGBSA) of approximately −67 kcal/mol, along with a docking affinity of −10.31 kcal/mol for the ChREBP receptor. In comparison, stachysetin showed a Binding Free Energy (MMGBSA) of about −61 kcal/mol and a docking affinity of −10.71 kcal/mol. Stachyurin, on the other hand, displayed a MMGBSA of around −55 kcal/mol with a docking affinity of −10.61 kcal/mol.

## 5 Discussion

The *in silico* drug design strategy has been gaining a lot of prominence in recent era due to the way it has demonstrated that it has the potential to speed up the discovery of new pharmaceuticals that are effective as well as safe. This is accomplished by analyzing the outcomes of pharmacophore screening, molecular docking, analysis of post-docking protein-ligand compounds interactions, MDS, and the development of noble and potential drug compounds against a broader spectrum of diseases in a simulation environment [10,56,57]. By analyzing the results of these processes used *in silico* drug design, the development of high-quality pharmaceuticals can be sped up significantly. This investigation made use of 20 FDA approved compounds and a total of 494 phytocompounds, top ten of which were selected specifically for their potential to act as lead compounds. ADMET profiling is an efficient method for cutting drug development costs by a significant amount and to perform "fact checks". Moreover, in case of high-performance assays, it provides secondary complementary judgments. The pharmacokinetic properties of these four phytochemicals were calculated using web-based servers provided by the pkCSM pharmacokinetics and Swiss ADME initiatives, as detailed in (Table 2).

Molecular docking is a technique used to predict the interactions that take place between molecules under circumstances that have the most stringent structural confirmation and the lowest binding affinity that is even conceivable. The bioactive phytocompounds with the most significant and potent binding score were determined with the help of the Maestro application (Schrödinger Release 2021-2: Maestro, Schrödinger, LLC, New York, NY, USA, 2020-3), which uses site-specific super molecular docking to assign a potential binding score. Corilagin, stachyurin, dieckol, isocorilagin, thonningianin, lactucain, gypensapogenin, stachysetin, kaempferol-3-rutinoside and cupressuflavone showed the better binding affinities (their 2D structures A1, B1, C1, D1, E1, F1, F1, G1, H1, I1 & J1(left) and 3D structures A2, B2, C2, D2, E2, F2, G2, H2. I2 & J2 (right) in Fig. 5 A–C) compared to the native ligand and FDA approved drugs**.**

Analysis of biomolecular interactions and the interface between protein structure and activity can empower the development of innovative pharmaceuticals. Additionally, MDS can help in the development of new therapeutics obtained from dynamic trajectory analysis as performance data [[58], [59], [60]]. In the context of our experiment, we made use of the Schrodinger package software known as the Desmond Application to carry out 100 ns MDS using the physiological and physicochemical parameters that were provided. This simulation trajectory was also utilized to analyze RMSD, Rg, RMSF, hydrogen bond number, and SASA perfectly [61]. The alpha carbon and backbone of the chosen 5F74 model protein were utilized to assess the protein structure's reliability and identify conformational changes; the lower value represents the more stable compound. RMSD values less than 1.5 are often indicative of stronger docking consistency, whereas RMSD values larger than 1.5 are typically indicative of average binding positions. According to the findings of our research, the RMSD values of protein-ligand interactions were within a suitable range, specifically the average mean values of 2 (the lowest value for the selected ligand compounds is round about 0.6, whereas the highest values are 2.6), indicating a more favorable docking position and along with no disruption of the protein-ligand structure (Fig. 6 A). The RMSF allows for the quantification of average protein fluctuations from a fixed reference point, additionally RMSF graphs show how these fluctuations are indicative of changes at the residue level, here Fig. 6 C represents the RMSF for C-alpha atoms of the protein.

This structural stability was evaluated by counting the total number of intermolecular bonds formed between the macromolecule and its ligand compounds. The ligand compound acarbose, which contains the highest number of intramolecular bonds and whose conformation is more stable than that of the ligand compound stachyurin, contained the highest number of intermolecular bonds (Fig. 6 E). Along with measuring the size shifts of the drug-like compounds, the SASA of the protein ligands was calculated using the simulated trajectories [62]. The proximity of hydrophobic amino acid residues to the water molecule contributes to structural instability, which in turn leads to a high SASA value [63]. And this is presented by Fig. 6 D. Moreover, the distance between the center of mass of the protein and its terminus is measured by Rg, it can tell you how far apart these two sections are. Rg is also a measurement of the distance between the protein's center of mass and its terminus. As a direct result of this, this measurement can quantify the compactness of the protein molecule and provides further details on the folding characteristics of the protein [64]. In addition, slackpacking is indicated when the Rg value is high, and compact packing is indicated when the Rg value is low [65]. Fig. 6 C presents a summary of the Rg values, which demonstrates that all compounds that contain protein exhibited standard compactness.

Dieckol compound is known to regulate blood glucose and lipid levels by improving insulin sensitivity in the diabetic condition, which is considered to be due to the activation of IRS-1/PI3K/Akt signaling [66]. Another study evidenced dieckol to prevent high glucose-induced β-cell damage [67]. This compound showed promising effect in human trials of dieckol at a concentration of 1.5 g/day for 12 weeks on pre-diabetic individuals, results of which revealed a substantial decrease in postprandial glucose levels without any adverse effects [68]. Stachysetin compound had previously been evidenced to have similar ligand-based similarity metrics against established antidiabetic agents [69]. Stachyurin revealed potent antidiabetic property upon stimulation of insulin-like glucose uptake [70].

In our study, the findings claimed that these compounds could be the potential therapeutic candidates for glucose-dependent carbohydrate-signaling gene regulator protein named ChREBP mediated NAFLD and T2DM. Additionally, our ADMET analysis revealed several concerns with the top compounds, including violations of drug similarity principles, potential carcinogenicity, and low absorption rates. To address these challenges, further structural optimization through Structure-Activity Relationship (SAR) studies is required. SAR analysis can guide the modification of these compounds to reduce potential toxicity and improve their pharmacokinetic properties, including absorption, thereby enhancing their suitability as drug candidates.

## 6 Conclusion and future directions

At present, there has been insufficient research on identifying modulators of ChREBP receptor which may result in exertion of lipogenic inhibition and anti-diabetic effects. This study is intended to provide a newer insight on targeting the novel ChREBP receptor and identifying novel therapies against liver disease and diabetes. In this work, 20 FDA approved and 494 natural compounds with known antidiabetic properties were screened using molecular docking analysis. Consequently, the top five compounds namely, dieckol, isocorilagin, stachyurin, stachysetin and thonningianin A were selected for simulations as they showed the best binding affinities and lowest binding energy with the target receptor. Their molecular simulations indicate that these could be potential leads in designing new therapies. These structures could be tailored by structure activity relationship studies leading to better pharmacokinetic properties and easy to formulate drugs for NAFLD and T2DM treatment.

Further research should be conducted focusing on the anti-lipogenic and antidiabetic activities of these compounds targeting ChREBP in animal models to validate the findings from this study. Corroboration between this *in silico* study along with further *in vivo* and *in vitro* studies would help provide a clearer understanding about the effects of modulation of this receptor on insulin sensitivity and resistance, which can be used to develop alternative therapeutic options for NAFLD and diabetes treatment.

## CRediT authorship contribution statement

**Hiron Saraj Devnath:** Writing – original draft, Data curation, Conceptualization. **Maisha Maliha Medha:** Writing – original draft, Data curation. **Md Naharul Islam:** Software, Data curation. **Partha Biswas:** Writing – original draft, Software. **Debasree Sen Oisay:** Formal analysis, Data curation. **Arafat Hossain:** Visualization, Software. **Rubaet Sharmin Ema:** Visualization, Data curation. **Md Mohaimenul Islam Tareq:** Visualization, Software. **Mimi Golder:** Supervision. **Md Nazmul Hasan:** Supervision. **Biswajit Biswas:** Writing – review & editing, Supervision, Conceptualization. **Samir Kumar Sadhu:** Writing – review & editing, Supervision, Conceptualization.

## Ethics approval and consent to participate

Not applicable.

## Availability of data and materials

The data are contained within the article.

## Consent for publication

Not applicable.

## Declaration of competing interest

The authors declare that they have no known competing financial interests or personal relationships that could have appeared to influence the work reported in this paper.
