# Supplementary material for: Bioactive small compounds effectively inhibit ChREBP overexpression to treat NAFLD and T2DM: A computational drug development approach
Source: Heliyon. 2025 Feb 10;11(4):e42477. doi: 10.1016/j.heliyon.2025.e42477 (PMC11872590; doi:10.1016/j.heliyon.2025.e42477)
Supplement: Multimedia component 1 [file mmc1.docx]

**Bioactive Small Compounds Effectively Inhibit ChREBP Overexpression to Treat Type 2 Diabetes Mellitus: A Computational Drug Development Approach**

**Hiron Saraj Devnath^1^, Maisha Maliha Medha^1^, Md. Naharul Islam^1^, Partha Biswas^2^, Debasree Sen Oisay^1^,** **Arafat Hossain^3^, Rubaet Sharmin Ema^1^, Md. Mohaimenul Islam Tareq^2^, Mimi Golder^1^,** **Md. Nazmul Hasan^2^, Biswajit Biswas^4*^, Samir Kumar Sadhu^1*^**

^1^Pharmacy Discipline, Khulna University, Khulna 9208, Bangladesh.

^2^Laboratory of Pharmaceutical Biotechnology and Bioinformatics, Department of Genetic Engineering and Biotechnology, Jashore University of Science and Technology, Jashore 7408, Bangladesh.

^3^Biochemistry and Molecular Biology Department, Life Science Faculty, Bangabandhu Sheikh Mujibur Rahman Science and Technology University, Gopalgonj 8100, Bangladesh.

^4^Department of Pharmacy, Faculty of Biological Science and Technology, Jashore University of Science and Technology, Jashore 7408, Bangladesh.

**^*^Corresponding Author:** Biswajit Biswas ([bb@just.edu.bd](mailto:bb@just.edu.bd))

Samir Kumar Sadhu ([sksadhu1969@pharm.ku.ac.bd](mailto:sksadhu1969@ku.ac.bd))

**Supplementary Table 1:** Binding affinities of the FDA approved drugs docked with ChREBP

| **Sl No.** | **Pubchem ID** | **Drug** | **Binding affinity** | **Class** | **Mechanism** |
| --- | --- | --- | --- | --- | --- |
| 1 | 3488 | Glyburide | -8.1 | SU | Close ATP-sensitive K-channels in the beta-cell plasma membrane results in insulin secretion |
| 2 | 3478 | Glipizide | -8.9 |  |  |
| 3 | 2727 | Chlorpropamide | -6.5 |  |  |
| 4 | 5505 | Tolbutamide | -6.7 |  |  |
| 5 | 5503 | Tolazamide | -7.7 |  |  |
| 6 | 41774 | Acarbose | -7.5 | Alpha glucosidase inhibitors | Prevent the digestion of carbohydrates, improve glycemic control |
| 7 | 441314 | Miglitol | -5.1 |  |  |
| 8 | 77999 | Rosiglitazone | -7.1 | TZD | activation of the gamma isoform of the peroxisome proliferator-activated receptor (PPAR gamma) |
| 9 | 4829 | Pioglitazone | -7.7 |  |  |
| 10 | 4091 | Metformin | -3.9 | Biguanide | Inhibit gluconeogenesis, insulin sensitizer |
| 11 | 4369359 | Sitagliptin | -7.6 | DPP-4 inhibitors | Block the action of the enzyme DPP-4 |
| 12 | 11243969 | Saxagliptin | -7.1 |  |  |
| 13 | 10096344 | Linagliptin | -9.8 |  |  |
| 14 | 11450633 | Alogliptin | -7.3 |  |  |
| 15 | 65981 | Repaglinide | -7.7 | Meglitinides | inhibition of the adenosine triphosphate (ATP)-dependent potassium channel |
| 16 | 31101 | Bromocriptine | -9.8 | Dopamine receptor agonist | Reduce plasma glucose, insulin, resistance, triglycerides, Free Fatty Acid (FFA) levels |
| 17 | 24812758 | Canagliflozin | -9.2 | SGLT2 inhibitors | Increase insulin secretion, suppress glucose reabsorption |
| 18 | 9887712 | Dapagliflozin | -8.1 |  |  |
| 19 | 11949646 | Empagliflozin | -8.5 |  |  |
| 20 | 44814423 | Ertuglifozin | -8.3 |  |  |

**Supplementary Table 2:** Nature compounds docked with ChREBP listed here

| Serial No | Bioactive Compound | Source | Used Parts | Bioactive methods | Binding Affinity | Reference |
| --- | --- | --- | --- | --- | --- | --- |
| 1 | (-)-Epicatechin | *Embelia ribes (Myrsinaceae)* | Leaves | α-Glucosidase | -7.6 | (Dang et al., 2015) |
| 2 | (+)-Lariciresinol | Edgeworthia gardneri  (Thymelaeaceae) | Flowers | α -Glucosidase | -7.1 | (Ma et al., 2015) |
| 3 | (+)-Syringaresinol | Embelia ribes  (Myrsinaceae) | Leaves | α -Glucosidase | -7.8 | (Dang et al., 2015) |
| 4 | (+)-Syringaresinol-beta-D-glucoside | Embelia ribes  (Myrsinaceae) | Leaves | α -Glucosidase | -7.9 | (Dang et al., 2015) |
| 5 | (+)-Taxifolin | Embelia ribes  (Myrsinaceae) | Leaves | α -Glucosidase | -7.9 | (Dang et al., 2015) |
| 6 | 1,5-Dicaffeoylquinic acid | Acanthopanax senticosus  (Araliaceae) | Leaves | α -Glucosidase | -8.2 | (Zhou et al., 2012) |
| 7 | 2,3-Dihydroauriculatin | Erythrina addisoniae (Leguminosae) | Stem bark | PTP1B | -8.8 | (Bae et al., 2006) |
| 8 | 2,4,6-trihydroxybenzoic acid | Zea mays (Poaceae) | Kernels | α -Glucosidase | -6 | (Nile & Park, 2014) |
| 9 | 2',4'-dihydroxy-4-methoxydihydrochalcone | Artemisia dracun culus (Asteraceae) | Seeds | H4IIE cells | -7 | (Govorko et al., 2007) |
| 10 | 3'- methoxyhirsutrin | Zea mays (Poaceae) | Kernels | α -Glucosidase | -7.4 | (Nile & Park, 2014) |
| 11 | 3,4-Dicaffeoylquinic acid | Acanthopanax senticosus  (Araliaceae) | Leaves | α -Glucosidase | -8.4 | (Zhou et al., 2012) |
| 12 | 3,4-Dihydroxy benzoic acid | Echinochloa utilis (Poaceae) | Seeds | α -Glucosidase Caco-2 cells | -5.5 | (Seo et al., 2015a) |
| 13 | 3,5-Dicaffeoylquinic acid methyl ester | Artemisia  capillaris (Asteraceae) | Whole plant | α -Glucosidase PTP1B | -9.1 | (Nurul Islam et al., 2013) |
| 14 | Trans-3,5-O-dicaffeoylquinic acid | Acanthopanax senticosus (Araliaceae) | Leaves | α-Glucosidase | -9.2 | (Zhou et al., 2012) |
| 15 | 3',5'-Diprenylgenistein | Tetracera scandens  (Dilleniaceae) | Branch | L6 cells PTP1B | -8.4 | (M. S. Lee et al., 2009) |
| 16 | 3-beta-Hydroxyetio-17-beta-dammaranic acid | Gynostemma pentaphyllum (Cucurbitaceae) | Aerial parts | PTP1B | -7.9 | (Zhang et al., 2013) |
| 17 | 3-Caffeoylquinic acid | Ilex kudingcha  (Aquifoliaceae) | Leaves | α-Glucosidase | -7.9 | (Xu et al., 2015) |
| 18 | 3-epicorosolic acid | Crataegus pinnatifida (Rosaceae) | Fruits | α-Glucosidase PTP1B | -8.6 | (Chowdhury et al., 2014) |
| 19 | 4,5-Dicaffeoylquinic acid | Acanthopanax senticosus (Araliaceae) | Leaves | α-Glucosidase | -8.3 | (Zhou et al., 2012) |
| 20 | 4-Caffeoylquinic acid | Ilex kudingcha (Aquifoliaceae) | Leaves | α-Glucosidase | -7.8 | (Xu et al., 2015) |
| 21 | 4-Hydroxybenzaldehyde | Edgeworthia gardneri (Thymelaeaceae | Flowers | α-Glucosidase | -5 | (Ma et al., 2015) |
| 22 | 4-Hydroxyderricin | Angelica keiskei (Apiaceae) | Stems | 3T3-L1 cells | -7.7 | (Ohta et al., 2015) |
| 23 | 5-caffeoylquinic acid | Holarrhena antidysenterica  (Apocynaceae) | Seeds | α-Glucosidase α-Amylase | -8 | (D. Kumar, S. Datta, et al., 2013) |
| 24 | 6,8-Diprenylgenistein | Erythrina senegalensis (Fabaceae) | Stem bark | DGAT 1 | -8.5 | (Oh et al., 2009) |
| 25 | 6-Demethoxycapillarisin | Artemisia dracunculus (Asteraceae) | Seeds | H4IIE cells | -7.9 | (Govorko et al., 2007) |
| 26 | 7-Methoxyheptaphylline | Clausena harmandiana (Rutaceae) | Roots | L6 cells | -7.6 | (Noipha et al., 2010) |
| 27 | 8-Methoxyeriodictyol | Iris unguicularis (Irideaceae) | Whole plant | α-Glucosidase | -8 | (Mosihuzzman et al., 2013) |
| 28 | 8-oxo-berberine | Berberis brevíssima Berberis parkeriana (Berberidaceae) | Roots | PTP1B | -8.6 | (Ali et al., 2013) |
| 29 | 23-hydroxyursolic acid | Lagerstroemia speciosa (Lythraceae) | Leaves | α-Glucosidase α-Amylase | -8.5 | (Hou et al., 2009) |
| 30 | 24-Methylenepollinastanol | Holarrhena curtisii  (Apocynaceae) | Seedpods | α-Glucosidase | -7.8 | (Srisurichan & Pornpakakul, 2015) |
| 31 | 30-Norhederagenin | Paeonia suffruticosa (Paeoniaceae) | Root barks | HepG2 cells | -8.1 | (Ha do et al., 2009) |
| 32 | Abyssinone-IV | Erythrina mildbraedii (Leguminosae) | Root bark | PTP1B | -8.4 | (Na et al., 2006) |
| 33 | Abyssinone-IV-4'-O-methyl ether | Erythrina mildbraedii (Leguminosae) | Root bark | PTP1B | -8.2 | (Na et al., 2006) |
| 34 | Abyssinone-V | Erythrina mildbraedii (Leguminosae) | Root bark | PTP1B | -8.5 | (Na et al., 2006) |
| 35 | Abyssinone-V-4'-O-methyl ether | Erythrina mildbraedii (Leguminosae) | Root bark | PTP1B | -8.4 | (Na et al., 2006) |
| 36 | Acteoside | Phlomis tuberosa (Lamiaceae) | Roots | α-Glucosidase | -8.7 | (Yang et al., 2015) |
| 37 | Aculeatin | Toddalia asiática (Rutaceae) | Stem | 3T3-L1 cells | -7.1 | (Watanabe et al., 2014) |
| 38 | Afzelin | Embelia ribes (Myrsinaceae) | Leaves | α-Glucosidase | -8.2 | (Dang et al., 2015) |
| 39 | Albafuran A | Morus alba var. tatarica (Moraceae) | Root barks | α-Glucosidase PTP1B | -7.3 | (Zhang et al., 2014) |
| 40 | Albafuran B | Morus alba var. tatarica (Moraceae) | Root barks | α-Glucosidase PTP1B | -7.7 | (Zhang et al., 2014) |
| 41 | Albanin A | Morus alba var. tatarica (Moraceae) | Root barks | α-Glucosidase | -7.8 | (Y. L. Zhang et al., 2015) |
| 42 | Albanin D | Morus alba var. tatarica (Moraceae) | Root barks | α-Glucosidase | -8.7 | (Y. L. Zhang et al., 2015) |
| 43 | alpha-Mangostin | Garcinia cowa (Clusiaceae) | Flowers | α-Glucosidase | -8.1 | (Sriyatep et al., 2015) |
| 44 | Alpinumisoflavone | Tetracera scandens (Dilleniaceae) | Branch | L6 cells PTP1B | -9.1 | (M. S. Lee et al., 2009) |
| 45 | Selaginellin | Selaginella tamariscina (Selaginellaceae) | Aerial parts | PTP1B | -8 | (P. H. Nguyen, B. T. Zhao, et al., 2015) |
| 46 | Anhydroaegeline | Aegle marmelos (Rutaceae) | Leaves | α-Glucosidase | -7.1 | (Phuwapraisirisan et al., 2008) |
| 47 | Arjunolic acid | Lagerstroemia speciosa (Lythraceae) | Leaves | α-Glucosidase α-Amylase | -8.6 | (Hou et al., 2009) |
| 48 | Asiatic acid | Lagerstroemia speciosa (Lythraceae) | Leaves | α-Glucosidase α-Amylase | -8 | (Hou et al., 2009) |
| 49 | Auriculatin | Erythrina senegalensis (Fabaceae) | Stem bark | DGAT 1 | -8.2 | (Oh et al., 2009) |
| 50 | Avicularin | Mimosa  pudica  (Fabaceae) | Leaves | α-Glucosidase α-Amylase | -7.7 | (Tasnuva et al., 2019) |
| 51 | Azadiradione | Azadirachta indicia (Meliaceae) | Fruits | α-Amylase | -9 | (Ponnusamy et al., 2015) |
| 52 | Bakuchiol | Psoralea corylifolia (Leguminosae) | Seeds | PTP1B | -6.2 | (Kim et al., 2005) |
| 53 | Benzoic acid | Vaccinium vitisidaea (Ericaceae) | Fruits | AMPK | -5.4 | (Eid et al., 2010) |
| 54 | β-Amyrin acetate | Holarrhena curtisii (Apocynaceae) | Seedpods | α-Glucosidase | -9 | (Srisurichan & Pornpakakul, 2015) |
| 55 | β-Mangostin | Garcinia cowa (Clusiaceae) | Flowers | α-Glucosidase | -7.6 | (Sriyatep et al., 2015) |
| 56 | Biochanin A | Pueraria thunbergiana (Leguminosae) | Roots | α-Glucosidase  α -Amylase | -8.4 | (Park et al., 2009) |
| 57 | Butein | Andromachia igniaria (Asteraceae) | Aerial parts | α -Amylase α-Glucosidase | -7.3 | (Saltos et al., 2015) |
| 58 | Calein C | Brickellia cavanillesii (Asteraceae) | Aerial parts | α-Glucosidase | -7.8 | (Escandón-Rivera et al., 2012) |
| 59 | Calycosin | *Pueraria lobata* (Leguminosae) | Roots | α-Glucosidase  PTP1B | -7.8 | (Seong et al., 2016) |
| 60 | Cardamonin | Alpinia katsu madai (Zingiberaceae) | Seeds | L6 cells | -7.5 | (Yamamoto et al., 2011) |
| 61 | Cassigarol E | Cyperus rotundus (Cyperaceae) | Rhizomes | α-Glucosidase α-Amylase | -8.7 | (Tran et al., 2014) |
| 62 | Caulophyllogenin | Kalopanax pictus (Araliaceae) | Stem Bark | HepG2 cells | -8.4 | (Quang et al., 2011) |
| 63 | Chaplupyrrolidone A | Piper sarmentosum (Piperaceae) | Leaves | α-Glucosidase | -7.5 | (Damsud et al., 2013) |
| 64 | Chaplupyrrolidone B | Piper sarmentosum (Piperaceae) | Leaves | α-Glucosidase | -7.1 | (Damsud et al., 2013) |
| 65 | Charantoside B | Momordica charantia (Cucurbitaceae) | Fruits | α-Glucosidase | -8.3 | (Nhiem et al., 2010) |
| 66 | Chebulagic acid | Terminalia chebula (Combretaceae) | Fruits | α-Glucosidase | -9.1 | (Gao et al., 2007) |
| 67 | Chebulanin | Terminalia chebula (Combretaceae) | Fruits | α-Glucosidase | -9.7 | (Gao et al., 2007) |
| 68 | Chrysophanol | Rheum sp. (Polygonaceae) | Rhizome | L6 cells 32DIR cells PTP1B | -8.2 | (Lee & Sohn, 2008) |
| 69 | Cinchonain Ib | Eriobotrya japonica (Rosaceae) | Leaves | INS-1 cells | -8.3 | (Qa'dan et al., 2009) |
| 70 | Coixol | Scoparia dulcis (Scrophulariceae | Whole plant | Mouse pancreas cells MIN-6 cells | -5.7 | (Sharma et al., 2015) |
| 71 | Columbamine | Berberis brevíssima Berberis parkeriana (Berberidaceae) | Roots | PTP1B | -7.6 | (Ali et al., 2013) |
| 72 | Continentalic acid | Aralia continentalis (Araliaceae) | Roots | Rat Lens PTP1B | -7.4 | (Jung et al., 2012) |
| 73 | Coptisine | Coptis chinensis (Ranunculaceae) | Rhizomes | HepG2 cells | -9.2 | (Chen et al., 2012) |
| 74 | Corilagin | Terminalia macroptera (Combretaceae) | Leaves | α-Glucosidase | -10.2 | (Pham et al., 2014) |
| 75 | Corosolic acid | Lagerstroemia speciosa (Lythraceae) | Leaves | α-Glucosidase α-Amylase | -8.6 | (Hou et al., 2009) |
| 76 | Cosmosiin | Citrus grandis (Rutaceae) | Leaves | 3T3-L1 cells | -8.2 | (Rao et al., 2011) |
| 77 | Cuminaldehyde | Cuminum cyminum (Apiaceae) | Seeds | α-Glucosidase Rat lens | -5.6 | (Lee & chemistry, 2005) |
| 78 | Cuminol | Cuminum cyminum (Apiaceae) | Seeds | Rat pancreatic cells | -5.5 | (Patil et al., 2013) |
| 79 | Cupressuflavone | Selaginella tamariscina (Selaginellaceae) | Aerial parts | PTP1B | -9.9 | (P. H. Nguyen, B. T. Zhao, et al., 2015) |
| 80 | Daidzein | Pueraria lobata (Leguminosae) | Roots | α-Glucosidase  PTP1B | -7.6 | (Seong et al., 2016) |
| 81 | Daphnoretin | Edgeworthia gardneri (Thymelaeaceae) | Flowers | α-Glucosidase α-Amylase | -9.2 | (Zhao et al., 2015) |
| 82 | Dehydrodieugenol B | Syzygium aromaticum (Myrtaceae) | Flowers | CV-1 cells 3T3-L1 cells | -7.1 | (Kuroda et al., 2012) |
| 83 | Dehydrodieugenol | Syzygium aromaticum (Myrtaceae) | Flowers | CV-1 cells 3T3-L1 cells | -7 | (Kuroda et al., 2012) |
| 84 | Dehydropipernonaline | Piper nigum (Piperaceae) | Fruits | Microsome rat liver | -7.2 | (Lee et al., 2006) |
| 85 | Deoxy-andrographolide | Andrographis paniculata (Acanthaceae) | Whole plant | ↓ OGTT ↓ Insulin | -7.5 | (Arha et al., 2015) |
| 86 | Derrone | Erythrina senegalensis (Fabaceae) | Stem bark | DGAT 1 | -8.7 | (Oh et al., 2009) |
| 87 | D-Glucono-1,4-lactone | Rosa canina (Rosaceae) | Fruits | α-Glucosidase | -5.6 | (Behvar Asghari et al., 2015) |
| 88 | Diasesartemin | Commiphora wightii (Burseraceae) | Resin | α-Glucosidase | -7.4 | (El-Mekkawy et al., 2013) |
| 89 | Dihydroauroglaucin | Phlomis tuberosa (Lamiaceae) | Roots | α-Glucosidase | -6.7 | (Yang et al., 2015) |
| 90 | Dihydroisotanshinone I | Salvia miltiorrhiza (Lamiaceae) | Roots | PTP1B | -9.5 | (Han et al., 2005) |
| 91 | Dihydromyricetin | Impatiens balsamina (Balsaminaceae) | Flowers | α-Glucosidase | -7.9 | (Q. Li et al., 2015) |
| 92 | Dodeca-2E,4E-dienoic acid isobutylamide | Echinacea purpurea (Asteraceae) | Flowers | 3T3-L1 cells | -5.2 | (Christensen et al., 2009) |
| 93 | Edgeworin | Edgeworthia gardneri (Thymelaeaceae) | Flowers | α-Glucosidase α-Amylase | -9.2 | (Zhao et al., 2015) |
| 94 | Edgeworthin | Edgeworthia gardneri (Thymelaeaceae | Flowers | α-Glucosidase α-Amylase | -9 | (Zhao et al., 2015) |
| 95 | Emodin | Rheum emodi (Polygonaceae) | Rhizomes | α-Glucosidase | -8 | (Arvindekar et al., 2015) |
| 96 | Enterodiol | Linum usitatissimum (Linaceae) | Seeds | α-Glucosidase α-Amylase | -5.9 | (Hano et al., 2013) |
| 97 | Enterolactone | Linum usitatissimum (Linaceae) | Seeds | α-Glucosidase α-Amylase | -7.4 | (Hano et al., 2013) |
| 98 | Epiberberine | Coptis chinensis (Ranunculaceae) | Rhizomes | PTP1B | -8.1 | (Choi et al., 2015) |
| 99 | Epi-lupeol | Euonymus alatus (Celastraceae) | Corks | PTP1B α-Glucosidase | -8.7 | (S. Y. Jeong et al., 2015) |
| 100 | Eriodictyol | Andromachia igniaria (Asteraceae) | Aerial parts | α-Amylase  α-Glucosidase | -8 | (Saltos et al., 2015) |
| 101 | Erylatissin C | Erythrina abyssinica (Leguminosae) | Stem bark | PTP1B Chinese hamster ovary cells | -8.5 | (Cui et al., 2010) |
| 102 | Erypoegin F | Erythrina abyssinica (Leguminosae) | Stem bark | C2C12 cells | -7.3 | (Nguyen et al., 2010) |
| 103 | Erysenegalensein D | Erythrina senegalensis (Fabaceae) | Stem bark | DGAT 1 | -7.9 | (Oh et al., 2009) |
| 104 | Erysenegalensein O | Erythrina senegalensis (Fabaceae) | Stem bark | DGAT 1 | -9.1 | (Oh et al., 2009) |
| 105 | Erythribyssin F | Erythrina abyssinica (Leguminosae) | Stem bark | C2C12 cells | -8.1 | (Nguyen et al., 2010) |
| 106 | Esculetin | Artemisia capillaris (Asteraceae) | Whole plant | α-Glucosidase PTP1B | -6.1 | (Islam et al., 2013a) |
| 107 | Ethyl caffeate | Cirsium japonicum var. australe (Asteraceae) | Whole plant | 3T3-L1 cells | -6 | (Lai et al., 2014) |
| 108 | Euphol | Euphorbia kansui (Euphorbiaceae) | Roots | Mice and Hu man’s 11-HSD1 and 11-HSD2 | -8.1 | (Guo et al., 2012) |
| 109 | Europetin-3-O-rhamnoside | Syzygium aqueum (Myrtaceae) | Leaves | 3T3-L1 cells | -8.1 | (Manaharan et al., 2013) |
| 110 | Excelside B | Fraxinus excelsior (Oleaceae) | Seeds | 3T3-L1 cells HEK-293 cells | -8.3 | (Naisheng Bai et al., 2010) |
| 111 | Falcarindiol | *Oenanthe javanica* (Apiaceae) | Aerial roots | GSK-3β | -5.3 | (Yoshida et al., 2013) |
| 112 | Falcarinol | Daucus carota (Apiaceae) | Roots | 3T3-L1 cells Pig muscles | -4.9 | (El-Houri et al., 2015) |
| 113 | Feruloyl serotonin | Echinochloa utilis (Poaceae) | Seeds | α-Glucosidase Caco-2 cells | -7.9 | (Seo et al., 2015c) |
| 114 | Flavoglaucin | Phlomis tuberosa (Lamiaceae) | Roots | α-Glucosidase | -6.1 | (Yang et al., 2015) |
| 115 | Formononetin | Dalbergia odorífera (Leguminosae) | Heartwood | α-Glucosidase | -7.6 | (Choi et al., 2010) |
| 116 | Isocorilagin | Punica granatum (Punicaceae) | Flowers | α-Glucosidase | -10.6 | (Yuan et al., 2012) |
| 117 | Imperatorin | Ducrosia anethifolia (Apiaceae) | Stems Leaves | α-Glucosidase α-Amylase α-galactosidase | -8.1 | (Shalaby et al., 2014) |
| 118 | Hederacolchiside F | Rosa rugosa (Rosaceae) | Roots | Sucrase | -8.6 | (Thao, Luyen, Jo, et al., 2014) |
| 119 | Ilekudinol B | Weigela subsessilis (Caprifoliaceae) | Leaves | L6 cells | -8.6 | (Thao, Luyen, Jo, et al., 2014) |
| 120 | Gedunin | Azadirachta indi cia (Meliaceae) | Fruits | α-Amylase AR42J cells | -9.1 | (Ponnusamy et al., 2015) |
| 121 | Kaempferol-3-rutinoside | Edgeworthia gardneri (Thymelaeaceae) | Flowers | α-Glucosidase | -10.1 | (Ma et al., 2015) |
| 122 | Hirsutrin | Zea mays (Poaceae) | Kernels | α-Glucosidase | -7.9 | (Nile & Park, 2014) |
| 123 | Isookanin | Albizzia Lebbeck (Mimosaceae) | Barks | α-Glucosidase α-Amylase | -8.3 | (Ahmed et al., 2014) |
| 124 | Kajiichigoside F1 | Potentilla fulgens (Rosaceae) | Roots | α-Glucosidase | -9.6 | (D. Kumar, R. Ghosh, et al., 2013) |
| 125 | Icariside F2 | Piper retrofractum (Piperaceae) | Leaves | α-Glucosidase | -7.9 | (Luyen et al., 2014) |
| 126 | Hyptadienic acid | Potentilla fulgens (Rosaceae) | Roots | α-Glucosidase | -8.1 | (D. Kumar, R. Ghosh, et al., 2013) |
| 127 | Kuwanon T | Morus alba var. tatarica (Moraceae) | Root barks | α-Glucosidase | -7.6 | (Ya‐Long Zhang et al., 2015) |
| 128 | Jaboticabin | Morus alba (Moraceae) | Fruits | α-Glucosidase | -7.4 | (Wang et al., 2013) |
| 129 | Isosojagol | Erythrina abyssinica (Leguminosae) | Stem bark | C2C12 cells | -8.6 | (Nguyen et al., 2010) |
| 130 | Kansenone | Euphorbia kansui (Euphorbiaceae) | Roots | Mice and Hu man’s 11-HSD1 and 11-HSD2 | -7.9 | (Guo et al., 2012) |
| 131 | Khellactone | Angelica keiskei (Apiaceae) | Aerial parts | α-Glucosidase | -7.4 | (Luo et al., 2012) |
| 132 | Kotalanol | Salacia chinensis (Hippocrateaceae) | Stems | α-Glucosidase | -6.5 | (Morikawa et al., 2015) |
| 133 | Isorhamnetin-3-glycoside | Phlomis stewartii (Lamiaceae) | Whole plant | α-Glucosidase | -8 | (Jabeen et al., 2013) |
| 134 | Ginnalin B | Acer pycnanthum (Aceraceae) | Leaves | α-Glucosidase | -7.5 | (Ogawa et al., 2011) |
| 135 | Ginnalin C | Acer pycnanthum (Aceraceae) | Leaves | α-Glucosidase | -8.4 | (Ogawa et al., 2011) |
| 136 | Kuwanon U | Morus alba var. tatarica (Moraceae) | Root barks | α-Glucosidase | -9.2 | (Y. L. Zhang et al., 2015) |
| 137 | Glycybenzofuran | Glycyrrhiza uralensis (Leguminosae) | Roots | PTP1B | -8.2 | (Li et al., 2010) |
| 138 | Isoquercetin | Edgeworthia gardneri (Thymelaeaceae) | Flowers | α-Glucosidase | -7.4 | (Ma et al., 2015) |
| 139 | Isobavachalcone | Angelica keiskei (Apiaceae) | Aerial parts | α-Glucosidase | -7.6 | (Luo et al., 2012) |
| 140 | Hispidulin | Scoparia dulcis (Scrophulariaceae) | Aerial parts | α-Glucosidase HEK-293 cells | -7.7 | (Liu et al., 2014) |
| 141 | Hyperoside | Artemisia capillaris (Asteraceae) | Whole plant | α-Glucosidase PTP1B | -7.7 | (Islam et al., 2013a) |
| 142 | Isorhamnetin | Artemisia capillaris (Asteraceae) | Whole plant | α-Glucosidase PTP1B | -7.8 | (Islam et al., 2013a) |
| 143 | Heptaphylline | Clausena harmandiana (Rutaceae) | Roots | L6 cells | -7.7 | (Noipha et al., 2010) |
| 144 | Ginnalin A | Acer pycnanthum (Aceraceae) | Leaves | α-Glucosidase | -8.9 | (Ogawa et al., 2011) |
| 145 | Isorhamnetin-3-O-glucoside | Salsola kali (Chenopodiaceae) | Aerial parts | α-Amylase | -7.4 | (Tundis et al., 2007) |
| 146 | Kaempferol 3-neohesperidoside | Cyathea phalerata (Cyatheaceae) | Stems | Rat soleus muscle | -7.5 | (Zanatta et al., 2008) |
| 147 | gamma-Mangostin | Garcinia mangostan (Clusiaceae) | Seedcases | α-Glucosidase | -8.2 | (Ryu et al., 2011) |
| 148 | Guaijaverin | Psidium guajava (Myrtaceae) | Leaves | α-Glucosidase α-Amylase | -7.9 | (Wang et al., 2010) |
| 149 | Isorhamnetin-3-O-rutinoside | Salsola kali (Chenopodiaceae) | Aerial parts | α-Amylase | -7.9 | (Tundis et al., 2007) |
| 150 | Glisoflavone | Glycyrrhiza uralensis (Leguminosae) | Roots | PTP1B | -8 | (Li et al., 2010) |
| 151 | Gypensapogenin B | Gynostemma pentaphyllum (Cucurbitaceae) | Aerial parts | PTP1B | -9.9 | (Zhang et al., 2013) |
| 152 | Gypensapogenin A | Gynostemma pentaphyllum (Cucurbitaceae) | Aerial parts | PTP1B | -10.2 | (Zhang et al., 2013) |
| 153 | Isooxypeucedanin | Ducrosia anethifolia (Apiaceae) | Stems Leaves | α-Glucosidase α-Amylase β-galactosidase | -7.2 | (Shalaby et al., 2014) |
| 154 | Isotanshinone IIA | Salvia miltiorrhiza (Lamiaceae) | Roots | PTP1B | -9.7 | (Han et al., 2005) |
| 155 | Isocryptotanshinone | Salvia miltiorrhiza (Lamiaceae) | Roots | PTP1B | -9.1 | (Han et al., 2005) |
| 156 | Guggulsterone E | Commiphora mukul (Burseraceae) | Resin | ↑ PPARα ↑ GLUT4  ↑ Glycogen | -8.9 | (B. Sharma et al., 2009) |
| 157 | Isoacteoside | Phlomis tuberosa (Lamiaceae) | Roots | α-Glucosidase | -7.8 | (Yang et al., 2015) |
| 158 | Isohydnocarpin | Hydnocarpus wightiana (Achariaceae) | Seeds | α-Glucosidase N-Acetyl- β-D-glucosaminidase | -8.6 | (Reddy et al., 2005) |
| 159 | Gypensapogenin E | Gynostemma pentaphyllum (Cucurbitaceae) | Aerial parts | PTP1B | -8.7 | (Zhang et al., 2013) |
| 160 | Gypensapogenin G | Gynostemma pentaphyllum (Cucurbitaceae) | Aerial parts | PTP1B | -9 | (Zhang et al., 2013) |
| 161 | Honokiol | Magnolia obovata (Magnoliaceae) | Barks | 3T3-L1 cells | -7.2 | (Choi et al., 2011) |
| 162 | Kaurenoic acid | Aralia continentalis (Araliaceae) | Roots | Rat Lens PTP1B | -7.7 | (Jung et al., 2012) |
| 163 | Hederagenin | Kalopanax pictus (Araliaceae) | Stem Bark | HepG2 cells | -8.4 | (Quang et al., 2011) |
| 164 | Hyperin | Acanthopanax senticosus (Araliaceae) | Leaves | α-Glucosidase | -7.6 | (Zhou et al., 2012) |
| 165 | Glutinol | Scoparia dulcis (Scrophulariaceae) | Whole plant | Mouse pancreas cells MIN-6 cells | 8.8 | (Sharma et al., 2015) |
| 166 | Licochalcone A | Glycyrrhiza uralensis (Leguminosae) | Rhizomes | α-Glucosidase PTP1B | -7.4 | (Guo et al., 2015) |
| 167 | Licochalcone B | Glycyrrhiza uralensis (Leguminosae) | Rhizomes | α-Glucosidase PTP1B | -7.3 | (Guo et al., 2015) |
| 168 | Licochalcone C | Glycyrrhiza uralensis (Leguminosae) | Rhizomes | α-Glucosidase PTP1B | -7.6 | (Guo et al., 2015) |
| 169 | Licochalcone D | Glycyrrhiza uralensis (Leguminosae) | Rhizomes | α-Glucosidase PTP1B | -8 | (Guo et al., 2015) |
| 170 | Licoflavone B | Glycyrrhiza uralensis (Leguminosae) | Rhizomes | α-Glucosidase PTP1B | -8.7 | (Guo et al., 2015) |
| 171 | Licoflavone C | Glycyrrhiza uralensis (Leguminosae) | Rhizomes | α-Glucosidase PTP1B | -7.9 | (Guo et al., 2015) |
| 172 | Loganin | Cornus officinalis (Cornaceae) | Fruits | Rat soleus muscle BRIN-BD11 cells H4IIE cells | -7.2 | (Lin et al., 2011) |
| 173 | Lonchocarpene | Deguelia rufescens (Fabaceae) | Leaves | α-Glucosidase | -7.6 | (Pereira et al., 2012) |
| 174 | Lupeol acetate | Holarrhena curtisii (Apocynaceae) | Seedpods | α-Glucosidase | -8.7 | (Srisurichan & Pornpakakul, 2015) |
| 175 | Lupeol | Euonymus alatus (Celastraceae) | Corks | PTP1B α-Glucosidase | -8.3 | (S. Y. Jeong et al., 2015) |
| 176 | Luteolin 7-O-glucuronide | Salvia chloroleuca (Lamiaceae) | Aerial parts | α-Amylase α-Glucosidase | -8.6 | (B. Asghari et al., 2015) |
| 177 | Luteolin | Albizzia Lebbeck (Mimosaceae) | Barks | α-Glucosidase α-Amylase |  | (Ahmed et al., 2014) |
| 178 | Lysicamine | Tinospora crispa (Menispermaceae | Vines | α-Glucosidase α-Amylase | -7.3 | (Hamid et al., 2015) |
| 179 | Magnoflorine | Tinospora cordifolia (Menispermaceae) | Stems | α-Glucosidase | -7.3 | (Patel & Mishra, 2012) |
| 180 | Malabaricone A | Horsfieldia macrobotrys (Myristicaceae) | Fruits | α-Glucosidase | -6.6 | (Ramadhan & Phuwapraisirisan, 2015) |
| 181 | Maplexin C | Acer rubrum (Sapindaceae) | Stems | α-Glucosidase | -8.2 | (Wan et al., 2012) |
| 182 | Maplexin D | Acer rubrum (Sapindaceae) | Stems | α-Glucosidase | -8.9 | (Wan et al., 2012) |
| 183 | Maplexin E | Acer rubrum (Sapindaceae) | Stems | α-Glucosidase | -9 | (Wan et al., 2012) |
| 184 | Medicarpin | Dalbergia odorífera (Leguminosae) | Heartwood | α-Glucosidase | -7.1 | (Choi et al., 2010) |
| 185 | Meso-dihydroguaiaretic acid | Myristica fragrans (Myristicaceae) | Semen | PTP1B 32D cells | -6.2 | (Yang et al., 2006) |
| 186 | 5-Methoxypsoralen | Angelica keiskei (Apiaceae) | Stems | PTP1B | -7 | (J. L. Li et al., 2015) |
| 187 | Methyl 3,4-dicaffeoylquinate | Gynura divaricata (Asteraceae) | Aerial parts | α-Glucosidase PTP1B | -7.6 | (J. Chen et al., 2014) |
| 188 | Methyl gallate | Acer rubrum (Sapindaceae) | Stems | α-Glucosidase | -5.6 | (Wan et al., 2012) |
| 189 | Momordicoside F1 | Momordica charantia (Cucurbitaceae) | Fruits | α-Glucosidase | -8.6 | (Nhiem et al., 2010) |
| 190 | Momordicoside F2 | Momordica charantia (Cucurbitaceae) | Fruits | α-Glucosidase | -8.6 | (Nhiem et al., 2010) |
| 191 | Momordicoside I | Momordica charantia (Cucurbitaceae) | Fruits | α-Glucosidase | -8.5 | (Nhiem et al., 2010) |
| 192 | Moracin I | Morus alba var. tatarica (Moraceae) | Root barks | α-Glucosidase PTP1B | -7.7 | (Zhang et al., 2014) |
| 193 | Mucronulatol | Dalbergia odorífera (Leguminosae) | Heartwood | α-Glucosidase | -8 | (Choi et al., 2010) |
| 194 | Mulberrofuran A | Morus alba var. tatarica (Moraceae) | Root barks | α-Glucosidase PTP1B | -7.3 | (Zhang et al., 2014) |
| 195 | Munduleaflavanone A | Angelica keiskei (Apiaceae) | Aerial parts | α-Glucosidase | -7.4 | (Luo et al., 2012) |
| 196 | Myricetin | Cyclocarya paliurus (Cyclocaryaceae) | Barks | α-Glucosidase Glycogen phos phorilase | -7.8 | (Li et al., 2011) |
| 197 | Myrigalone-B | Syzygium aqueum (Myrtaceae) | Leaves | 3T3-L1 cells | -7 | (Manaharan et al., 2013) |
| 198 | Myrigalone-G | Syzygium aqueum (Myrtaceae) | Leaves | 3T3-L1 cells | -7.2 | (Manaharan et al., 2013) |
| 199 | Neokotalanol | Salacia chinensis (Hippocrateaceae) | Stems | α-Glucosidase | -6 | (Morikawa et al., 2015) |
| 200 | Neosalacinol | Salacia chinensis (Hippocrateaceae) | Stems | α-Glucosidase | -5.7 | (Morikawa et al., 2015) |
| 201 | Nigelladine A | Nigella glandulífera (Ranunculaceae) | Seeds | PTP1B | -7.5 | (Q. B. Chen et al., 2014) |
| 202 | Nigelladine B | Nigella glandulífera (Ranunculaceae) | Seeds | PTP1B | -7.3 | (Q. B. Chen et al., 2014) |
| 203 | Nordentatin | Clausena harmandiana (Rutaceae) | Roots | L6 cells | -7.4 | (Noipha et al., 2010) |
| 204 | N-trans-Feruloyltyramine | Tinospora crispa (Menispermaceae) | Vines | α-Glucosidase α-Amylase | -7.1 | (Hamid et al., 2015) |
| 205 | Nuciferine | Nelumbo nucifera (Nelumbonaceae) | Leaves | Mouse pancreas cells INS-1E -cells | -7.8 | (Nguyen et al., 2012) |
| 206 | Nummularine-R | Ziziphus oxyphylla (Rhamnaceae) | Leaves | α-Glucosidase | -9.8 | (Choudhary et al., 2011) |
| 207 | Nuzhenide | Fraxinus excelsior (Oleaceae) | Seeds | 3T3-L1 cells HEK-293 cells | -8.4 | (N. Bai et al., 2010) |
| 208 | Okanin | Andromachia igniaria (Asteraceae) | Aerial parts | α-Amylase α-Glucosidase | -7.5 | (Saltos et al., 2015) |
| 209 | Oleoside dimethyl ester | Fraxinus excelsior (Oleaceae) | Seeds | 3T3-L1 cells HEK-293 cells | -7.5 | (Naisheng Bai et al., 2010) |
| 210 | Oresbiusin A | Tradescantia spathacea  (Commelinaceae) | Aerial parts | PTB1B | -6 | (Vo et al., 2015) |
| 211 | Orientanol E | Erythrina addisoniae (Leguminosae) | Stem bark | PTP1B | -7.3 | (Bae et al., 2006) |
| 212 | Otobaphenol | Myristica fragrans (Myristicaceae) | Semen | PTP1B 32D cells | -8.3 | (Yang et al., 2006) |
| 213 | Oxypeucedanin hydrate | Ducrosia anethifolia (Apiaceae) | Stems Leaves | α-Glucosidase α-Amylase β-galactosidase | -7.8 | (Shalaby et al., 2014) |
| 214 | Paeonoside | Paeonia suffruticosa (Paeoniaceae) | Root barks | HepG2 cells | -7.3 | (Ha do et al., 2010) |
| 215 | Palbinone | Paeonia suffruticosa (Paeoniaceae) | Root barks | HepG2 cells | -8.2 | (Ha do et al., 2009) |
| 216 | Palmatine | Tinospora cordifolia (Menispermaceae) | Stems | α-Glucosidase | -7.6 | (Patel & Mishra, 2012) |
| 217 | Palmitic acid | Agrimonia pilosa (Rosaceae) | Aerial parts | PTP1B α-Glucosidase | -5.2 | (Braham Na et al., 2016) |
| 218 | p-Coumaric acid | Euonymus alatus (Celastraceae) | Corks | PTP1B α-Glucosidase | -6.3 | (Su‐Yang Jeong et al., 2015) |
| 219 | Pelargonidin-3-glucoside | Zea mays (Poaceae) | Kernels | α-Glucosidase | -7.8 | (Nile & Park, 2014) |
| 220 | Peltatoside | Psidium guajava (Myrtaceae) | Leaves | DPP-4 | -8.5 | (Eidenberger et al., 2013) |
| 221 | Pentadecanoic acid | Edgeworthia gardneri (Thymelaeaceae) | Flowers | HeLa cells | -5.2 | (Gao et al., 2015) |
| 222 | Petasin | Petasites japonicus (Asteraceae) | Shoots | H4IIE cells 3T3-L1 cells C2C12 cells | -7.1 | (Adachi et al., 2014) |
| 223 | Phloretin | Syzygium aqueum (Myrtaceae) | Leaves | 3T3-L1 cells | -7.3 | (Manaharan et al., 2013) |
| 224 | Phlorizin | Rhus javanica (Anacardiaceae) | Stem bark | α-Glucosidase | -7.8 | (Cho et al., 2013) |
| 225 | Pipernonaline | Piper longum (Piperaceae) | Fruits | Microsome rat liver | -6.9 | (Lee et al., 2006) |
| 226 | Piperrolein B | Piper nigum (Piperaceae) | Fruits | Microsome rat liver | -6.4 | (Lee et al., 2006) |
| 227 | Ponkoranol | Salacia chinensis (Hippocrateaceae) | Stems | α-Glucosidase | -6.6 | (Morikawa et al., 2015) |
| 228 | Procyanidins B3 | Rosa rugosa (Rosaceae) | Roots | Rat intestinal sucrase inhibition | -8.5 | (Thao, Luyen, Tai, et al., 2014) |
| 229 | Psoralen | Ducrosia anethifolia (Apiaceae) | Stems Leaves | α-Glucosidase α-Amylase β-galactosidase | -7 | (Shalaby et al., 2014) |
| 230 | Psoralidin | Psoralea corylifolia (Leguminosae) | Seeds | PTP1B | -8.5 | (Kim et al., 2005) |
| 231 | Pycnalin | Acer pycnanthum (Aceraceae) | Leaves | α-Glucosidase | -7.3 | (Ogawa et al., 2011) |
| 232 | Quinoline | Ruta chalepensis (Rutaceae) | Leaves | α-Glucosidase α-Amylase | -5.4 | (Park & Lee, 2015) |
| 233 | Quinovic acid | Fagonia cretica (Zygophyllaceae) | Aerial parts | DPP-4 | -8.5 | (Saleem et al., 2014) |
| 234 | Ratanhiaphenol III | Krameria lappacea (Krameriaceae) | Roots | PTP1B C2C12 cells | -7.8 | (Heiss et al., 2012) |
| 235 | Rhoifolin | Citrus grandis (Rutaceae) | Leaves | 3T3-L1 cells | -8.9 | (Rao et al., 2011) |
| 236 | Robustaflavone | Selaginella tamariscina (Selaginellaceae) | Aerial parts | PTP1B | -9.6 | (P.-H. Nguyen et al., 2015) |
| 237 | Rosamultic acid | Potentilla fulgens (Rosaceae) | Roots | α-Glucosidase | -8.2 | (D. Kumar, R. Ghosh, et al., 2013) |
| 238 | Rosamultin | Rosa rugosa (Rosaceae) | Roots | Rat intestinal sucrase inhibition | -9.5 | (Thao, Luyen, Jo, et al., 2014) |
| 239 | Salacinol | Salacia chinensis (Hippocrateaceae) | Stems | α-Glucosidase | -6.6 | (Morikawa et al., 2015) |
| 240 | Scirpusin A | Syagrus romanzoffiana (Arecaceae) | Seeds | α-Glucosidase | -8.4 | (Lam et al., 2008) |
| 241 | Scirpusin B | Cyperus rotundus (Cyperaceae) | Rhizomes | α-Glucosidase α-Amylase | -8.4 | (Tran et al., 2014) |
| 242 | Scopoletin | Artemisia capillaris (Asteraceae) | Whole plant | α-Glucosidase PTP1B | -6.3 | (Islam et al., 2013b) |
| 243 | Scutellarein | Scoparia dulcis (Scrophulariaceae) | Aerial parts | α-Glucosidase | -8.1 | (Liu et al., 2014) |
| 244 | Amentoflavon  e | Selaginella tamariscina (Selaginellaceae) | Aerial parts | PTP1B | -8 | (P. H. Nguyen, D. J. Ji, et al., 2015) |
| 245 | Selariscinins E | Selaginella tamariscina (Selaginellaceae) | Aerial parts | PTP1B | -9.8 | (P.-H. Nguyen et al., 2015) |
| 246 | Sennoside A | *Rhei Rhizoma* (Polygonaceae) | Rhizome Roots | α-Glucoamylase | -8.6 | (Choi et al., 2006) |
| 247 | Sigmoidin E | Erythrina mildbraedii (Leguminosae) | Root bark | PTP1B | -9.3 | (Na et al., 2006) |
| 248 | Sigmoidin K | Erythrina abyssinica (Fabaceae) | Stem bark | C2C12 cells | -8 | (Nguyen et al., 2010) |
| 249 | Smeathxanthone A | Garcinia mangostan  (Clusiaceae) | Seedcases | α-Glucosidase | -8.8 | (Ryu et al., 2011) |
| 250 | Specioside | Kigelia pinnata (Bignoniaceae) | Twigs | GLUT4 | -8.8 | (Khan et al., 2012) |
| 251 | Syringaldehyde | Pandanus tectorius (Pandanaceae) | Fruits | α-Glucosidase | -5.5 | (Mai et al., 2015) |
| 252 | Syringaresinol | Cucurbita moschata (Cucurbitaceae) | Stems | Insulin sensitizer | -8 | (Chang et al., 2014) |
| 253 | Tachioside | Piper retrofractum (Piperaceae) | Leaves | α-Glucosidase | -7.2 | (Luyen et al., 2014) |
| 254 | Tanshinone I | Salvia miltiorrhiza (Lamiaceae) | Roots | GLUT4  MAPK & PI3K | -8.9 | (Jung et al., 2009) |
| 255 | Tanshinone IIA | Salvia miltiorrhiza (Lamiaceae) | Roots | GLUT4  MAPK & PI3K | -9 | (Jung et al., 2009) |
| 256 | Taxoquinone | Metasequoia glyptostroboides (Cupressaceae) | Cones | α-Glucosidase | -7.8 | (Bajpai et al., 2015) |
| 257 | Tectorigenin | Dalbergia odorífera  (Leguminosae) | Heartwood | α-Glucosidase | -7.5 | (Choi et al., 2010) |
| 258 | Tetrahydroauroglaucin | Phlomis tuberosa (Lamiaceae) | Roots | α-Glucosidase | -6.7 | (Yang et al., 2015) |
| 259 | Tormentic acid | Agrimonia pilosa (Rosaceae) | Aerial parts | PTP1B α-Glucosidase | -9 | (B. Na et al., 2016) |
| 260 | Tricin | Echinochloa utilis (Poaceae) | Seeds | α-Glucosidase | -7.7 | (Seo et al., 2015b) |
| 261 | Tuberosin | Pueraria thunbergiana (Leguminosae) | Roots | α-Glucosidase  α-Amylase | -8.3 | (Park et al., 2009) |
| 262 | Umbelliferone | Edgeworthia gardneri (Thymelaeaceae) | Flowers | PPARγ  PPARβ | -6.5 | (Gao et al., 2015) |
| 263 | Vanillic acid | Cyclocarya paliurus (Cyclocaryaceae) | Barks | α-Glucosidase Glycogen phosphorilase | -5.6 | (Li et al., 2011) |
| 264 | Vanillin | Pandanus tectorius (Pandanaceae) | Fruits | α-Glucosidase | -5 | (Mai et al., 2015) |
| 265 | Vasicine | Adhatoda vasica (Acanthaceae) | Leaves | α-Glucosidase | -6.4 | (H. Gao et al., 2008) |
| 266 | Vasicinol | Adhatoda vasica (Acanthaceae) | Leaves | α-Glucosidase | -6.3 | (H. Gao et al., 2008) |
| 267 | Verminoside | Kigelia pinnata (Bignoniaceae) | Twigs | GLUT4 | -8.2 | (Khan et al., 2012) |
| 268 | Vindolidine | Catharanthus roseus (Apocynaceae) | Leaves | PTP1B | -7.1 | (Tiong et al., 2013) |
| 269 | Vindoline | Catharanthus roseus (Apocynaceae) | Leaves | PTP1B | -7.3 | (Tiong et al., 2013) |
| 270 | Vindolinine | Catharanthus roseus (Apocynaceae) | Leaves | PTP1B | -7.5 | (Tiong et al., 2013) |
| 271 | Xanthoangelol | Angelica keiskei (Apiaceae) | Stems | GLUT4 | -7.3 | (Ohta et al., 2015) |
| 272 | 4-hydroxyisoleucine | Trigonella foenumgraecum (Fabaceae) | Seeds | GLUT4 | -4.8 | (Jaiswal et al., 2012) |
| 273 | 25R antcin K | *Antrodia cinnamomea*  *(*Fomitopsidaceae) | Fruiting bodies | α-Glucosidase | -8.8 | (Huang et al., 2018) |
| 274 | 25S antcin K | *Antrodia cinnamomea*  *(*Fomitopsidaceae) | Fruiting bodies | α-Glucosidase | -8.7 | (Huang et al., 2018) |
| 275 | 2,4,6-tribromophenol | *Grateloupia elliptica (*Halymeniaceae) | - | α-Glucosidase | -4.7 | (Kim et al., 2008) |
| 276 | 2,4-dibromophenol | *Grateloupia elliptica*  *(*Halymeniaceae) | - | α-Glucosidase | -4.6 | (Kim et al., 2008) |
| 277 | andrographolide | Andrographis paniculata  *(Acanthaceae)* | Leaves | GLUT4 | -7.8 | (Yu et al., 2003) |
| 278 | callyspongynic acid | *Callyspongia truncate* (Callyspongiidae) | - | α-glucosidase | -5.5 | (Nakao et al., 2002) |
| 279 | 3,4-dibromo-5-(2-bromo-3,4-  dihydroxy-6-(ethoxymethyl)benzyl)  benzene-1,2-diol | *Rhodomela confervoides*  (Rhodomelaceae) | - | PTP1B | -6.5 | (Shi et al., 2013) |
| 280 | cowanol | *Garcinia cowa*  *(Clusiaceae)* | Leaves | α-Glucosidase | -8.4 | (Phukhatmuen et al., 2020) |
| 281 | dehydroeburicoic acid | *Antrodia cinnamomea*  (Fomitopsidaceae) | Fruiting bodies | α-Glucosidase | -8.6 | (Huang et al., 2018) |
| 282 | dysidine | *Dysidea villosa* (Dysideidae) | - | PTP1B  GLUT4 | -8.5 | (Y. Zhang et al., 2009) |
| 283 | eburicoic acid | *Antrodia cinnamomea*  (Fomitopsidaceae) | Fruiting bodies | α-Glucosidase | -7.8 | (Huang et al., 2018) |
| 284 | stachysetin | *Stachys iva*  *(Lamiaceae)* | Aerial parts | *In silico* | -10.4 | (Pritsas et al., 2020) |
| 285 | sativanone | *Dalbergia tonkinensis*  (Fomitopsidaceae) | Heartwood | α-Glucosidase | -7.2 | (Nguyen et al., 2018) |
| 286 | palinurin | *Ircinia dendroides* (Irciniidae) | - | GSK-3β | -6.7 | (Bidon-Chanal et al., 2013) |
| 287 | Oenothein C | Eugenia jambolana (Myrtaceae) | Seeds | α-Glucosidase | -9.2 | (Omar et al., 2012) |
| 288 | Vaticanol A | Cotylelobium melanoxylon (Dipterocarpaceae) | Wood, Bark | α-Glucosidase, aldose reductase | -8.3 | (Matsuda et al., 2009) |
| 289 | Sennoside B | Rhizoma Rhei (Polygonaceae) | Rhizome, Roots | α-Glucoamylase, insulin stimulation | -8.4 | (Choi et al., 2006) |
| 290 | Thonningianin A | Penthorum chinense (Saxifragaceae) | Aerial parts | α-Amylase | -11.3 | (Huang et al., 2015) |
| 291 | Stachyurin | Lagerstroemia speciosa (Lythraceae) | Leaves | PPAR-γ | -10.4 | (Bai et al., 2008) |
| 292 | Octadecyl caffeate | Coptis chinensis (Ranunculaceae) | Roots | Glucose uptake | -5.7 | (Yang et al., 2014) |
| 293 | Vindolicine | Catharanthus roseus  (Apocynaceae) | Leaves | PTP1B | -8.2 | (Tiong et al., 2013) |
| 294 | Vaticanol E | Cotylelobium melanoxylon (Dipterocarpaceae) | Wood, Bark | α-Glucosidase,  aldose reductase | -8.7 | (Matsuda et al., 2009) |
| 295 | taxifolin | *Coreopsis tinctoria*  *(Asteraceae)* | Capitula | PTP1B | -7.9 | (Begmatov et al., 2020) |
| 296 | 3,6,7,4’,5’-Pentamethoxy-5,3’-dihydroxyflavone | Caesalpinia pulcherrima (Fabaceae) | Aerial parts | Aldose reductase | -7.3 | (Kumar et al., 2015) |
| 297 | gutiferone I | *Garcinia cowa*  *(Clusiaceae)* | Leaves | α-Glucosidase | -8.2 | (Phukhatmuen et al., 2020) |
| 298 | Lunariifolioside | Phlomis stewartii (Lamiaceae) | Whole plant | α-Glucosidase | -8.3 | (Jabeen et al., 2013) |
| 299 | Momordicine II | Momordica charantia (Cucurbitaceae) | Fruits and Seeds | ↑ pancreatic β-cells secretion | -9.3 | (Keller et al., 2011) |
| 300 | Momordicoside G | Momordica charantia  (Cucurbitaceae) | Fruits | α-Glucosidase | -9.1 | (Nhiem et al., 2010) |
| 301 | Momordicoside M | Momordica charantia  (Cucurbitaceae) | Fruits | α-Glucosidase | -8.8 | (Nhiem et al., 2010) |
| 302 | Momordicoside B | Momordica charantia  (Cucurbitaceae) | Fruits | ↑ GLUT4,  ↑ AMPK | -8.6 | (Tan et al., 2008) |
| 303 | Momordicoside A | Momordica charantia  (Cucurbitaceae) | Fruits | α-Glucosidase | -8.3 | (Nhiem et al., 2010) |
| 304 | Gartanin | Garcinia mangostan  (Clusiaceae) | Seedcases | α-Glucosidase | -7.9 | (Ryu et al., 2011) |
| 305 | α-galactosylceramide | *Agelas mauritianus*  (Agelasidae) | - | Natural killer cells (NKT) | -5.7 | (Barde et al., 2015) |
| 306 | catharanthine | *Catharanthus roseus*  *(*Apocynaceae) | Stems,  Leaves | - | -7.3 | (B Gaikwad et al., 2014) |
| 307 | Harmane | *Tribulus terrestris*  *(Zygophyllaceae)* | - | - | -6.7 | (B Gaikwad et al., 2014) |
| 308 | hesperidin | *Citrus fruits* | Fruits | pancreatic β-cell | -9.6 | (Hanchang et al., 2019) |
| 309 | Lactucain A | *Lactuca indica*  *(Asteraceae)* | Whole plant | - | -10.7 | (Hou et al., 2003) |
| 310 | lactucaside | *Lactuca indica*  *(Asteraceae)* | Whole plant | - | -8.5 | (Hou et al., 2003) |
| 311 | Leucodelphinidin | *Ficus bengalensis*  *(Moraceae)* | Bark | - | -7.5 | (Geetha et al., 1994) |
| 312 | naringin | *Cochlospemum vitifolium*  *(Bixaceae)* | Fruits | α-glucosidase | -8.6 | (Vinayagam et al., 2015) |
| 313 | 24-methyl-lophenol | *Aloe vera (Aloe barbadensis MILLER)*  *(*[*Asphodelaceae*](https://www.google.com/search?rlz=1C1CHBF_enBD842BD842&sxsrf=AOaemvKT5bxRYM7Rv_otOxQf1tlDR0jXgw:1630563613651&q=Asphodelaceae&stick=H4sIAAAAAAAAAONgVuLUz9U3MCovT6p4xGjCLfDyxz1hKe1Ja05eY1Tl4grOyC93zSvJLKkUEudig7J4pbi5ELp4FrHyOhYXZOSnpOYkJqcmpgIA901wh1QAAAA)*)* | *Aloe vera* gel | HbA1c | -8.4 | (Tanaka et al., 2006) |
| 314 | 4-hydroxybenzoic acid | *Pandanus odorus (Pandanaceae)* | Roots | - | -5.3 | (Peungvicha et al., 1998) |
| 315 | coumarin | *Hemionitis arifolia (Pteridaceae), Clausena anisate (Rutaceae)* | Whole plants, roots | pancreatic β-cells stimulator | -6.1 | (Ajikumaran Nair et al., 2006) |
| 316 | Phloroglucinol | *Ecklonia stolonifera (Lessoniaceae)* | - | α-Glucosidase,  PTP1B | -5.3 | (Lee & Jeon, 2013) |
| 317 | Eugenol | *Ocimum gratissimum (Lamiaceae)* | Leaves | α-Glucosidase, | -5.6 | (Singh et al., 2016) |
| 318 | Berberine | *Berberis lyceum (Berberidaceae)* | Roots | GLUT4  AMPK | -8.1 | (Gulfraz et al., 2008) |
| 319 | Bergapten | *Ficus exasperata (Moraceae)* | Root bark | - | -7.1 | (Famobuwa et al., 2019) |
| 320 | Castanospermine | *Xanthocercis*  *Zambesiaca (Fabaceae)* | Leaves | - | -5.6 | (Nojima et al., 1998) |
| 321 | Ursolic acid | *Leandra lacunosa*  *(Melastomataceae)* | Aerial  parts | - | -8.9 | (Cunha et al., 2008) |
| 322 | Silybin | *Silybum marianum (Asteraceae)* | Seeds | Regulate pancreatic β-cell function | -8.4 | (Huseini et al., 2006) |
| 323 | Diallyl disulfide-oxide | *Allium sativum (Alliaceae)* | Bulbs | ↑ Insulin secretion | -3.7 | (Chauhan et al., 2010) |
| 324 | Epigallocatechin 3-gallate | *Camellia sinensis (Theaceae)* | - | Modulation of the redox state of the cell,  insulin-mimetic action | -8.1 | (Waltner-Law et al., 2002) |
| 325 | Secoisolariciresinol | *Taxus yunnanensis (Taxaceae)* | Wood | - | -6.3 | (Banskota et al., 2006) |
| 326 | Fagomine | *Xanthocercis*  *Zambesiaca*  *(Fabaceae)* | Leaves | - | -4.8 | (Nojima et al., 1998) |
| 327 | Catechin | *Cassia fistula (Fabaceae)* | Stems | GLUT4,  pancreatic β-cell | -7.2 | (Daisy et al., 2010) |
| 328 | Diosgenin | *Dioscorea bulbifera (Dioscoreaceae)* | Bulbs | α-Glucosidase,  α-Amylase | -9.4 | (Ghosh et al., 2014) |
| 329 | S- methyl cysteine sulfoxide | *Allium cepa (Alliaceae)* | Bulbs | ↑ Insulin secretion | -4.4 | (Kumari & Augusti, 2002) |
| 330 | Eremanthin | *Costus speciosus (Costaceae)* | Rhizomes | Stimulating insulin release from β-cell | -8 | (Eliza et al., 2009a) |
| 331 | Nimbidin | *Azadirachta indica (Meliaceae)* | Leaf,  Seed | - | -7.8 | (Makheswari & Sudarsanam, 2012) |
| 332 | Isoorientin | *Gentiana olivieri*  *(Gentianaceae)* | Aerial  parts | - | -8 | (Sezik et al., 2005) |
| 333 | Phlorofucofuroeckol-A | *Ecklonia cava*  *(Lessoniaceae)* | - | α-Glucosidase,  α-Amylase | -8.8 | (S. H. Lee et al., 2009) |
| 334 | 6,6′-Bieckol | *Ecklonia cava*  *(Lessoniaceae)* | - | α-Glucosidase,  α-Amylase | -9.5 | (S. H. Lee et al., 2009) |
| 335 | Eckol | *Ecklonia maxima*  *(Lessoniaceae)* | - | α-Glucosidase | -8.4 | (Gunathilaka et al., 2020) |
| 336 | Bassic acid | *Bumelia sartorum (Sapotaceae)* | Root bark | ↑Insulin secretion | -8.2 | (Naik et al., 1991) |
| 337 | 3-O-methyl-D-chiro-inositol | *Glycine max*  *(Fabaceae)* | Seed | Activating glucose metabolism | -5.4 | (Kang et al., 2006) |
| 338 | Garcinol | *Garcinia quaesita (Clusiaceae)* | Fruits | Pancreatic β cells | -8.2 | (Liyanagamage et al., 2020) |
| 339 | Limonene | *Aegle marmelos (Rutaceae)* | Leaves | - | -9.5 | (Vieira et al., 2018) |
| 340 | Lanosterol | *Garcinia prainiana (Clusiaceae)* | Twigs | ↑Glucose uptake,  Insulin mimetic effect | -8.3 | (Susanti et al., 2013) |
| 341 | Pheophorbide-A | *Gelidium amansii (Gelidiaceae)* | - | α-Glucosidase,  α-Amylase | -8.3 | (Kim et al., 2019) |
| 342 | Ginsenosides | *Panax ginseng (Araliaceae)* | Roots | AMPK signaling pathway | -8.1 | (Lee et al., 2010) |
| 343 | Malvidin-3-O-Glucoside | *Phaseolus vulgaris*  *(Fabaceae)* | Beans | α-Glucosidase,  α-Amylase | -7.4 | (Mojica et al., 2017) |
| 344 | Naringenin | *Ajuga iva*  *(Lamiaceae)* | Whole plants | ↑Insulin secretion | -8.2 | (Taleb-Senouci et al., 2009) |
| 345 | β-glucan | *Eleusine coracana (Poaceae)* | Seeds | α-Glucosidase,  α-Amylase | -7.1 | (Divya et al., 2020) |
| 346 | Galactomannan | *Trigonella foenum-graecum (Fabaceae)* | Seeds | ↑Insulin level  ↑Tissue sensitivity of insulin action | -8.9 | (Puri et al., 2002) |
| 347 | Saponarin | *Tinospora cordifolia*  *(Menispermaceae)* | Leaves | α-Glucosidase | -8.2 | (Sengupta et al., 2009) |
| 348 | Silychristin | *Silybum marianum*  *(Asteraceae)* | Fruits | α-Amylase | -8.8 | (Kato et al., 2020) |
| 349 | Luteolin-6,8-di-C-glucoside | *Montanoa*  *Bipinnatifida, Passiflora*  *Bahiensis* | - | - | -7.5 | (Figueirinha et al., 2008) |
| 350 | Vicenin-2 | *Ocimum gratissimum,*  *Ocimum basilicum*  *(Lamiaceae)* | Leaves | GLUT2,  ↑Insulin Secretion | -7.5 | (Casanova et al., 2017) |
| 351 | Gingerol | *Zingiber officinale (Zingiberaceae)* | Bulb | - | -5.9 | (Makheswari & Sudarsanam, 2012) |
| 352 | Petunidin 3-glucoside | *Vitis vinifera (Vitaceae)* | Fruits | - | -7.4 | (Morimitsu et al., 2002) |
| 353 | peonidin 3-glucoside | *Vitis vinifera*  *(Vitaceae)* | Fruits | - | -7.7 | (Morimitsu et al., 2002) |
| 354 | Resveratrol | *Common in grapes and berries* | Fruits | AMPK  GLUT4 | -6.9 | (Do et al., 2012) |
| 355 | Pongamol | *Pongamia pinnata*  *(Fabaceae)* | Fruits | PTP1B | -6.8 | (Tamrakar et al., 2008) |
| 356 | curcumin | *Curcuma longa*  *(*Zingiberaceae) | Rhizome | - | -7.4 | (Kuhad & Chopra, 2007) |
| 357 | Ginsenoside RG1 | *Panax notoginseng (Araliaceae)* | Roots | - | -7.5 | (Yu et al., 2015) |
| 358 | Silydianin | *Silybum marianum*  *(Asteraceae)* | Seeds | Regulate pancreatic β-cell function | -8.2 | (Huseini et al., 2006) |
| 359 | dieckol | *Ecklonia bicyclis (Lessoniaceae)* | - | α-glucosidase | -10.8 | (Lee et al., 2012a) |
| 360 | 3-Caffeoylquinic acid | *Ilex kudingcha*  *(Aquifoliaceae)* | Leaves | α-glucosidase | -7.5 | (Xu et al., 2015) |
| 361 | Glyceollin I | *Glycine max (Fabaceae)* | Soybean | ↑Insulin secretion | -8.9 | (Park et al., 2010) |
| 362 | Eupatilin | *Artemisia ludoviciana (Asteraceae)* | Aerial  Parts | Modulate  intracellular  Ca^2+^ | -7.7 | (Anaya-Eugenio et al., 2014) |
| 363 | Quercetin | *Euonymus alatus (Celastraceae)* | Leaves | PPARγ | -7.9 | (Fang et al., 2008) |
| 364 | Beta carotene | *Cucumis*  *Metuliferus (Cucurbitaceae)* | Fruits | - | -7.8 | (Makheswari & Sudarsanam, 2012) |
| 365 | Kaempferol | *Euonymus alatus (Celastraceae)* | Leaves | PPARγ | -7.8 | (Fang et al., 2008) |
| 366 | Genistein | *Glycine max*  *(Fabaceae)* | *Beans* | Glucose-6-phosphatase | -8.3 | (Lee, 2006) |
| 367 | Apigenin | *Newbouldia laevis (Bigoniaaceae)* | Leaves | Glucose-6 phosphatase, Fructose-1,6-biphosphatase | -8.1 | (Osigwe et al., 2017) |
| 368 | Fucoxanthin | *Eisenia bicyclis (Lessoniaceae)* | - | PTP1B,  Aldose reductase | -8.6 | (Peng et al., 2011) |
| 369 | Fucosterol | *Ecklonia stolonifera (Laminariacea)* | - | PTP1B,  Aldose reductase | -8 | (Jung et al., 2008) |
| 370 | Mangiferin | *Anemarrhena asphodeloides (Asparagaceae)* | Rhizome | Reduce insulin resistance | -7.6 | (Miura et al., 2001) |
| 371 | Aloeresin A | *Aloe vera* | - | Suppress  α-glucosidase activity, ↓insulin resistance) | -8.8 | (Jong-Anurakkun et al., 2008) |
| 372 | enhydrin | *Smallanthus sonchifolius (Asteraceae)* | Leaves | - | -7.2 | (Genta et al., 2010) |
| 373 | 3,5-O-dicaffeoylquinic acid | *Ilex paraguariensis (Aquifoliaceae)* | Leaves | ↑Insulin sensitivity | -9 | (Hussein et al., 2011) |
| 374 | 24- methylene cycloartanol | *Aloe barbadensis (Asphodelaceae)* | *Aloe vera* gel | HbA1c | -7.9 | (Tanaka et al., 2006) |
| 375 | Stigmasterol-3-O-β-  D-glucopyranoside | *Burkea africana (Fabacea)* | Roots | *α-*Amylase | -8.8 | (Feunaing et al., 2021) |
| 376 | S-allylmercapto-L-cysteine | *Allium cepa (Alliaceae)* | Bulb | - | -4.7 | (Eidi et al., 2006) |
| 377 | isotaxiresinol | *Taxus yunnanensis*  *(Taxaceae)* | Wood | - | -7.3 | (Banskota et al., 2006) |
| 378 | Ginsenoside rb1 | *Panax ginseng*  *(*Araliaceae) | - | GLUT4  AMPK | -8 | (Zhou et al., 2019) |
| 379 | Taxiresinol | *Taxus yunnanensis*  *(Taxaceae)* | Wood | - | -7.8 | (Banskota et al., 2006) |
| 380 | Sargahydroquinoic acid | *Sargassum serratifolium (Sargassaceae)* | - | PTP1B | -7.5 | (Ezzat et al., 2018) |
| 381 | dysidine | *Dysidea villosa (Dysideidae)* | - | PTP1B | -8.8 | (Li et al., 2009) |
| 382 | kinsenoside | *Anoectochilus roxburghii (Orchidaceae)* | Whole plant | Repairing pancreatic β cells | -6.5 | (Y. Zhang et al., 2007) |
| 383 | Dioxinodehydroeckol | *Eisenia bicyclis (Lessoniaceae)* | - | α-glucosidase | -8.4 | (Abdelsalam et al., 2019) |
| 384 | 7-phloroeckol | *Eisenia bicyclis (Lessoniaceae)* | - | PTP1B,  α-glucosidase | -9.7 | (Abdelsalam et al., 2019) |
| 385 | Aspalathin | *Aspalathus linearis (Fabaceae)* | Leaves and stem | Improve β -cell function,  PI3K | -7.1 | (Kawano et al., 2009) |
| 386 | gymnemic acid | *Gymnema sylvestre (Apocynaceae)* | Leaves | regeneration of β-cells | -8.6 | (Ahmed et al., 2010) |
| 386 | cycloartanol | *Aloe barbadensis MILLER (Asphodelaceae)* | *Aloe vera* gel | HbA1c | -7.6 | (Tanaka et al., 2006) |
| 387 | Momordicin | *Momordica charantia (Cucurbitaceae)* | Fruit | insulin-mimetic properties | -8 | (Singh et al., 2011) |
| 388 | Conophylline | *Ervatamia microphylla (Apocynaceae)* | Leaves | induce beta-cell differentiation | -9.8 | (Umezawa et al., 2018) |
| 389 | Aegeline | *Aegle marmelos (Rutaceaea)* | Leaves | - | -7.3 | (Narender et al., 2007) |
| 390 | Diphlorethohydroxycarmalol | *Ishige*  *Okamurae (Ishigeasceae)* | - | α -glucosidase  α-amylase | -8.8 | (Heo et al., 2009) |
| 391 | Amorfrutin 1 | *Glycyrrhiza foetida* (Fabaceae) | Roots | PPARγ | -8.2 | (Weidner et al., 2012) |
| 392 | Amorfrutin 2 | *Glycyrrhiza foetida* (Fabaceae) | Roots | PPARγ | -6.6 | (Weidner et al., 2012) |
| 393 | Amorfrutin 3 | *Glycyrrhiza foetida* (Fabaceae) | Roots | PPARγ | -8.2 | (Weidner et al., 2012) |
| 394 | Arenamides B | *Salinispora arenicola* |  | Nitric oxide and  PGE2 inhibition | -8.5 | (Asolkar et al., 2009) |
| 395 | Arenamides A | *Salinispora arenicola* |  | Nitric oxide and  PGE2 inhibition | -8.3 | (Asolkar et al., 2009) |
| 396 | Lutonarin | *Plagiomnium*  *cuspidatum* |  |  | -8 | (Anhut et al., 1992) |
| 397 | Isoscoparin-7-O-glucoside | *Plagiomnium*  *cuspidatum* |  |  | -8 | (Anhut et al., 1992) |
| 398 | Isorhamnetin 3,7-diglucoside | *Brassica juncea (Brassicaceae)* | mustard  Leaves | ↓ Glucose | -8.2 | (Yokozawa et al., 2002) |
| 399 | Palasonin | *Butea monosperma (Fabaceae)* | Fruit,  Leaves,  bark | ↓ Glucose | -6.2 | (Harish et al., 2014) |
| 400 | Fucodiphloroethol G | *Ecklonia cava (lessoniaceae)* | - | α-amylase | -7.8 | (Lee et al., 2012b) |
| 401 | 4-O-beta-Dglucopyranosylfagomine | *Xanthocercis*  *Zambesiaca (Fabaceae)* | Leaves | ↓ Glucose  ↑ Insulin | -6.5 | (Nojima et al., 1998) |
| 402 | Matesaponin 2 | *Ilex paraguariensis (aquifoliaceae_)* | Leaves | Augmentation of  GLP-1 production | -9.1 | (Hussein et al., 2011) |
| 403 | Trigonenoside Xb | *Trigonella foenumgraecum (Fabaceae)* | Seeds | ↓ Glucose | -8.8 | (Puri et al., 2002) |
| 404 | Sargaquinoic acid | *Sargassum serratifolium (Sargassaceae)* | - | 3T3-L1 cells  PTP 1B inhibitory activity  PPARα/γ activation | -7.3 | (Ali et al., 2017) |
| 405 | Stachysetin | *Stachys iva Griseb.* | - | In silico screening against 17  proteins implicated in diabetes, as also ligand-based similarity metrics against established anti-diabetic  drugs | -11.3 | (Pritsas et al., 2020) |
| 406 | 1-Caffeoyl-5-feruloylquinic acid | *Ilex paraguariensis (aquifoliaceae)* | Leaves | Regulate two or more  pathways (augmentation of  GLP-1 production) | -8 | (Hussein et al., 2011) |
| 407 | Gugulsterone | *Commiphora mukul (Burseraceae)* | Barks | improved PPARᵧ  expression,  inhibition 3T3-L1 preadipocytes | -8.5 | (Bhavna Sharma et al., 2009) |
| 408 | Pheophytin-A | *Saccharina japonica (Laminariaceae)* | - | Aldose reductase  inhibition | -8 | (Jung et al., 2013) |
| 409 | charantin | *Momordica*  *charantia (Cucurbitaceae)* | Fruits | α-glucosidase  α-amylase | -4.6 | (Ahamad, 2019) |
| 410 | Comatin | *Coprinus comatus broth* | Whole plant | Maintain glucose, improve glucose  tolerance | -4.8 | (Ding et al., 2010) |
| 411 | 5β, 19-epoxy-  3β,25-dihydroxycucurbita-6,23(E)-diene | *Momordica charantia (Cucurbitaceae)* | Dried gourds | ↓ Glucose,  Upregulated the expression of GLUT4 & AMP‑activated protein kinase α1 | -8.7 | (Jiang et al., 2016) |
| 412 | 24 Ethyl lophenol | *Aloe barbadensis MILLER (Asphodelaceae)* | Gel | ↓HbA1c levels | -8.2 | (Tanaka et al., 2006) |
| 413 | Trigonenoside XIIa/b | *Trigonella foenumgraecum (Fabaceae)* | Seeds | ↓ Glucose | -9.3 | (Puri et al., 2002) |
| 414 | Lophenol | *Aloe barbadensis MILLER (Asphodelaceae)* | Gel | ↓HbA1c levels | -8.1 | (Tanaka et al., 2006) |
| 415 | 3,7,25-Trihydroxycucurbita-5,23-dien-19-al | *Momordica charantia*  *(Cucurbitaceae)* | Dried gourds | ↓ Glucose  Insulin secretion | -8 | (Keller et al., 2011) |
| 416 | alpha-Amyrin acetate | *Streblus asper*  *(Moraceae)* | Bark | ↓ HbA1c | -8.5 | (Karan et al., 2013) |
| 417 | Apigenin fucopyranoside | *Averrhoa carambola (Oxalidaceae)* | Leaves | ↓ Glucose uptake (PI3-K -  PKC and  MAPK pathways) | -8.7 | (Cazarolli et al., 2012) |
| 418 | beta-Amyrin palmitate | *Hemidesmus*  *indicus (Asclepiadaceae)* | Roots | ↓ Glucose  ↑ Glycogen | -7.4 | (Nair et al., 2014) |
| 419 | Bruceine D | *Brucea javanica*  *(Simaroubaceae)* | Seeds | ↓ Glucose  increased insulin | -7.8 | (Ablat et al., 2017) |
| 420 | Chikusetsu saponin Iva | *Aralia taibaiensis*  *(Araliaceae)* | Root Barks | ↓ Glucose  insulin secretion from βTC3 cells  AMPK activator  enhancing membrane translocation of GLUT4 | -9.8 | (Cui et al., 2015) |
| 421 | Delphinidin 3-sambubioside-5-glucoside | *Aristotelia chilensis (Elaeocarpaceae)* | Fruits | ↑ Glucose uptake | -8.3 | (Rojo et al., 2012) |
| 422 | Gamma-Sitosterol | *Lippia nodiflora*  *(Verbenaceae)* | Whole plant | ↓ Glucose  ↑ Insulin | -5.5 | (Balamurugan et al., 2011) |
| 423 | Malonyl ginsenosides | *Panax ginseng*  *(Araliaceae)* | Roots | ↑ Insulin | -9.3 | (Liu et al., 2013) |
| 424 | Vescalagin | *Syzygium samarangense (Myrtaceae)* | Fruits | ↓ Glucose  ↓ Insulin | -9.5 | (Huang et al., 2016) |
| 425 | 2',4'-Dihydroxy-3',5'- dimethyl-6'- methoxychalcone | *Syzygium samarangense (Myrtaceae)* | Leaves | ↓ Glucose  Promoted Glucose Uptake  3T3-L1 Cells | -7.4 | (Hu et al., 2014) |
| 426 | 2R,3R taxifolin 3-Orhamnoside | *Hydnocarpus*  *alpina (Flacourtiaceae)* | Leaves | ↑ Insulin  ↑ Insulin  tolerance  ↓ HbA1c | -7.8 | (Balamurugan et al., 2015) |
| 427 | 3-O-Acetyloleanolic acid | *Eysenhardtia*  *platycarpa*  *(Leguminosae)* | Leaves, Branches, Bark | ↓ Glucose | -9.3 | (Narvaez-Mastache et al., 2006) |
| 428 | 4-Hydroxypipecolic acid | *Peganum harmala*  *(Zygophyllaceae)* | Seeds | ↓ Glucose  ↓ Insulin  ↓ Glucagon  increased GLUT 4 translocation | -5.1 | (Naresh et al., 2012) |
| 429 | Acacetin | *Anoda cristata*  *(Malvaceae)* | Aerial parts | ↓ Glucose  Activation of CaMKII-AMPK pathway  Enhancement of GLUT4 translocation | -8.1 | (Kwon et al., 2020) |
| 430 | Aloe-emodin | *Rheum emodi*  *(Polygonaceae)* | Rhizomes | ↓ Glucose  α-glucosidase | -8 | (Arvindekar et al., 2015) |
| 431 | Arglanin | *Artemisia ludoviciana (Asteraceae)* | Aerial parts | Block K+-  ATP channels | -7.3 | (Anaya-Eugenio et al., 2014) |
| 432 | Azorellanol | *Azorella compacta*  *(Umbelliferae)* | Whole plant | ↑ Insulin | -7.5 | (Fuentes et al., 2005) |
| 433 | Bacosine | *Bacopa monnieri*  *(Scrophulariaceae)* | Aerial parts | ↓ Glucose  ↑ Glycogen | -8.2 | (Ghosh et al., 2011) |
| 434 | Bellidifolin | *Swertia punicea*  *(Gentinaceae)* | Whole plant | ↓ Glucose  ↓ Insulin  ↑ PI3K  ↑ Glycogen  ↑ G6Pase | -8 | (Tian et al., 2010) |
| 435 | Bergenin | *Caesalpinia*  *digyna (Fabaceae)* | Roots | ↓ Glucose  Regeneration of pancreatic β cell | -7.8 | (Kumar et al., 2012) |
| 436 | Beta-Sitosterol | *Dillenia indica*  *(Dilleniaceae)* | Leaves | ↓ Glucose  activation of IR and GLUT4 | -8.4 | (Ponnulakshmi et al., 2019) |
| 437 | Betulinic acid | *Tectona grandis*  *(Verbenaceae)* | Leaves | ↓ Glucose  α-amylase Improved insulin sensitivity  activation of AMP-activated protein kinase (AMPK) | -8.2 | (Birgani et al., 2018) |
| 438 | Borapetoside A | *Tinospora crispa*  *(Menispermaceae)* | Vines | ↓ IpGTT  ↑ Insulin  ↓ PEPCK | -8.5 | (Ruan et al., 2013) |
| 439 | Borapetoside C | *Tinospora crispa*  *(Menispermaceae)* | Vines | ↓ ipGTT  ↑ Insulin  ↓ PEPCK | -7.9 | (Lam et al., 2012) |
| 440 | Bruceine E | *Brucea javanica*  *(Simaroubaceae)* | Seeds | ↓ Glucose | -8.1 | (NoorShahida et al., 2009) |
| 441 | Butyl isobutyl phthalate | *Laminaria japonica (Laminariaceae)* | Rhizoids | ↓ Glucose  α-glucosidase | -6.3 | (Bu et al., 2010) |
| 442 | Caffeic acid | *Calamintha officinalis (Lamiaceae)* | Aerial parts | ↓ Glucose  ↑glucose uptake,  ↑insulin secretion | -6.4 | (Jung et al., 2006) |
| 443 | Catalpol | *Rehmannia glutinosa (Scrophulariaceae* | Roots | ↓ PEPCK  ↑ GLUT4  ↑ β-endorphin | -6.9 | (Shieh et al., 2011) |
| 444 | Cinnamaldehyde | *Cinnamonum*  *zeylanicum*  *(Lauraceae)* | Stem, Bark | ↓ Glucose  HbA1C  α-glucosidase | -5.3 | (Babu et al., 2007) |
| 445 | Cinnamic acid | *Syzygium alternifolium (Myrtaceae)* | Seeds | ↓ Glucose  improving glucose tolerance  stimulating insulin secretion | -6 | (Hafizur et al., 2015) |
| 446 | Coagulin L | *Withania coagulans (Solanaceae)* | Fruits | ↓ Glucose  Improved glucose tolerance | -9.8 | (Maurya et al., 2008) |
| 447 | Costunolide | *Costus speciosus*  *(Costaceae)* | Roots | ↑ Insulin  ↓ HbA1c | -6.9 | (Eliza et al., 2009b) |
| 448 | Cycloart-23-ene-3 β,25-diol | *Pongamia pinnata*  *(Fabaceae)* | Stem Bark | ↑HbA1c  ↑Insulin | -8.5 | (Badole & Bodhankar, 2010) |
| 449 | Decursin | *Angelica gigas*  *(Apiaceae)* | Whole plant | ↓ Glucose  Activation of glycogen synthase kinase-3 | -7.6 | (Bae et al., 2016) |
| 450 | Embelin | *Embelia ribes*  *(Myrsinaceae)* | Fruits | ↓ Glucose | -6.3 | (Naik et al., 2013) |
| 451 | Eremanthin | *Costus speciosus* | rhizomes | ↓HBA_1_c  ↑insulin | -8 | (Eliza et al., 2009a) |
| 452 | Ferulic acid | *Hibiscus mutabilis*  *(Malvaceae)* | Leaves | ↓ Glucose  improves insulin sensitivity  PEPCK G6Pase | -6.5 | (Narasimhan et al., 2015; Ohnishi et al., 2004) |
| 453 | Gallic acid | *Terminalia bellerica (Combretaceae)* | Fruits | ↑ Insulin | -5.7 | (Latha & Daisy, 2011) |
| 454 | Globularin | *Globularia alypum (Globulariaceae)* | Leaves | ↓ Glucose  ↓ TC  ↓ TG | -8.5 | (Merghache et al., 2013) |
| 455 | Guggulsterone Z | *Commiphora*  *mukul (Burseraceae)* | Resin | ↓ PEPCK  ↑ PPARγ  ↓ Aldose  reductase | -8.8 | (B. Sharma et al., 2009) |
| 456 | Hydrangenol | *Hydrangea*  *macrophylla*  *(Hydrangeaceae)* | Leaves | ↓ Glucose  ↓ FFA  PPARγ2  GLUT4 | -8.4 | (H. Zhang et al., 2007) |
| 457 | Isoliquiritigenin | *Glycyrrhiza*  *glabra (Fabaceae)* | Rhizomes | ↓ Glucose | -7.3 | (Gaur et al., 2014) |
| 458 | Lsoorientin | *Cecropia pachystachya*  *(Urticaceae)* | Leaves | Activate insulin signaling pathway | -7.9 | (Alonso-Castro et al., 2012) |
| 459 | Isovitexin | *Ficus deltoidea*  *(Moraceae)* | Leaves | ↓ α-glucosidase  SGLT2 | -7.9 | (Shi et al., 2019) |
| 460 | Karanjin | *Pongamia pinnata*  *(Fabaceae)* | Fruits | ↓ Glucose  ↓tyrosine phosphatase-1B | -7.6 | (Mandal & Maity, 1986) |
| 461 | Kolaviron | *Garcinia kola*  *(Clusiaceae)* | Seeds | ↓ Hb1Ac  α -Amylase | -8.6 | (Adaramoye, 2012) |
| 462 | Licochalcone E | *Glycyrrhiza*  *inflata (Fabaceae)* | Roots | ↑ PPARγ | -7.1 | (Park et al., 2012) |
| 463 | Lucidin 3-O-beta-D-primeveroside | *Morinda citrifolia*  *(Rubiaceae)* | Roots | ↓ Glucose | -8.6 | (Nerurkar et al., 2015) |
| 464 | Lupenone | *Musa basjoo*  *(Musaceae)* | Rhizomes | ↓ HbA1c | -8.6 | (Xu et al., 2014) |
| 465 | Magnolol | *Magnolia officinalis (Magnoliaceae)* | Root Barks | ↓ Glucose  ↓ Insulin  ↓ TGF- β1  hepatic insulin resistance | -7.2 | (Wang et al., 2014) |
| 466 | Mahanimbine | *Murraya koenigii*  *(Rutaceae)* | Leaves | α-glucosidase  α -Amylase | -8.7 | (Mitra & Mahadevappa, 2010) |
| 467 | Mahanine | *Murraya koenigii*  *(Rutaceae)* | Leaves | ↓ Glucose  Insulin resistance gene expression  NF-kB pathway | -8.9 | (Biswas et al., 2010) |
| 468 | Marrubiin | *Leonotis leonurus*  *(Lamiaceae)* | Leaves | ↓ ipGTT  ↑ Insulin | -7.3 | (Mnonopi et al., 2012) |
| 469 | Maslinic acid | *Syzygium aromaticum (Myrtaceae)* | Flowers | α-Glucosidase  α-Amylase | -9.2 | (Khathi et al., 2013) |
| 470 | Methyl caffeate | *Solanum torvum*  *(Solanaceae)* | Fruits | ↓ HbA1c  ↑ Insulin | -6 | (Gandhi et al., 2011) |
| 471 | Moracin M | *Morus alba*  *(Moraceae)* | Root Bark | ↓ Glucose | -7.6 | (M. Zhang et al., 2009) |
| 472 | Morolic acid | *Phoradendron*  *reichenbachianum*  *(Loranthaceae)* | Aerial parts | inhibition of 11-HSD  PTP1B inhibition | -8.9 | (Rani et al., 2021) |
| 472 | Moronic acid | *Phoradendron*  *reichenbachianum*  *(Loranthaceae)* | Aerial parts | ↓ Glucose absorption  PTP-1B  11β-HSD 1 | -8.9 | (Rani et al., 2021) |
| 473 | Mulinolic acid | *Azorella compacta*  *(Umbelliferae)* | Whole plant | ↑ Insulin | -6.8 | (Fuentes et al., 2005) |
| 474 | Multiflorin A | *Prunus persica*  *(Rosaceae)* | Leaves | ↓glucose  absorption | -8 | (Shirosaki et al., 2012) |
| 475 | Nicotinic acid | *Cucurbita sp.*  *(Cucurbitaceae)* | Fruits | ↓ HbA1c  ↑ Insulin | -5.3 | (Yoshinari et al., 2009) |
| 475 | Nymphayol | *Nymphaea stellata*  *(Nymphaeaceae)* | Flowers | ↑ Insulin | -8.1 | (Subash-Babu et al., 2009) |
| 476 | Oleanolic acid | *Syzygium aromaticum (Myrtaceae)* | Flowers | α-Glucosidase  α-Amylase | -8.8 | (Khathi et al., 2013) |
| 477 | Plumbagin | *Plumbago zeylanica (Plumbaginaceae)* | Roots | ↑ Insulin  ↑ Glycogen | -6.8 | (Sunil et al., 2012) |
| 478 | Polymatin A | *Smallanthus*  *macroscyphus*  *(Asteraceae)* | Leaves | ↓ HbA1c | -7.6 | (Serra-Barcellona et al., 2014) |
| 479 | Quercetin 3-(6-malonylglucoside) | *Morus alba*  *(Moraceae)* | Leaves | ↓ Glucose | -7.9 | (Katsube et al., 2010) |
| 480 | Rhaponticin | *Rheum franzenbachii (Polygonaceae)* | Rhizomes | ↓ Glucose  enhanced insulin-stimulated glucose uptake | -8.4 | (Chen et al., 2009) |
| 481 | Rutin | *Morus alba*  *(Moraceae)* | Leaves | ↓ Glucose increase the secretion of insulin  α-glucosidases α-amylase  expression of PPARγ | -7.9 | (Ghorbani & Pharmacotherapy, 2017) |
| 482 | Shikimic acid | *Juniperus oxycedrus (Cupressaceae)* | Berries | ↓ Glucose | -5.7 | (Orhan et al., 2012) |
| 483 | Steppogenin-4'-O-beta-D-glucosiade | *Morus alba*  *(Moraceae)* | Root, Bark | ↓ Glucose | -9.2 | (M. Zhang et al., 2009) |
| 484 | Stigmasterol | *Dillenia indica*  *(Dilleniaceae)* | Leaves | α-Glucosidase  α-Amylase | -8.7 | (S. Kumar et al., 2013) |
| 485 | Swertiamarin | *Enicostemma*  *littorale (Gentianaceae)* | Whole plant | ↓ Glucose  PPAR-ᵧ Gene Expression | -7.5 | (Vaidya et al., 2013) |
| 486 | Swertisin | *Wilbrandia ebracteata (Cucurbitaceae)* | Roots | ↑ Glucose  uptake  ↑ Insulin | -7.8 | (Folador et al., 2010) |
| 487 | Swietenine | *Swietenia macrophylla (Meliaceae)* | Seeds | ↓ Glucose | -8.5 | (Dewanjee et al., 2009) |
| 488 | Syringin | *Musa paradisíac*  *(Musaceae)* | Flowers | ↑ Insulin  ↓ HbA1c | -7.3 | (Krishnan et al., 2014) |
| 489 | Trans-tiliroside | *Potentilla chinesis*  *(Rosaceae)* | Whole plant | ↓ Glucose  HepG2 cell | -8.2 | (Zhu et al., 2010) |
| 490 | Trigonelline | *Cucurbita sp.*  *(Cucurbitaceae)* | Fruits | ↓ HbA1c  ↑ Insulin | -5.4 | (Yoshinari et al., 2009) |
| 491 | Ursolic acid | *Leandra lacunosa*  *Cornus officinalis* | aerial  parts  whole plant | GLUT4  PI3K/AKT | -8.9 | (Castro et al., 2015; D. Gao et al., 2008; He et al., 2014) |
| 492 | Valoneic acid dilactone | *Punica granatum*  *(Punicaceae)* | Fruits | Aldose reductase, α-amylase, PTP1B | -9.2 | (Jain et al., 2012) |
| 493 | Vitexin | *Ficus deltoida* | Leaves | α-glucosidase | -7.9 | (Choo et al., 2012) |
| 494 | Xanthorrhizol | *Curcuma xanthorrhiza (Zingiberaceae)* | Rhizomes | ↓ Glucose  ↓ Insulin  OGTT | -6.5 | (Kim et al., 2014) |

**Supplementary Table 3:** Protein-ligand interactions of the top ten compounds

| **Compounds** | **Pubchem ID** | **Docking score (Kcal/mol)** | **Category** | **Bond type** | **Interacting amino acids** | **Interacting Chain** | **Distance**  **(Å)** |
| --- | --- | --- | --- | --- | --- | --- | --- |
| Corilagin | 73568 | -10.2 | Electrostatic | Pi-cation | Lys122 | A | 3.83085 |
|  |  |  | Hydrophobic | Pi-Pi stacked | Trp127 | B | 3.65756 |
|  |  |  |  |  | Trp127 | B | 4.04405 |
|  |  |  |  | Pi-alkyl | Lys51 | A | 4.48778 |
|  |  |  | H bond | Conventional | Ser47 | A | 2.78863 |
|  |  |  |  |  | Arg58 | A | 2.01848 |
|  |  |  |  |  | Arg58 | A | 2.85764 |
|  |  |  |  |  | Asn124 | B | 2.37148 |
|  |  |  |  |  | Asn124 | B | 2.0454 |
|  |  |  |  |  | Arg128 | B | 2.69814 |
|  |  |  |  | Carbon H bond | Lys51 | A | 3.17068 |
| Stachyurin | 157395 | -10.4 | Electrostatic | Pi-cation | Lys122 | A | 3.57021 |
|  |  |  | Hydrophobic | Pi-alkyl | Lys51 | A | 4.62083 |
|  |  |  | H bond | Conventional | Asn52 | A | 2.17891 |
|  |  |  |  |  | Lys122 | A | 2.52592 |
|  |  |  |  |  | Arg129 | A | 2.99649 |
|  |  |  |  |  | Tyr130 | A | 2.41335 |
|  |  |  |  |  | Asn123 | B | 2.26235 |
|  |  |  |  |  | Asn124 | B | 2.28086 |
|  |  |  |  |  | Asp126 | A | 2.79142 |
|  |  |  |  |  | Asn175 | A | 2.22646 |
|  |  |  |  |  | Asn52 | A | 1.78616 |
|  |  |  | Electrostatic; H bond | Pi-cation; Pi-donor hydrogen bond | Lys51 | A | 2.78967 |
|  |  |  |  |  | Arg128 | B | 3.86259 |
| Dieckol | 3008868 | -10.8 | Electrostatic | Pi-cation | Lys122 | A | 3.61661 |
|  |  |  |  |  | Lys122 | A | 3.68935 |
|  |  |  |  | Pi-anion | Asp126 | A | 3.78191 |
|  |  |  | Hydrophobic | Pi-Pi stacked | Trp127 | B | 3.82949 |
|  |  |  |  |  | Trp127 | B | 4.20872 |
|  |  |  |  |  | Trp127 | B | 4.77809 |
|  |  |  |  |  | Trp127 | B | 4.0851 |
|  |  |  |  |  | Trp127 | B | 4.71852 |
|  |  |  |  | Amide-Pi stacked | Gly55, Ala56 | A | 3.74587 |
|  |  |  |  | Pi-alkyl | Ala56 | A | 5.18136 |
|  |  |  |  |  | Lys51 | A | 4.39167 |
|  |  |  | H bond | Conventional | Ser59 | A | 2.95046 |
|  |  |  |  |  | Asn175 | A | 2.38547 |
|  |  |  |  |  | Asn123 | B | 2.68951 |
|  |  |  |  |  | Tyr127 | A | 2.82641 |
| Isocorilagin | 10077799 | -10.6 | Electrostatic | Pi-cation | Lys122 | A | 3.39032 |
|  |  |  |  | Pi-anion | Asp126 | A | 3.935 |
|  |  |  | Hydrophobic | Pi-Pi stacked | Trp127 | B | 3.97624 |
|  |  |  |  |  | Trp127 | B | 3.80873 |
|  |  |  |  | Pi-alkyl | Lys51 | A | 4.76213 |
|  |  |  | H bond | Conventional | Lys51 | A | 2.3359 |
|  |  |  |  |  | Lys51 | A | 2.77127 |
|  |  |  |  |  | Arg58 | A | 2.0223 |
|  |  |  |  |  | Asn124 | B | 2.21445 |
|  |  |  |  |  | Ser47 | A | 2.15421 |
|  |  |  |  | Carbon H bond | Lys51 | A | 3.66903 |
| Thonningianin A | 10328286 | -11.3 | Electrostatic | Pi-cation | Lys122 | A | 3.35562 |
|  |  |  | Hydrophobic | Pi-Pi stacked | Trp127 | B | 3.8754 |
|  |  |  |  |  | Trp127 | B | 4.84084 |
|  |  |  |  | Pi-Pi T-shaped | Phe119 | A | 5.40565 |
|  |  |  |  | Pi-alkyl | Lys51 | A | 4.91785 |
|  |  |  |  |  | Lys51 | A | 5.46729 |
|  |  |  |  |  | Leu174 | A | 5.43253 |
|  |  |  | Hydrogen | Conventional | Lys51 | A | 2.46072 |
|  |  |  |  |  | Lys51 | A | 2.04256 |
|  |  |  |  |  | Arg129 | A | 2.78603 |
|  |  |  |  |  | Asp126 | A | 2.09122 |
| Lactucain | 10896414 | -10.7 | Hydrogen | Conventional | Arg58 | A | 2.84275 |
|  |  |  |  |  | Arg58 | A | 2.3116 |
|  |  |  |  |  | Lys122 | A | 2.67226 |
|  |  |  |  |  | Arg129 | A | 2.6298 |
|  |  |  |  |  | Trp127 | B | 2.67482 |
|  |  |  |  |  | Arg128 | B | 2.24639 |
|  |  |  | Hydrophobic | Pi-sigma | Trp127 | B | 3.47664 |
| Gypensapogenin A | 57403926 | -10.2 | Hydrogen | Conventional | Lys51 | A | 2.64985 |
|  |  |  | Hydrophobic | Pi-sigma | Trp127 | B | 3.77401 |
|  |  |  |  | Alkyl | Leu218 | A | 5.3454 |
|  |  |  |  |  | Ile219 | A | 5.1807 |
|  |  |  |  |  | Ile120 | B | 5.45902 |
|  |  |  |  | Pi-alkyl | Trp127 | B | 4.98698 |
|  |  |  |  |  | Trp127 | B | 3.97678 |
| Stachysetin | 101682257 | -10.4 | Hydrogen | Conventional | Lys51 | A | 2.69362 |
|  |  |  |  |  | Lys51 | A | 2.89485 |
|  |  |  |  |  | Arg129 | A | 2.44346 |
|  |  |  |  |  | Arg129 | A | 2.08052 |
|  |  |  |  |  | Arg129 | A | 2.50627 |
|  |  |  |  |  | Asn175 | A | 2.48795 |
|  |  |  |  |  | Asn123 | B | 2.88818 |
|  |  |  |  |  | Asn124 | B | 2.2382 |
|  |  |  |  |  | Tyr130 | A | 2.3895 |
|  |  |  |  | Pi-donor H bond | Trp127 | B | 2.59029 |
|  |  |  | Hydrophobic | Pi-sigma | Lys51 | A | 3.6235 |
|  |  |  |  |  | Ala56 | A | 3.49764 |
|  |  |  |  |  | Ile120 | B | 3.72732 |
|  |  |  |  | Pi-alkyl | Lys51 | A | 4.45253 |
|  |  |  |  |  | Val48 | A | 5.28361 |
|  |  |  |  |  | Lys51 | A | 4.98297 |
|  |  |  |  |  | Ile219 | A | 5.48291 |
|  |  |  |  |  | Ile120 | B | 5.49501 |
| Kaempferol-3-rutinoside | 122173234 | -10.1 | Hydrophobic | Pi-sigma | Ile168 | A | 3.98088 |
|  |  |  |  |  | Ile219 | A | 3.94948 |
|  |  |  |  | Pi-alkyl | Pro167 | A | 5.12042 |
|  |  |  | H bond | Conventional | Asn44 | A | 2.76498 |
|  |  |  |  |  | Asn44 | A | 1.86276 |
|  |  |  |  |  | Asn44 | A | 2.82819 |
|  |  |  |  |  | Ser47 | A | 2.14358 |
|  |  |  |  |  | Asp215 | A | 2.02503 |
| Cupressuflavone | 5281609 | -9.9 | Hydrophobic | Pi-sigma | Ile120 | B | 3.93596 |
|  |  |  |  | Pi-Pi stacked | Trp127 | B | 4.10085 |
|  |  |  |  |  | Trp127 | B | 3.72373 |
|  |  |  |  | Pi-alkyl | Lys51 | A | 3.85102 |
|  |  |  | H bond | Conventional | Ser47 | A | 2.32537 |
|  |  |  |  |  | Arg128 | B | 1.82935 |
|  |  |  |  |  | Gly55 | A | 2.6428 |
|  |  |  |  | Pi-donor | Asn124 | B | 2.92648 |
|  |  |  | H bond; Electrostatic | Pi-cation; Pi-donor H bond | Lys51 | A | 2.89853 |

**References:**

Abdelsalam, S. S., Korashy, H. M., Zeidan, A., & Agouni, A. (2019, Jul 17). The Role of Protein Tyrosine Phosphatase (PTP)-1B in Cardiovascular Disease and Its Interplay with Insulin Resistance. *Biomolecules, 9*(7). <https://doi.org/10.3390/biom9070286>

Ablat, A., Halabi, M. F., Mohamad, J., Hasnan, M. H. H., Hazni, H., Teh, S.-h., Shilpi, J. A., Mohamed, Z., Awang, K. J. B. c., & medicine, a. (2017). Antidiabetic effects of Brucea javanica seeds in type 2 diabetic rats. *17*(1), 1-14.

Adachi, Y., Kanbayashi, Y., Harata, I., Ubagai, R., Takimoto, T., Suzuki, K., Miwa, T., & Noguchi, Y. (2014). Petasin activates AMP-activated protein kinase and modulates glucose metabolism. *Journal of natural products, 77*(6), 1262-1269.

Adaramoye, O. A. (2012, Dec). Antidiabetic effect of kolaviron, a biflavonoid complex isolated from Garcinia kola seeds, in Wistar rats. *Afr Health Sci, 12*(4), 498-506. <https://doi.org/10.4314/ahs.v12i4.16>

Ahamad, J. J. I. R. J. O. P. (2019). ANTIHYPERGLYCEMIC ACTIVITY OF CHARANTIN ISOLATED FROM FRUITS OF MOMORDICA CHARANTIA LINN. *10*(1), 61-64.

Ahmed, A. B., Rao, A. S., & Rao, M. V. (2010, Nov). In vitro callus and in vivo leaf extract of Gymnema sylvestre stimulate β-cells regeneration and anti-diabetic activity in Wistar rats. *Phytomedicine, 17*(13), 1033-1039. <https://doi.org/10.1016/j.phymed.2010.03.019>

Ahmed, D., Kumar, V., Sharma, M., & Verma, A. (2014, May 13). Target guided isolation, in-vitro antidiabetic, antioxidant activity and molecular docking studies of some flavonoids from Albizzia Lebbeck Benth. bark. *BMC Complement Altern Med, 14*, 155. <https://doi.org/10.1186/1472-6882-14-155>

Ajikumaran Nair, S., Shylesh, B. S., Gopakumar, B., & Subramoniam, A. (2006, Jun 30). Anti-diabetes and hypoglycaemic properties of Hemionitis arifolia (Burm.) Moore in rats. *J Ethnopharmacol, 106*(2), 192-197. <https://doi.org/10.1016/j.jep.2005.12.020>

Ali, M. Y., Kim, D. H., Seong, S. H., Kim, H. R., Jung, H. A., & Choi, J. S. (2017, Dec 1). α-Glucosidase and Protein Tyrosine Phosphatase 1B Inhibitory Activity of Plastoquinones from Marine Brown Alga Sargassum serratifolium. *Mar Drugs, 15*(12). <https://doi.org/10.3390/md15120368>

Ali, S., Igoli, J., Clements, C., Alamzeb, M., Shah, S. Q., Ferro, V. A., Gray, A. I., Khan, M. R. J., & Pharmacology, B. J. o. (2013). Anti-diabetic and antimicrobial activities of fractions and compounds isolated from Berberis brevissima Jafri and Berberis parkeriana Schneid. *Bangladesh Journal of Pharmacology, 8*(3), 336-342.

Alonso-Castro, A. J., Zapata-Bustos, R., Gómez-Espinoza, G., & Salazar-Olivo, L. A. J. E. (2012). Isoorientin reverts TNF-α-induced insulin resistance in adipocytes activating the insulin signaling pathway. *153*(11), 5222-5230.

Anaya-Eugenio, G. D., Rivero-Cruz, I., Rivera-Chávez, J., & Mata, R. (2014, Aug 8). Hypoglycemic properties of some preparations and compounds from Artemisia ludoviciana Nutt. *J Ethnopharmacol, 155*(1), 416-425. <https://doi.org/10.1016/j.jep.2014.05.051>

Anhut, S., Biehl, J., Seeger, T., Mues, R., & Zinsmeister, H. D. J. Z. f. N. C. (1992). Flavone-C-Glycosides from the Mosses Plagiomnium elatum and Plagiomnium cuspidatum. *Zeitschrift für Naturforschung C, 47*(9-10), 654-660.

Arha, D., Pandeti, S., Mishra, A., Srivastava, S. P., Srivastava, A. K., Narender, T., & Tamrakar, A. K. (2015, Dec 5). Deoxyandrographolide promotes glucose uptake through glucose transporter-4 translocation to plasma membrane in L6 myotubes and exerts antihyperglycemic effect in vivo. *Eur J Pharmacol, 768*, 207-216. <https://doi.org/10.1016/j.ejphar.2015.10.055>

Arvindekar, A., More, T., Payghan, P. V., Laddha, K., Ghoshal, N., & Arvindekar, A. (2015, Aug). Evaluation of anti-diabetic and alpha glucosidase inhibitory action of anthraquinones from Rheum emodi. *Food Funct, 6*(8), 2693-2700. <https://doi.org/10.1039/c5fo00519a>

Asghari, B., Salehi, P., Farimani, M. M., & Ebrahimi, S. N. J. R. o. n. p. (2015). α-Glucosidase Inhibitors from Fruits of Rosa canina L. *Records of natural products, 9*(3).

Asghari, B., Salehi, P., Sonboli, A., & Nejad Ebrahimi, S. (2015, Spring). Flavonoids from Salvia chloroleuca with α-Amylsae and α-Glucosidase Inhibitory Effect. *Iran J Pharm Res, 14*(2), 609-615.

Asolkar, R. N., Freel, K. C., Jensen, P. R., Fenical, W., Kondratyuk, T. P., Park, E. J., & Pezzuto, J. M. (2009, Mar 27). Arenamides A-C, cytotoxic NFkappaB inhibitors from the marine actinomycete Salinispora arenicola. *J Nat Prod, 72*(3), 396-402. <https://doi.org/10.1021/np800617a>

B Gaikwad, S., Krishna Mohan, G., & Sandhya Rani, M. (2014). Phytochemicals for diabetes management. *Pharmaceutical Crops, 5*(1).

Babu, P. S., Prabuseenivasan, S., & Ignacimuthu, S. J. P. (2007). Cinnamaldehyde—a potential antidiabetic agent. *14*(1), 15-22.

Badole, S. L., & Bodhankar, S. L. (2010, Apr 25). Antidiabetic activity of cycloart-23-ene-3beta, 25-diol (B2) isolated from Pongamia pinnata (L. Pierre) in streptozotocin-nicotinamide induced diabetic mice. *Eur J Pharmacol, 632*(1-3), 103-109. <https://doi.org/10.1016/j.ejphar.2010.01.019>

Bae, E. Y., Na, M., Njamen, D., Mbafor, J. T., Fomum, Z. T., Cui, L., Choung, D. H., Kim, B. Y., Oh, W. K., & Ahn, J. S. (2006, Aug). Inhibition of protein tyrosine phosphatase 1B by prenylated isoflavonoids isolated from the stem bark of Erythrina addisoniae. *Planta Med, 72*(10), 945-948. <https://doi.org/10.1055/s-2006-946674>

Bae, U.-J., Choi, E.-K., Oh, M.-R., Jung, S.-J., Park, J., Jung, T.-S., Park, T.-S., Chae, S.-W., & Park, B.-H. J. T. A. j. o. C. m. (2016). Angelica gigas ameliorates hyperglycemia and hepatic steatosis in C57BL/KsJ-db/db mice via activation of AMP-activated protein kinase signaling pathway. *44*(08), 1627-1638.

Bai, N., He, K., Ibarra, A., Bily, A., Roller, M., Chen, X., & Rühl, R. (2010, Jan). Iridoids from Fraxinus excelsior with adipocyte differentiation-inhibitory and PPARalpha activation activity. *J Nat Prod, 73*(1), 2-6. <https://doi.org/10.1021/np9003118>

Bai, N., He, K., Ibarra, A., Bily, A., Roller, M., Chen, X., & Rühl, R. J. J. o. N. P. (2010). Iridoids from Fraxinus excelsior with adipocyte differentiation-inhibitory and PPARα activation activity. *J Nat Prod, 73*(1), 2-6.

Bai, N., He, K., Roller, M., Zheng, B., Chen, X., Shao, Z., Peng, T., & Zheng, Q. (2008, Dec 24). Active compounds from Lagerstroemia speciosa, insulin-like glucose uptake-stimulatory/inhibitory and adipocyte differentiation-inhibitory activities in 3T3-L1 cells. *J Agric Food Chem, 56*(24), 11668-11674. <https://doi.org/10.1021/jf802152z>

Bajpai, V. K., Park, Y. H., Na, M., & Kang, S. C. (2015, Mar 26). α-Glucosidase and tyrosinase inhibitory effects of an abietane type diterpenoid taxoquinone from Metasequoia glyptostroboides. *BMC Complement Altern Med, 15*, 84. <https://doi.org/10.1186/s12906-015-0626-3>

Balamurugan, R., Duraipandiyan, V., & Ignacimuthu, S. J. E. j. o. p. (2011). Antidiabetic activity of γ-sitosterol isolated from Lippia nodiflora L. in streptozotocin induced diabetic rats. *667*(1-3), 410-418.

Balamurugan, R., Vendan, S. E., Aravinthan, A., & Kim, J. H. (2015, Apr). Isolation and structural characterization of 2R, 3R taxifolin 3-O-rhamnoside from ethyl acetate extract of Hydnocarpus alpina and its hypoglycemic effect by attenuating hepatic key enzymes of glucose metabolism in streptozotocin-induced diabetic rats. *Biochimie, 111*, 70-81. <https://doi.org/10.1016/j.biochi.2015.02.003>

Banskota, A. H., Nguyen, N. T., Tezuka, Y., Nobukawa, T., & Kadota, S. (2006, Jan). Hypoglycemic effects of the wood of Taxus yunnanensis on streptozotocin-induced diabetic rats and its active components. *Phytomedicine, 13*(1-2), 109-114. <https://doi.org/10.1016/j.phymed.2004.01.015>

Barde, S. R., Sakhare, R. S., Kanthale, S. B., Chandak, P. G., & Jamkhande, P. G. J. A. P. J. o. T. D. (2015). Marine bioactive agents: A short review on new marine antidiabetic compounds. *Asian Pacific Journal of Tropical Disease, 5*, S209-S213.

Begmatov, N., Li, J., Bobakulov, K., Numonov, S., & Aisa, H. A. (2020, Jun). The chemical components of Coreopsis tinctoria Nutt. and their antioxidant, antidiabetic and antibacterial activities. *Nat Prod Res, 34*(12), 1772-1776. <https://doi.org/10.1080/14786419.2018.1525377>

Bidon-Chanal, A., Fuertes, A., Alonso, D., Pérez, D. I., Martínez, A., Luque, F. J., & Medina, M. (2013, Feb). Evidence for a new binding mode to GSK-3: allosteric regulation by the marine compound palinurin. *Eur J Med Chem, 60*, 479-489. <https://doi.org/10.1016/j.ejmech.2012.12.014>

Birgani, G. A., Ahangarpour, A., Khorsandi, L., & Moghaddam, H. F. J. B. J. o. P. S. (2018). Anti-diabetic effect of betulinic acid on streptozotocin-nicotinamide induced diabetic male mouse model. *54*.

Biswas, A., Bhattacharya, S., Dasgupta, S., Kundu, R., Roy, S. S., Pal, B. C., & Bhattacharya, S. (2010, Mar). Insulin resistance due to lipid-induced signaling defects could be prevented by mahanine. *Mol Cell Biochem, 336*(1-2), 97-107. <https://doi.org/10.1007/s11010-009-0257-4>

Bu, T., Liu, M., Zheng, L., Guo, Y., & Lin, X. (2010, Nov). α-Glucosidase inhibition and the in vivo hypoglycemic effect of butyl-isobutyl-phthalate derived from the Laminaria japonica rhizoid. *Phytother Res, 24*(11), 1588-1591. <https://doi.org/10.1002/ptr.3139>

Casanova, L. M., Gu, W., Costa, S. n. S., & Jeppesen, P. B. (2017). Phenolic substances from Ocimum species enhance glucose-stimulated insulin secretion and modulate the expression of key insulin regulatory genes in mice pancreatic islets. *Journal of natural products, 80*(12), 3267-3275.

Castro, A. J. G., Frederico, M. J. S., Cazarolli, L. H., Mendes, C. P., Bretanha, L. C., Schmidt, E. C., Bouzon, Z. L., de Medeiros Pinto, V. A., da Fonte Ramos, C., & Pizzolatti, M. G. J. B. e. B. A.-G. S. (2015). The mechanism of action of ursolic acid as insulin secretagogue and insulinomimetic is mediated by cross-talk between calcium and kinases to regulate glucose balance. *1850*(1), 51-61.

Cazarolli, L. H., Kappel, V. D., Pereira, D. F., Moresco, H. H., Brighente, I. M., Pizzolatti, M. G., & Silva, F. R. (2012, Oct). Anti-hyperglycemic action of apigenin-6-C-β-fucopyranoside from Averrhoa carambola. *Fitoterapia, 83*(7), 1176-1183. <https://doi.org/10.1016/j.fitote.2012.07.003>

Chang, C.-I., Hsu, C.-M., Li, T.-S., Huang, S.-D., Lin, C.-C., Yen, C.-H., Chou, C.-H., & Cheng, H.-L. J. j. o. f. f. (2014). Constituents of the stem of Cucurbita moschata exhibit antidiabetic activities through multiple mechanisms. *journal of functional foods, 10*, 260-273.

Chauhan, A., Sharma, P., Srivastava, P., Kumar, N., & Dudhe, R. J. D. P. L. (2010). Plants having potential antidiabetic activity: a review. *2*(3), 369-387.

Chen, H. Y., Ye, X. L., Cui, X. L., He, K., Jin, Y. N., Chen, Z., & Li, X. G. (2012, Jan). Cytotoxicity and antihyperglycemic effect of minor constituents from Rhizoma Coptis in HepG2 cells. *Fitoterapia, 83*(1), 67-73. <https://doi.org/10.1016/j.fitote.2011.09.014>

Chen, J., Ma, M., Lu, Y., Wang, L., Wu, C., & Duan, H. (2009, Apr). Rhaponticin from rhubarb rhizomes alleviates liver steatosis and improves blood glucose and lipid profiles in KK/Ay diabetic mice. *Planta Med, 75*(5), 472-477. <https://doi.org/10.1055/s-0029-1185304>

Chen, J., Mangelinckx, S., Ma, L., Wang, Z., Li, W., & De Kimpe, N. (2014, Dec). Caffeoylquinic acid derivatives isolated from the aerial parts of Gynura divaricata and their yeast α-glucosidase and PTP1B inhibitory activity. *Fitoterapia, 99*, 1-6. <https://doi.org/10.1016/j.fitote.2014.08.015>

Chen, Q. B., Xin, X. L., Yang, Y., Lee, S. S., & Aisa, H. A. (2014, Apr 25). Highly conjugated norditerpenoid and pyrroloquinoline alkaloids with potent PTP1B inhibitory activity from Nigella glandulifera. *J Nat Prod, 77*(4), 807-812. <https://doi.org/10.1021/np4009078>

Cho, J.-Y., Lee, K.-D., Park, S.-Y., Jeong, W. C., Moon, J.-H., & Ham, K.-S. (2013). Isolation and identification of α-glucosidase inhibitors from the stem bark of the nutgall tree (Rhus javanica Linné). *Journal of the Korean Society for Applied Biological Chemistry, 56*(5), 547-552.

Choi, C. W., Choi, Y. H., Cha, M. R., Yoo, D. S., Kim, Y. S., Yon, G. H., Hong, K. S., Kim, Y. H., & Ryu, S. Y. (2010, Sep 22). Yeast α-glucosidase inhibition by isoflavones from plants of Leguminosae as an in vitro alternative to acarbose. *J Agric Food Chem, 58*(18), 9988-9993. <https://doi.org/10.1021/jf101926j>

Choi, J. S., Ali, M. Y., Jung, H. A., Oh, S. H., Choi, R. J., & Kim, E. J. J. J. o. e. (2015). Protein tyrosine phosphatase 1B inhibitory activity of alkaloids from Rhizoma Coptidis and their molecular docking studies. *171*, 28-36.

Choi, S. B., Ko, B. S., Park, S. K., Jang, J. S., & Park, S. (2006, Jan 25). Insulin sensitizing and alpha-glucoamylase inhibitory action of sennosides, rheins and rhaponticin in Rhei Rhizoma. *Life Sci, 78*(9), 934-942. <https://doi.org/10.1016/j.lfs.2005.05.101>

Choi, S. S., Cha, B. Y., Iida, K., Sato, M., Lee, Y. S., Teruya, T., Yonezawa, T., Nagai, K., & Woo, J. T. (2011, Jul). Honokiol enhances adipocyte differentiation by potentiating insulin signaling in 3T3-L1 preadipocytes. *J Nat Med, 65*(3-4), 424-430. <https://doi.org/10.1007/s11418-011-0512-3>

Choo, C., Sulong, N., Man, F., & Wong, T. J. J. o. e. (2012). Vitexin and isovitexin from the leaves of Ficus deltoidea with in-vivo α-glucosidase inhibition. *142*(3), 776-781.

Choudhary, M. I., Adhikari, A., Rasheed, S., Marasini, B. P., Hussain, N., & Kaleem, W. A. J. P. l. (2011). Cyclopeptide alkaloids of Ziziphus oxyphylla Edgw as novel inhibitors of α-glucosidase enzyme and protein glycation. *Phytochemistry letters, 4*(4), 404-406.

Chowdhury, S. S., Islam, M. N., Jung, H. A., & Choi, J. S. (2014, Jan-Feb). In vitro antidiabetic potential of the fruits of Crataegus pinnatifida. *Res Pharm Sci, 9*(1), 11-22.

Christensen, K. B., Petersen, R. K., Petersen, S., Kristiansen, K., & Christensen, L. P. (2009, May 22). Activation of PPARgamma by metabolites from the flowers of purple coneflower (Echinacea purpurea). *J Nat Prod, 72*(5), 933-937. <https://doi.org/10.1021/np900003a>

Cui, J., Xi, M.-M., Li, Y.-W., Duan, J.-L., Wang, L., Weng, Y., Jia, N., Cao, S.-S., Li, R.-L., & Wang, C. J. J. o. e. (2015). Insulinotropic effect of Chikusetsu saponin IVa in diabetic rats and pancreatic β-cells. *164*, 334-339.

Cui, L., Lee, H. S., Ndinteh, D. T., Mbafor, J. T., Kim, Y. H., Le, T. V. T., Nguyen, P. H., & Oh, W. K. J. P. m. (2010). New prenylated flavanones from Erythrina abyssinica with protein tyrosine phosphatase 1B (PTP1B) inhibitory activity. *Planta medica, 76*(07), 713-718.

Cunha, W. R., Arantes, G. M., Ferreira, D. S., Lucarini, R., Silva, M. L., Furtado, N. A., da Silva Filho, A. A., Crotti, A. E., & Araújo, A. R. (2008, Jul). Hypoglicemic effect of Leandra lacunosa in normal and alloxan-induced diabetic rats. *Fitoterapia, 79*(5), 356-360. <https://doi.org/10.1016/j.fitote.2008.04.002>

Daisy, P., Balasubramanian, K., Rajalakshmi, M., Eliza, J., & Selvaraj, J. (2010, Jan). Insulin mimetic impact of Catechin isolated from Cassia fistula on the glucose oxidation and molecular mechanisms of glucose uptake on Streptozotocin-induced diabetic Wistar rats. *Phytomedicine, 17*(1), 28-36. <https://doi.org/10.1016/j.phymed.2009.10.018>

Damsud, T., Adisakwattana, S., & Phuwapraisirisan, P. J. P. L. (2013). Three new phenylpropanoyl amides from the leaves of Piper sarmentosum and their α-glucosidase inhibitory activities. *Phytochemistry Letters, 6*(3), 350-354.

Dang, P. H., Nguyen, N. T., Nguyen, H. X., Nguyen, L. B., Le, T. H., Do, T. N., Can, M. V., & Nguyen, M. T. (2015, Jan). α-Glucosidase inhibitors from the leaves of Embelia ribes. *Fitoterapia, 100*, 201-207. <https://doi.org/10.1016/j.fitote.2014.12.004>

Dewanjee, S., Maiti, A., Das, A. K., Mandal, S. C., & Dey, S. P. (2009, Jun). Swietenine: a potential oral hypoglycemic from Swietenia macrophylla seed. *Fitoterapia, 80*(4), 249-251. <https://doi.org/10.1016/j.fitote.2009.02.004>

Ding, Z., Lu, Y., Lu, Z., Lv, F., Wang, Y., Bie, X., Wang, F., & Zhang, K. J. F. c. (2010). Hypoglycaemic effect of comatin, an antidiabetic substance separated from Coprinus comatus broth, on alloxan-induced-diabetic rats. *Food chemistry, 121*(1), 39-43.

Divya, M., Karthikeyan, S., Ravi, C., Govindarajan, M., Alharbi, N. S., Kadaikunnan, S., Khaled, J. M., Almanaa, T. N., & Vaseeharan, B. J. M. p. (2020). Isolation of β-glucan from Eleusine coracana and its antibiofilm, antidiabetic, antioxidant, and biocompatible activities. *140*, 103955.

Do, G. M., Jung, U. J., Park, H. J., Kwon, E. Y., Jeon, S. M., McGregor, R. A., & Choi, M. S. (2012, Aug). Resveratrol ameliorates diabetes-related metabolic changes via activation of AMP-activated protein kinase and its downstream targets in db/db mice. *Mol Nutr Food Res, 56*(8), 1282-1291. <https://doi.org/10.1002/mnfr.201200067>

Eid, H. M., Martineau, L. C., Saleem, A., Muhammad, A., Vallerand, D., Benhaddou-Andaloussi, A., Nistor, L., Afshar, A., Arnason, J. T., & Haddad, P. S. (2010, Jul). Stimulation of AMP-activated protein kinase and enhancement of basal glucose uptake in muscle cells by quercetin and quercetin glycosides, active principles of the antidiabetic medicinal plant Vaccinium vitis-idaea. *Mol Nutr Food Res, 54*(7), 991-1003. <https://doi.org/10.1002/mnfr.200900218>

Eidenberger, T., Selg, M., & Krennhuber, K. (2013, Sep). Inhibition of dipeptidyl peptidase activity by flavonol glycosides of guava (Psidium guajava L.): a key to the beneficial effects of guava in type II diabetes mellitus. *Fitoterapia, 89*, 74-79. <https://doi.org/10.1016/j.fitote.2013.05.015>

Eidi, A., Eidi, M., & Esmaeili, E. (2006, Nov). Antidiabetic effect of garlic (Allium sativum L.) in normal and streptozotocin-induced diabetic rats. *Phytomedicine, 13*(9-10), 624-629. <https://doi.org/10.1016/j.phymed.2005.09.010>

El-Houri, R. B., Kotowska, D., Christensen, K. B., Bhattacharya, S., Oksbjerg, N., Wolber, G., Kristiansen, K., & Christensen, L. P. (2015, Jul). Polyacetylenes from carrots (Daucus carota) improve glucose uptake in vitro in adipocytes and myotubes. *Food Funct, 6*(7), 2135-2144. <https://doi.org/10.1039/c5fo00223k>

El-Mekkawy, S., Meselhy, M. R., Nkobole, N., & Lall, N. (2013). Three new α-glucosidase inhibitors from guggul, the oleogum resin of Commiphora wightii. *Nat Prod Res, 27*(2), 146-154. <https://doi.org/10.1080/14786419.2012.662651>

Eliza, J., Daisy, P., Ignacimuthu, S., & Duraipandiyan, V. (2009a, Nov 10). Antidiabetic and antilipidemic effect of eremanthin from Costus speciosus (Koen.)Sm., in STZ-induced diabetic rats. *Chem Biol Interact, 182*(1), 67-72. <https://doi.org/10.1016/j.cbi.2009.08.012>

Eliza, J., Daisy, P., Ignacimuthu, S., & Duraipandiyan, V. (2009b, May 15). Normo-glycemic and hypolipidemic effect of costunolide isolated from Costus speciosus (Koen ex. Retz.)Sm. in streptozotocin-induced diabetic rats. *Chem Biol Interact, 179*(2-3), 329-334. <https://doi.org/10.1016/j.cbi.2008.10.017>

Escandón-Rivera, S., González-Andrade, M., Bye, R., Linares, E., Navarrete, A., & Mata, R. (2012, May 25). α-glucosidase inhibitors from Brickellia cavanillesii. *J Nat Prod, 75*(5), 968-974. <https://doi.org/10.1021/np300204p>

Ezzat, S. M., Bishbishy, M. H. E., Habtemariam, S., Salehi, B., Sharifi-Rad, M., Martins, N., & Sharifi-Rad, J. (2018, Dec 15). Looking at Marine-Derived Bioactive Molecules as Upcoming Anti-Diabetic Agents: A Special Emphasis on PTP1B Inhibitors. *Molecules, 23*(12). <https://doi.org/10.3390/molecules23123334>

Famobuwa, O., Agbowuro, A., Adekunbi, E., & Akinwale, M. J. I. J. B. R. R. (2019). Isolation and characterization of bergapten from the root bark of Ficus exasperata (Vahl). *25*, 1-5.

Fang, X.-K., Gao, J., & Zhu, D.-N. (2008). Kaempferol and quercetin isolated from Euonymus alatus improve glucose uptake of 3T3-L1 cells without adipogenesis activity. *Life sciences, 82*(11-12), 615-622.

Feunaing, R. T., Tamfu, A. N., Ntchapda, F., Gade, I. S., Mbane, M. N., Tagatsing, M. F., Talla, E., Henoumont, C., Laurent, S., & Dinica, R. M. (2021). A new abietane-type diterpenoid from roots of Burkea africana Hook (Fabaceae) with α-amylase inhibitory potential. *Natural Product Research*, 1-8.

Figueirinha, A., Paranhos, A., Pérez-Alonso, J., Santos-Buelga, C., & Batista, M. (2008, 10/01). Cymbopogon citratus leaves: Characterization of flavonoids by HPLC–PDA–ESI/MS/MS and an approach to their potential as a source of bioactive polyphenols. *Food Chemistry - FOOD CHEM, 110*, 718-728. <https://doi.org/10.1016/j.foodchem.2008.02.045>

Folador, P., Cazarolli, L. H., Gazola, A. C., Reginatto, F. H., Schenkel, E. P., & Silva, F. R. (2010, Dec). Potential insulin secretagogue effects of isovitexin and swertisin isolated from Wilbrandia ebracteata roots in non-diabetic rats. *Fitoterapia, 81*(8), 1180-1187. <https://doi.org/10.1016/j.fitote.2010.07.022>

Fuentes, N. L., Sagua, H., Morales, G., Borquez, J., San Martin, A., Soto, J., & Loyola, L. A. (2005, Aug). Experimental antihyperglycemic effect of diterpenoids of llareta Azorella compacta (Umbelliferae) Phil in rats. *Phytother Res, 19*(8), 713-716. <https://doi.org/10.1002/ptr.1740>

Gandhi, G. R., Ignacimuthu, S., Paulraj, M. G., & Sasikumar, P. (2011, Nov 30). Antihyperglycemic activity and antidiabetic effect of methyl caffeate isolated from Solanum torvum Swartz. fruit in streptozotocin induced diabetic rats. *Eur J Pharmacol, 670*(2-3), 623-631. <https://doi.org/10.1016/j.ejphar.2011.09.159>

Gao, D., Li, J., & Liu, Z. J. C. P. (2008). Study of the extraction, purification and antidiabetic potential of ursolic acid from Cornus officinalis Sieb. et Zucc. *5*(5), 697.

Gao, D., Zhang, Y. L., Xu, P., Lin, Y. X., Yang, F. Q., Liu, J. H., Zhu, H. W., & Xia, Z. N. (2015, Mar 13). In vitro evaluation of dual agonists for PPARγ/β from the flower of Edgeworthia gardneri (wall.) Meisn. *J Ethnopharmacol, 162*, 14-19. <https://doi.org/10.1016/j.jep.2014.12.034>

Gao, H., Huang, Y.-N., Xu, P.-Y., & Kawabata, J. J. F. C. (2007). Inhibitory effect on α-glucosidase by the fruits of Terminalia chebula Retz. *Food Chemistry, 105*(2), 628-634.

Gao, H., Huang, Y. N., Gao, B., Li, P., Inagaki, C., & Kawabata, J. (2008, Jun 1). Inhibitory effect on α-glucosidase by Adhatoda vasica Nees. *Food Chem, 108*(3), 965-972. <https://doi.org/10.1016/j.foodchem.2007.12.002>

Gaur, R., Yadav, K. S., Verma, R. K., Yadav, N. P., & Bhakuni, R. S. (2014, Mar 15). In vivo anti-diabetic activity of derivatives of isoliquiritigenin and liquiritigenin. *Phytomedicine, 21*(4), 415-422. <https://doi.org/10.1016/j.phymed.2013.10.015>

Geetha, B., Mathew, B., Augusti, K. J. I. j. o. p., & pharmacology. (1994). Hypoglycemic effects of leucodelphinidin derivative isolated from Ficus bengalensis (Linn.). *Indian journal of physiology and pharmacology, 38*, 220-220.

Genta, S. B., Cabrera, W. M., Mercado, M. I., Grau, A., Catalán, C. A., & Sánchez, S. S. (2010, Apr 29). Hypoglycemic activity of leaf organic extracts from Smallanthus sonchifolius: Constituents of the most active fractions. *Chem Biol Interact, 185*(2), 143-152. <https://doi.org/10.1016/j.cbi.2010.03.004>

Ghorbani, A. J. B., & Pharmacotherapy. (2017). Mechanisms of antidiabetic effects of flavonoid rutin. *96*, 305-312.

Ghosh, S., More, P., Derle, A., Patil, A. B., Markad, P., Asok, A., Kumbhar, N., Shaikh, M. L., Ramanamurthy, B., & Shinde, V. S. J. P. o. (2014). Diosgenin from Dioscorea bulbifera: novel hit for treatment of type II diabetes mellitus with inhibitory activity against α-amylase and α-glucosidase. *9*(9), e106039.

Ghosh, T., Maity, T. K., & Singh, J. (2011, May). Antihyperglycemic activity of bacosine, a triterpene from Bacopa monnieri, in alloxan-induced diabetic rats. *Planta Med, 77*(8), 804-808. <https://doi.org/10.1055/s-0030-1250600>

Govorko, D., Logendra, S., Wang, Y., Esposito, D., Komarnytsky, S., Ribnicky, D., Poulev, A., Wang, Z., Cefalu, W. T., & Raskin, I. (2007, Dec). Polyphenolic compounds from Artemisia dracunculus L. inhibit PEPCK gene expression and gluconeogenesis in an H4IIE hepatoma cell line. *Am J Physiol Endocrinol Metab, 293*(6), E1503-1510. <https://doi.org/10.1152/ajpendo.00420.2007>

Gulfraz, M., Mehmood, S., Ahmad, A., Fatima, N., Praveen, Z., & Williamson, E. J. P. R. (2008). Comparison of the antidiabetic activity of Berberis lyceum root extract and berberine in alloxan‐induced diabetic rats. *22*(9), 1208-1212.

Gunathilaka, T. L., Samarakoon, K., Ranasinghe, P., & Peiris, L. D. C. J. J. o. d. r. (2020). Antidiabetic potential of marine brown algae—A mini review. *2020*.

Guo, J., Zhou, L. Y., He, H. P., Leng, Y., Yang, Z., & Hao, X. J. (2012, Oct 9). Inhibition of 11b-HSD1 by tetracyclic triterpenoids from Euphorbia kansui. *Molecules, 17*(10), 11826-11838. <https://doi.org/10.3390/molecules171011826>

Guo, Z., Niu, X., Xiao, T., Lu, J., Li, W., & Zhao, Y. J. J. o. F. F. (2015). Chemical profile and inhibition of α-glycosidase and protein tyrosine phosphatase 1B (PTP1B) activities by flavonoids from licorice (Glycyrrhiza uralensis Fisch). *Journal of Functional Foods, 14*, 324-336.

Ha do, T., Trung, T. N., Hien, T. T., Dao, T. T., Yim, N., Ngoc, T. M., Oh, W. K., & Bae, K. (2010, Sep 15). Selected compounds derived from Moutan Cortex stimulated glucose uptake and glycogen synthesis via AMPK activation in human HepG2 cells. *J Ethnopharmacol, 131*(2), 417-424. <https://doi.org/10.1016/j.jep.2010.07.010>

Ha do, T., Tuan, D. T., Thu, N. B., Nhiem, N. X., Ngoc, T. M., Yim, N., & Bae, K. (2009, Oct 1). Palbinone and triterpenes from Moutan Cortex (Paeonia suffruticosa, Paeoniaceae) stimulate glucose uptake and glycogen synthesis via activation of AMPK in insulin-resistant human HepG2 Cells. *Bioorg Med Chem Lett, 19*(19), 5556-5559. <https://doi.org/10.1016/j.bmcl.2009.08.048>

Hafizur, R. M., Hameed, A., Shukrana, M., Raza, S. A., Chishti, S., Kabir, N., & Siddiqui, R. A. J. P. (2015). Cinnamic acid exerts anti-diabetic activity by improving glucose tolerance in vivo and by stimulating insulin secretion in vitro. *22*(2), 297-300.

Hamid, H., Yusoff, M., Liu, M., & Karim, M. J. j. o. f. f. (2015). α-Glucosidase and α-amylase inhibitory constituents of Tinospora crispa: Isolation and chemical profile confirmation by ultra-high performance liquid chromatography-quadrupole time-of-flight/mass spectrometry. *Journal of Functional Foods, 16*, 74-80.

Han, Y. M., Oh, H., Na, M., Kim, B. S., Oh, W. K., Kim, B. Y., Jeong, D. G., Ryu, S. E., Sok, D.-E., Ahn, J. S. J. B., & Bulletin, P. (2005). PTP1B inhibitory effect of abietane diterpenes isolated from Salvia miltiorrhiza. *Biological and Pharmaceutical Bulletin, 28*(9), 1795-1797.

Hanchang, W., Khamchan, A., Wongmanee, N., & Seedadee, C. (2019, Oct 15). Hesperidin ameliorates pancreatic β-cell dysfunction and apoptosis in streptozotocin-induced diabetic rat model. *Life Sci, 235*, 116858. <https://doi.org/10.1016/j.lfs.2019.116858>

Hano, C., Renouard, S., Molinié, R., Corbin, C., Barakzoy, E., Doussot, J., Lamblin, F., & Lainé, E. (2013, May 15). Flaxseed (Linum usitatissimum L.) extract as well as (+)-secoisolariciresinol diglucoside and its mammalian derivatives are potent inhibitors of α-amylase activity. *Bioorg Med Chem Lett, 23*(10), 3007-3012. <https://doi.org/10.1016/j.bmcl.2013.03.029>

Harish, M., Ahmed, F., Urooj, A. J. J. o. f. s., & technology. (2014). In vitro hypoglycemic effects of Butea monosperma Lam. leaves and bark. *51*(2), 308-314.

He, Y., Li, W., Li, Y., Zhang, S., Wang, Y., & Sun, C. J. P. o. (2014). Ursolic acid increases glucose uptake through the PI3K signaling pathway in adipocytes. *9*(10), e110711.

Heiss, E. H., Baumgartner, L., Schwaiger, S., Heredia, R. J., Atanasov, A. G., Rollinger, J. M., Stuppner, H., & Dirsch, V. M. (2012, May). Ratanhiaphenol III from Ratanhiae radix is a PTP1B inhibitor. *Planta Med, 78*(7), 678-681. <https://doi.org/10.1055/s-0031-1298242>

Heo, S. J., Hwang, J. Y., Choi, J. I., Han, J. S., Kim, H. J., & Jeon, Y. J. (2009, Aug 1). Diphlorethohydroxycarmalol isolated from Ishige okamurae, a brown algae, a potent alpha-glucosidase and alpha-amylase inhibitor, alleviates postprandial hyperglycemia in diabetic mice. *Eur J Pharmacol, 615*(1-3), 252-256. <https://doi.org/10.1016/j.ejphar.2009.05.017>

Hou, C. C., Lin, S. J., Cheng, J. T., & Hsu, F. L. (2003, May). Antidiabetic dimeric guianolides and a lignan glycoside from Lactuca indica. *J Nat Prod, 66*(5), 625-629. <https://doi.org/10.1021/np0205349>

Hou, W., Li, Y., Zhang, Q., Wei, X., Peng, A., Chen, L., & Wei, Y. (2009, May). Triterpene acids isolated from Lagerstroemia speciosa leaves as alpha-glucosidase inhibitors. *Phytother Res, 23*(5), 614-618. <https://doi.org/10.1002/ptr.2661>

Hu, Y.-C., Zhang, Z., Shi, W.-G., Mi, T.-Y., Zhou, L.-X., Huang, N., Hoptroff, M., Lu, Y.-H. J. J. o. a., & chemistry, f. (2014). 2′, 4′-Dihydroxy-6′-methoxy-3′, 5′-dimethylchalcone promoted glucose uptake and imposed a paradoxical effect on adipocyte differentiation in 3T3-L1 cells. *62*(8), 1898-1904.

Huang, D.-W., Chang, W.-C., Wu, J. S.-B., Shih, R.-W., Shen, S.-C. J. J. o. a., & chemistry, f. (2016). Vescalagin from pink wax apple [Syzygium samarangense (Blume) Merrill and Perry] alleviates hepatic insulin resistance and ameliorates glycemic metabolism abnormality in rats fed a high-fructose diet. *64*(5), 1122-1129.

Huang, D., Jiang, Y., Chen, W., Yao, F., Huang, G., & Sun, L. (2015, Apr 2). Evaluation of hypoglycemic effects of polyphenols and extracts from Penthorum chinense. *J Ethnopharmacol, 163*, 256-263. <https://doi.org/10.1016/j.jep.2015.01.014>

Huang, H. T., Wang, S. L., Nguyen, V. B., & Kuo, Y. H. (2018, Nov 2). Isolation and Identification of Potent Antidiabetic Compounds from Antrodia cinnamomea-An Edible Taiwanese Mushroom. *Molecules, 23*(11). <https://doi.org/10.3390/molecules23112864>

Huseini, H. F., Larijani, B., Heshmat, R., Fakhrzadeh, H., Radjabipour, B., Toliat, T., & Raza, M. (2006, Dec). The efficacy of Silybum marianum (L.) Gaertn. (silymarin) in the treatment of type II diabetes: a randomized, double-blind, placebo-controlled, clinical trial. *Phytother Res, 20*(12), 1036-1039. <https://doi.org/10.1002/ptr.1988>

Hussein, G. M., Matsuda, H., Nakamura, S., Akiyama, T., Tamura, K., & Yoshikawa, M. (2011, Dec 15). Protective and ameliorative effects of maté (Ilex paraguariensis) on metabolic syndrome in TSOD mice. *Phytomedicine, 19*(1), 88-97. <https://doi.org/10.1016/j.phymed.2011.06.036>

Islam, M. N., Jung, H. A., Sohn, H. S., Kim, H. M., & Choi, J. S. J. A. o. p. r. (2013a). Potent α-glucosidase and protein tyrosine phosphatase 1B inhibitors from Artemisia capillaris. *Arch Pharm Res, 36*(5), 542-552.

Islam, M. N., Jung, H. A., Sohn, H. S., Kim, H. M., & Choi, J. S. J. A. o. p. r. (2013b). Potent α-glucosidase and protein tyrosine phosphatase 1B inhibitors from Artemisia capillaris. *Archives of Pharmacal Research 36*(5), 542-552.

Jabeen, B., Riaz, N., Saleem, M., Naveed, M. A., Ashraf, M., Alam, U., Rafiq, H. M., Tareen, R. B., & Jabbar, A. (2013, Dec). Isolation of natural compounds from Phlomis stewartii showing α-glucosidase inhibitory activity. *Phytochemistry, 96*, 443-448. <https://doi.org/10.1016/j.phytochem.2013.09.015>

Jain, V., Viswanatha, G. L., Manohar, D., Shivaprasad, H. J. E.-B. C., & Medicine, A. (2012). Isolation of antidiabetic principle from fruit rinds of Punica granatum. *2012*.

Jaiswal, N., Maurya, C. K., Venkateswarlu, K., Sukanya, P., Srivastava, A. K., Narender, T., & Tamrakar, A. K. (2012, Oct). 4-Hydroxyisoleucine stimulates glucose uptake by increasing surface GLUT4 level in skeletal muscle cells via phosphatidylinositol-3-kinase-dependent pathway. *Eur J Nutr, 51*(7), 893-898. <https://doi.org/10.1007/s00394-012-0374-9>

Jeong, S. Y., Nguyen, P. H., Zhao, B. T., Ali, M. Y., Choi, J. S., Min, B. S., & Woo, M. H. (2015, Oct). Chemical Constituents of Euonymus alatus (Thunb.) Sieb. and Their PTP1B and α-Glucosidase Inhibitory Activities. *Phytother Res, 29*(10), 1540-1548. <https://doi.org/10.1002/ptr.5411>

Jeong, S. Y., Nguyen, P. H., Zhao, B. T., Ali, M. Y., Choi, J. S., Min, B. S., & Woo, M. H. J. P. R. (2015). Chemical Constituents of Euonymus alatus (Thunb.) Sieb. and Their PTP1B and α‐Glucosidase Inhibitory Activities. *Phytotherapy Research, 29*(10), 1540-1548.

Jiang, B., Ji, M., Liu, W., Chen, L., Cai, Z., Zhao, Y., & Bi, X. J. M. m. r. (2016). Antidiabetic activities of a cucurbitane‑type triterpenoid compound from Momordica charantia in alloxan‑induced diabetic mice. *14*(5), 4865-4872.

Jong-Anurakkun, N., Bhandari, M. R., Hong, G., & Kawabata, J. (2008, Sep). Alpha-glucosidase inhibitor from Chinese aloes. *Fitoterapia, 79*(6), 456-457. <https://doi.org/10.1016/j.fitote.2008.02.010>

Jung, H. A., Islam, M. N., Lee, C. M., Oh, S. H., Lee, S., Jung, J. H., & Choi, J. S. (2013, Oct 25). Kinetics and molecular docking studies of an anti-diabetic complication inhibitor fucosterol from edible brown algae Eisenia bicyclis and Ecklonia stolonifera. *Chem Biol Interact, 206*(1), 55-62. <https://doi.org/10.1016/j.cbi.2013.08.013>

Jung, H. A., Yoon, N. Y., Woo, M.-H., & Choi, J. S. J. F. s. (2008). Inhibitory activities of extracts from several kinds of seaweeds and phlorotannins from the brown alga Ecklonia stolonifera on glucose-mediated protein damage and rat lens aldose reductase. *Fisheries science, 74*(6), 1363-1365.

Jung, H. J., Jung, H. A., Kang, S. S., Lee, J. H., Cho, Y. S., Moon, K. H., & Choi, J. S. (2012, Oct). Inhibitory activity of Aralia continentalis roots on protein tyrosine phosphatase 1B and rat lens aldose reductase. *Arch Pharm Res, 35*(10), 1771-1777. <https://doi.org/10.1007/s12272-012-1009-7>

Jung, S. H., Seol, H. J., Jeon, S. J., Son, K. H., & Lee, J. R. (2009, Apr). Insulin-sensitizing activities of tanshinones, diterpene compounds of the root of Salvia miltiorrhiza Bunge. *Phytomedicine, 16*(4), 327-335. <https://doi.org/10.1016/j.phymed.2008.12.017>

Jung, U. J., Lee, M.-K., Park, Y. B., Jeon, S.-M., Choi, M.-S. J. J. o. p., & therapeutics, e. (2006). Antihyperglycemic and antioxidant properties of caffeic acid in db/db mice. *318*(2), 476-483.

Kang, M. J., Kim, J. I., Yoon, S. Y., Kim, J. C., & Cha, I. J. (2006, Summer). Pinitol from soybeans reduces postprandial blood glucose in patients with type 2 diabetes mellitus. *J Med Food, 9*(2), 182-186. <https://doi.org/10.1089/jmf.2006.9.182>

Karan, S. K., Mondal, A., Mishra, S. K., Pal, D., & Rout, K. K. (2013, Mar). Antidiabetic effect of Streblus asper in streptozotocin-induced diabetic rats. *Pharm Biol, 51*(3), 369-375. <https://doi.org/10.3109/13880209.2012.730531>

Kato, E., Kushibiki, N., Satoh, H., & Kawabata, J. J. N. p. r. (2020). Silychristin derivatives conjugated with coniferylalcohols from silymarin and their pancreatic α-amylase inhibitory activity. *34*(6), 759-765.

Katsube, T., Yamasaki, M., Shiwaku, K., Ishijima, T., Matsumoto, I., Abe, K., & Yamasaki, Y. (2010, Nov). Effect of flavonol glycoside in mulberry (Morus alba L.) leaf on glucose metabolism and oxidative stress in liver in diet-induced obese mice. *J Sci Food Agric, 90*(14), 2386-2392. <https://doi.org/10.1002/jsfa.4096>

Kawano, A., Nakamura, H., Hata, S., Minakawa, M., Miura, Y., & Yagasaki, K. (2009, May). Hypoglycemic effect of aspalathin, a rooibos tea component from Aspalathus linearis, in type 2 diabetic model db/db mice. *Phytomedicine, 16*(5), 437-443. <https://doi.org/10.1016/j.phymed.2008.11.009>

Keller, A. C., Ma, J., Kavalier, A., He, K., Brillantes, A. M., & Kennelly, E. J. (2011, Dec 15). Saponins from the traditional medicinal plant Momordica charantia stimulate insulin secretion in vitro. *Phytomedicine, 19*(1), 32-37. <https://doi.org/10.1016/j.phymed.2011.06.019>

Khan, M. F., Dixit, P., Jaiswal, N., Tamrakar, A. K., Srivastava, A. K., & Maurya, R. (2012, Jan). Chemical constituents of Kigelia pinnata twigs and their GLUT4 translocation modulatory effect in skeletal muscle cells. *Fitoterapia, 83*(1), 125-129. <https://doi.org/10.1016/j.fitote.2011.10.002>

Khathi, A., Serumula, M. R., Myburg, R. B., Van Heerden, F. R., & Musabayane, C. T. (2013). Effects of Syzygium aromaticum-derived triterpenes on postprandial blood glucose in streptozotocin-induced diabetic rats following carbohydrate challenge. *PloS one, 8*(11), e81632. <https://doi.org/10.1371/journal.pone.0081632>

Kim, K. Y., Nam, K. A., Kurihara, H., & Kim, S. M. (2008, Nov). Potent alpha-glucosidase inhibitors purified from the red alga Grateloupia elliptica. *Phytochemistry, 69*(16), 2820-2825. <https://doi.org/10.1016/j.phytochem.2008.09.007>

Kim, M. B., Kim, C., Song, Y., & Hwang, J. K. (2014). Antihyperglycemic and Anti-Inflammatory Effects of Standardized Curcuma xanthorrhiza Roxb. Extract and Its Active Compound Xanthorrhizol in High-Fat Diet-Induced Obese Mice. *Evid Based Complement Alternat Med, 2014*, 205915. <https://doi.org/10.1155/2014/205915>

Kim, M. J., Kim, H. J., & Han, J. S. J. P. R. (2019). Pheophorbide A from Gelidium amansii improves postprandial hyperglycemia in diabetic mice through α‐glucosidase inhibition. *33*(3), 702-707.

Kim, Y. C., Oh, H., Kim, B. S., Kang, T. H., Ko, E. K., Han, Y. M., Kim, B. Y., & Ahn, J. S. (2005, Jan). In vitro protein tyrosine phosphatase 1B inhibitory phenols from the seeds of Psoralea corylifolia. *Planta Med, 71*(1), 87-89. <https://doi.org/10.1055/s-2005-837759>

Krishnan, S. S. C., Subramanian, I. P., Subramanian, S. P. J. B., & Nutrition, P. (2014). Isolation, characterization of syringin, phenylpropanoid glycoside from Musa paradisiaca tepal extract and evaluation of its antidiabetic effect in streptozotocin-induced diabetic rats. *Biomedicine & Preventive Nutrition, 4*(2), 105-111.

Kuhad, A., & Chopra, K. (2007, Dec 8). Curcumin attenuates diabetic encephalopathy in rats: behavioral and biochemical evidences. *Eur J Pharmacol, 576*(1-3), 34-42. <https://doi.org/10.1016/j.ejphar.2007.08.001>

Kumar, D., Datta, S., Roy, S. S., Gaonkar, R. H., Vedasiromoni, J., Ghosh, R., Pal, B. C. J. J. o. h., spices, & plants, m. (2013). Bioactivity-guided isolation and quantification of anti-diabetic principle in vitro from Holarrhena antidysenterica L.(Wall). *Journal of herbs, spices & medicinal plants, 19*(1), 54-65.

Kumar, D., Ghosh, R., & Pal, B. C. J. J. o. F. F. (2013). α-Glucosidase inhibitory terpenoids from Potentilla fulgens and their quantitative estimation by validated HPLC method. *Journal of Functional Foods, 5*(3), 1135-1141.

Kumar, M. P., Sankeshi, V., Naik, R. R., Thirupathi, P., Das, B., & Raju, T. N. (2015, Jul 25). The inhibitory effect of Isoflavones isolated from Caesalpinia pulcherrima on aldose reductase in STZ induced diabetic rats. *Chem Biol Interact, 237*, 18-24. <https://doi.org/10.1016/j.cbi.2015.05.010>

Kumar, R., Patel, D. K., Prasad, S. K., Laloo, D., Krishnamurthy, S., & Hemalatha, S. (2012, Mar). Type 2 antidiabetic activity of bergenin from the roots of Caesalpinia digyna Rottler. *Fitoterapia, 83*(2), 395-401. <https://doi.org/10.1016/j.fitote.2011.12.008>

Kumar, S., Kumar, V., & Prakash, O. (2013). Enzymes inhibition and antidiabetic effect of isolated constituents from Dillenia indica. *Biomed Res Int, 2013*, 382063. <https://doi.org/10.1155/2013/382063>

Kumari, K., & Augusti, K. (2002). Antidiabetic and antioxidant effects of S-methyl cysteine sulfoxide isolated from onions (Allium cepa Linn) as compared to standard drugs in alloxan diabetic rats.

Kuroda, M., Mimaki, Y., Ohtomo, T., Yamada, J., Nishiyama, T., Mae, T., Kishida, H., & Kawada, T. (2012, Apr). Hypoglycemic effects of clove (Syzygium aromaticum flower buds) on genetically diabetic KK-Ay mice and identification of the active ingredients. *J Nat Med, 66*(2), 394-399. <https://doi.org/10.1007/s11418-011-0593-z>

Kwon, E.-B., Kang, M.-J., Ryu, H. W., Lee, S., Lee, J.-W., Lee, M. K., Lee, H.-S., Lee, S. U., Oh, S.-R., & Kim, M.-O. J. P. (2020). Acacetin enhances glucose uptake through insulin-independent GLUT4 translocation in L6 myotubes. *68*, 153178.

Lai, W. C., Wu, Y. C., Dankó, B., Cheng, Y. B., Hsieh, T. J., Hsieh, C. T., Tsai, Y. C., El-Shazly, M., Martins, A., Hohmann, J., Hunyadi, A., & Chang, F. R. (2014, Jul 25). Bioactive constituents of Cirsium japonicum var. australe. *J Nat Prod, 77*(7), 1624-1631. <https://doi.org/10.1021/np500233t>

Lam, S.-H., Chen, J.-M., Kang, C.-J., Chen, C.-H., & Lee, S.-S. J. P. (2008). α-Glucosidase inhibitors from the seeds of Syagrus romanzoffiana. *Phytochemistry, 69*(5), 1173-1178.

Lam, S. H., Ruan, C. T., Hsieh, P. H., Su, M. J., & Lee, S. S. (2012, Feb 24). Hypoglycemic diterpenoids from Tinospora crispa. *J Nat Prod, 75*(2), 153-159. <https://doi.org/10.1021/np200692v>

Latha, R. C., & Daisy, P. (2011, Jan 15). Insulin-secretagogue, antihyperlipidemic and other protective effects of gallic acid isolated from Terminalia bellerica Roxb. in streptozotocin-induced diabetic rats. *Chem Biol Interact, 189*(1-2), 112-118. <https://doi.org/10.1016/j.cbi.2010.11.005>

Lee, H.-S. J. J. o. a., & chemistry, f. (2005). Cuminaldehyde: aldose reductase and α-glucosidase inhibitor derived from Cuminum cyminum L. seeds. *Journal of agricultural and food chemistry, 53*(7), 2446-2450.

Lee, J. S. (2006, Sep 13). Effects of soy protein and genistein on blood glucose, antioxidant enzyme activities, and lipid profile in streptozotocin-induced diabetic rats. *Life Sci, 79*(16), 1578-1584. <https://doi.org/10.1016/j.lfs.2006.06.030>

Lee, J. Y., Kim, S. M., Jung, W.-S., Song, D.-G., Um, B.-H., Son, J.-K., & Pan, C.-H. (2012a, 2012/12/01). Phlorofucofuroeckol-A, a potent inhibitor of aldo-keto reductase family 1 member B10, from the edible brown alga Eisenia bicyclis. *Journal of the Korean Society for Applied Biological Chemistry, 55*(6), 721-727. <https://doi.org/10.1007/s13765-012-2169-3>

Lee, J. Y., Kim, S. M., Jung, W.-S., Song, D.-G., Um, B.-H., Son, J.-K., & Pan, C.-H. J. J. o. t. K. S. f. A. B. C. (2012b). Phlorofucofuroeckol-A, a potent inhibitor of aldo-keto reductase family 1 member B10, from the edible brown alga Eisenia bicyclis. *Journal of the Korean Society for Applied Biological Chemistry, 55*(6), 721-727.

Lee, M. S., Hwang, J. T., Kim, S. H., Yoon, S., Kim, M. S., Yang, H. J., & Kwon, D. Y. (2010, Feb 17). Ginsenoside Rc, an active component of Panax ginseng, stimulates glucose uptake in C2C12 myotubes through an AMPK-dependent mechanism. *J Ethnopharmacol, 127*(3), 771-776. <https://doi.org/10.1016/j.jep.2009.11.022>

Lee, M. S., Kim, C. H., Hoang, D. M., Kim, B. Y., Sohn, C. B., Kim, M. R., & Ahn, J. S. (2009, Mar). Genistein-derivatives from Tetracera scandens stimulate glucose-uptake in L6 myotubes. *Biol Pharm Bull, 32*(3), 504-508. <https://doi.org/10.1248/bpb.32.504>

Lee, M. S., & Sohn, C. B. (2008, Nov). Anti-diabetic properties of chrysophanol and its glucoside from rhubarb rhizome. *Biol Pharm Bull, 31*(11), 2154-2157. <https://doi.org/10.1248/bpb.31.2154>

Lee, S. H., & Jeon, Y. J. (2013, Apr). Anti-diabetic effects of brown algae derived phlorotannins, marine polyphenols through diverse mechanisms. *Fitoterapia, 86*, 129-136. <https://doi.org/10.1016/j.fitote.2013.02.013>

Lee, S. H., Karadeniz, F., Kim, M. M., Kim, S. K. J. J. o. t. S. o. F., & Agriculture. (2009). α‐Glucosidase and α‐amylase inhibitory activities of phloroglucinal derivatives from edible marine brown alga, Ecklonia cava. *89*(9), 1552-1558.

Lee, S. W., Rho, M. C., Park, H. R., Choi, J. H., Kang, J. Y., Lee, J. W., Kim, K., Lee, H. S., & Kim, Y. K. (2006, Dec 27). Inhibition of diacylglycerol acyltransferase by alkamides isolated from the fruits of Piper longum and Piper nigrum. *J Agric Food Chem, 54*(26), 9759-9763. <https://doi.org/10.1021/jf061402e>

Li, J. L., Gao, L. X., Meng, F. W., Tang, C. L., Zhang, R. J., Li, J. Y., Luo, C., Li, J., & Zhao, W. M. (2015). PTP1B inhibitors from stems of Angelica keiskei (Ashitaba). *Bioorg Med Chem Lett, 25*(10), 2028-2032. <https://doi.org/10.1016/j.bmcl.2015.04.003>

Li, Q., Zhang, X., Cao, J., Guo, Z., Lou, Y., Ding, M., & Zhao, Y. (2015, Sep). Depside derivatives with anti-hepatic fibrosis and anti-diabetic activities from Impatiens balsamina L. flowers. *Fitoterapia, 105*, 234-239. <https://doi.org/10.1016/j.fitote.2015.07.007>

Li, S., Li, J., Guan, X. L., Li, J., Deng, S. P., Li, L. Q., Tang, M. T., Huang, J. G., Chen, Z. Z., & Yang, R. Y. (2011, Oct). Hypoglycemic effects and constituents of the barks of Cyclocarya paliurus and their inhibiting activities to glucosidase and glycogen phosphorylase. *Fitoterapia, 82*(7), 1081-1085. <https://doi.org/10.1016/j.fitote.2011.07.002>

Li, S., Li, W., Wang, Y., Asada, Y., & Koike, K. (2010, Sep 15). Prenylflavonoids from Glycyrrhiza uralensis and their protein tyrosine phosphatase-1B inhibitory activities. *Bioorg Med Chem Lett, 20*(18), 5398-5401. <https://doi.org/10.1016/j.bmcl.2010.07.110>

Li, Y., Zhang, Y., Shen, X., & Guo, Y. W. (2009, Jan 15). A novel sesquiterpene quinone from Hainan sponge Dysidea villosa. *Bioorg Med Chem Lett, 19*(2), 390-392. <https://doi.org/10.1016/j.bmcl.2008.11.068>

Lin, M. H., Liu, H. K., Huang, W. J., Huang, C. C., Wu, T. H., & Hsu, F. L. (2011, Jul 27). Evaluation of the potential hypoglycemic and Beta-cell protective constituents isolated from Corni fructus to tackle insulin-dependent diabetes mellitus. *J Agric Food Chem, 59*(14), 7743-7751. <https://doi.org/10.1021/jf201189r>

Liu, Q., Yang, Q. M., Hu, H. J., Yang, L., Yang, Y. B., Chou, G. X., & Wang, Z. T. (2014, Jul 25). Bioactive diterpenoids and flavonoids from the aerial parts of Scoparia dulcis. *J Nat Prod, 77*(7), 1594-1600. <https://doi.org/10.1021/np500150f>

Liu, Z., Li, W., Li, X., Zhang, M., Chen, L., Zheng, Y. N., Sun, G. Z., & Ruan, C. C. (2013, Jan 9). Antidiabetic effects of malonyl ginsenosides from Panax ginseng on type 2 diabetic rats induced by high-fat diet and streptozotocin. *J Ethnopharmacol, 145*(1), 233-240. <https://doi.org/10.1016/j.jep.2012.10.058>

Liyanagamage, D., Jayasinghe, S., Attanayake, A., Karunaratne, V., & Wijesundara, D. J. C. J. o. S. (2020). Antihyperglycemic activity of fruit extracts of Sri Lankan endemic species Garcinia quaesita Pierre “Rathgoraka” and its isolated compound, garcinol. *Ceylon Journal of Science, 49*(3), 303-309.

Luo, L., Wang, R., Wang, X., Ma, Z., & Li, N. J. F. C. (2012). Compounds from Angelica keiskei with NQO1 induction, DPPH scavenging and α-glucosidase inhibitory activities. *Food Chem, 131*(3), 992-998.

Luyen, B. T., Tai, B. H., Thao, N. P., Yang, S. Y., Cuong, N. M., Kwon, Y. I., Jang, H. D., & Kim, Y. H. (2014, Sep 1). A new phenylpropanoid and an alkylglycoside from Piper retrofractum leaves with their antioxidant and α-glucosidase inhibitory activity. *Bioorg Med Chem Lett, 24*(17), 4120-4124. <https://doi.org/10.1016/j.bmcl.2014.07.057>

Ma, Y. Y., Zhao, D. G., Zhou, A. Y., Zhang, Y., Du, Z., & Zhang, K. (2015, Sep 23). α-Glucosidase Inhibition and Antihyperglycemic Activity of Phenolics from the Flowers of Edgeworthia gardneri. *J Agric Food Chem, 63*(37), 8162-8169. <https://doi.org/10.1021/acs.jafc.5b03081>

Mai, D. T., Le, T. D., Nguyen, T. P., Phan, N. M., Nguyen, H. A., Nguyen, T. T., & Tran le, Q. (2015). A new aldehyde compound from the fruit of Pandanus tectorius Parkinson ex Du Roi. *Nat Prod Res, 29*(15), 1437-1441. <https://doi.org/10.1080/14786419.2014.1003929>

Makheswari, M. U., & Sudarsanam, D. J. I. J. P. S. R. (2012). Database on antidiabetic indigenous plants of Tamil Nadhu, India. *Int J Pharma Sci Res, 3*(2), 287-293.

Manaharan, T., Ming, C. H., & Palanisamy, U. D. (2013, Jan 15). Syzygium aqueum leaf extract and its bioactive compounds enhances pre-adipocyte differentiation and 2-NBDG uptake in 3T3-L1 cells. *Food Chem, 136*(2), 354-363. <https://doi.org/10.1016/j.foodchem.2012.08.056>

Mandal, B., & Maity, C. J. A. p. e. p. B. (1986). Hypoglycemic action of karanjin. *12*(4), 42-46.

Matsuda, H., Asao, Y., Nakamura, S., Hamao, M., Sugimoto, S., Hongo, M., Pongpiriyadacha, Y., & Yoshikawa, M. (2009, May). Antidiabetogenic constituents from the Thai traditional medicine Cotylelobium melanoxylon. *Chem Pharm Bull (Tokyo), 57*(5), 487-494. <https://doi.org/10.1248/cpb.57.487>

Maurya, R., Akanksha, Jayendra, Singh, A. B., & Srivastava, A. K. (2008, Dec 15). Coagulanolide, a withanolide from Withania coagulans fruits and antihyperglycemic activity. *Bioorg Med Chem Lett, 18*(24), 6534-6537. <https://doi.org/10.1016/j.bmcl.2008.10.050>

Merghache, S., Zerriouh, M., Merghache, D., Boufeldja, T., Rabah, D., & Said, G. (2013, 04/01). Evaluation of hypoglycaemic and hypolipidemic activities of Globularin isolated from Globularia alypum L. In normal and streptozotocin-induced diabetic rats. *Journal of Applied Pharmaceutical Science, 03*, 001-007. <https://doi.org/10.7324/JAPS.2013.3401>

Mitra, A., & Mahadevappa, M. J. I. J. o. P. (2010). Antidiabetic and hypolipidemic effects of mahanimbine (carbazole alkaloid) from Murraya koenigii (rutaceae) leaves. *International journal of phytomedicine, 2*, 22-30.

Miura, T., Ichiki, H., Hashimoto, I., Iwamoto, N., Kao, M., Kubo, M., Ishihara, E., Komatsu, Y., Okada, M., & Ishida, T. (2001). Antidiabetic activity of a xanthone compound, mangiferin. *Phytomedicine, 8*(2), 85-87.

Mnonopi, N., Levendal, R. A., Mzilikazi, N., & Frost, C. L. (2012, Apr 15). Marrubiin, a constituent of Leonotis leonurus, alleviates diabetic symptoms. *Phytomedicine, 19*(6), 488-493. <https://doi.org/10.1016/j.phymed.2011.12.008>

Mojica, L., Berhow, M., & de Mejia, E. G. J. F. c. (2017). Black bean anthocyanin-rich extracts as food colorants: Physicochemical stability and antidiabetes potential. *229*, 628-639.

Morikawa, T., Akaki, J., Ninomiya, K., Kinouchi, E., Tanabe, G., Pongpiriyadacha, Y., Yoshikawa, M., & Muraoka, O. (2015, Feb 27). Salacinol and related analogs: new leads for type 2 diabetes therapeutic candidates from the Thai traditional natural medicine Salacia chinensis. *Nutrients, 7*(3), 1480-1493. <https://doi.org/10.3390/nu7031480>

Morimitsu, Y., Kubota, K., Tashiro, T., Hashizume, E., Kamiya, T., & Osawa, T. (2002). Inhibitory effect of anthocyanins and colored rice on diabetic cataract formation in the rat lenses. International Congress Series,

Mosihuzzman, M., Naheed, S., Hareem, S., Talib, S., Abbas, G., Khan, S. N., Choudhary, M. I., Sener, B., Tareen, R. B., & Israr, M. (2013, Feb 27). Studies on α-glucosidase inhibition and anti-glycation potential of Iris loczyi and Iris unguicularis. *Life Sci, 92*(3), 187-192. <https://doi.org/10.1016/j.lfs.2012.11.022>

Na, B., Nguyen, P.-H., Zhao, B.-T., Vo, Q.-H., Min, B. S., & Woo, M. H. (2016). Protein tyrosine phosphatase 1B (PTP1B) inhibitory activity and glucosidase inhibitory activity of compounds isolated from Agrimonia pilosa. *Pharmaceutical biology, 54*(3), 474-480.

Na, B., Nguyen, P. H., Zhao, B. T., Vo, Q. H., Min, B. S., & Woo, M. H. (2016). Protein tyrosine phosphatase 1B (PTP1B) inhibitory activity and glucosidase inhibitory activity of compounds isolated from Agrimonia pilosa. *Pharm Biol, 54*(3), 474-480. <https://doi.org/10.3109/13880209.2015.1048372>

Na, M., Jang, J., Njamen, D., Mbafor, J. T., Fomum, Z. T., Kim, B. Y., Oh, W. K., & Ahn, J. S. (2006, Nov). Protein tyrosine phosphatase-1B inhibitory activity of isoprenylated flavonoids isolated from Erythrina mildbraedii. *J Nat Prod, 69*(11), 1572-1576. <https://doi.org/10.1021/np0601861>

Naik, S. R., Barbosa Filho, J. M., Dhuley, J. N., & Deshmukh, V. (1991, May-Jun). Probable mechanism of hypoglycemic activity of bassic acid, a natural product isolated from Bumelia sartorum. *J Ethnopharmacol, 33*(1-2), 37-44. <https://doi.org/10.1016/0378-8741(91)90158-a>

Naik, S. R., Niture, N. T., Ansari, A. A., & Shah, P. D. J. P. (2013). Anti-diabetic activity of embelin: involvement of cellular inflammatory mediators, oxidative stress and other biomarkers. *20*(10), 797-804.

Nair, S. A., Sabulal, B., Radhika, J., Arunkumar, R., & Subramoniam, A. (2014, Jul 5). Promising anti-diabetes mellitus activity in rats of β-amyrin palmitate isolated from Hemidesmus indicus roots. *Eur J Pharmacol, 734*, 77-82. <https://doi.org/10.1016/j.ejphar.2014.03.050>

Nakao, Y., Uehara, T., Matsunaga, S., Fusetani, N., & van Soest, R. W. (2002, Jun). Callyspongynic acid, a polyacetylenic acid which inhibits alpha-glucosidase, from the marine sponge Callyspongia truncata. *J Nat Prod, 65*(6), 922-924. <https://doi.org/10.1021/np0106642>

Narasimhan, A., Chinnaiyan, M., Karundevi, B. J. A. p., nutrition,, & metabolism. (2015). Ferulic acid exerts its antidiabetic effect by modulating insulin-signalling molecules in the liver of high-fat diet and fructose-induced type-2 diabetic adult male rat. *40*(8), 769-781.

Narender, T., Shweta, S., Tiwari, P., Papi Reddy, K., Khaliq, T., Prathipati, P., Puri, A., Srivastava, A. K., Chander, R., Agarwal, S. C., & Raj, K. (2007, Mar 15). Antihyperglycemic and antidyslipidemic agent from Aegle marmelos. *Bioorg Med Chem Lett, 17*(6), 1808-1811. <https://doi.org/10.1016/j.bmcl.2006.12.037>

Naresh, G., Jaiswal, N., Sukanya, P., Srivastava, A., Tamrakar, A., Narender, T. J. B., & letters, m. c. (2012). Glucose uptake stimulatory effect of 4-hydroxypipecolic acid by increased GLUT 4 translocation in skeletal muscle cells. *22*(17), 5648-5651.

Narvaez-Mastache, J. M., Garduño-Ramírez, M. L., Alvarez, L., & Delgado, G. (2006, Dec). Antihyperglycemic activity and chemical constituents of Eysenhardtia platycarpa. *J Nat Prod, 69*(12), 1687-1691. <https://doi.org/10.1021/np060166z>

Nerurkar, P. V., Hwang, P. W., & Saksa, E. J. M. (2015). Anti-diabetic potential of noni: The Yin and the Yang. *20*(10), 17684-17719.

Nguyen, K. H., Ta, T. N., Pham, T. H., Nguyen, Q. T., Pham, H. D., Mishra, S., & Nyomba, B. L. (2012, Jul 13). Nuciferine stimulates insulin secretion from beta cells-an in vitro comparison with glibenclamide. *J Ethnopharmacol, 142*(2), 488-495. <https://doi.org/10.1016/j.jep.2012.05.024>

Nguyen, P.-H., Ji, D.-J., Han, Y.-R., Choi, J.-S., Rhyu, D.-Y., Min, B.-S., & Woo, M.-H. (2015). Selaginellin and biflavonoids as protein tyrosine phosphatase 1B inhibitors from Selaginella tamariscina and their glucose uptake stimulatory effects. *Bioorganic medicinal chemistry, 23*(13), 3730-3737.

Nguyen, P. H., Ji, D. J., Han, Y. R., Choi, J. S., Rhyu, D. Y., Min, B. S., & Woo, M. H. (2015, Jul 1). Selaginellin and biflavonoids as protein tyrosine phosphatase 1B inhibitors from Selaginella tamariscina and their glucose uptake stimulatory effects. *Bioorg Med Chem, 23*(13), 3730-3737. <https://doi.org/10.1016/j.bmc.2015.04.007>

Nguyen, P. H., Nguyen, T. N., Dao, T. T., Kang, H. W., Ndinteh, D. T., Mbafor, J. T., & Oh, W. K. (2010, Apr 23). AMP-activated protein kinase (AMPK) activation by benzofurans and coumestans isolated from Erythrina abyssinica. *J Nat Prod, 73*(4), 598-602. <https://doi.org/10.1021/np900745g>

Nguyen, P. H., Zhao, B. T., Ali, M. Y., Choi, J. S., Rhyu, D. Y., Min, B. S., & Woo, M. H. (2015, Jan 23). Insulin-mimetic selaginellins from Selaginella tamariscina with protein tyrosine phosphatase 1B (PTP1B) inhibitory activity. *J Nat Prod, 78*(1), 34-42. <https://doi.org/10.1021/np5005856>

Nguyen, V. B., Wang, S. L., Nhan, N. T., Nguyen, T. H., Nguyen, N. P. D., Nghi, D. H., & Cuong, N. M. (2018, Jun 29). New Records of Potent In-Vitro Antidiabetic Properties of Dalbergia tonkinensis Heartwood and the Bioactivity-Guided Isolation of Active Compounds. *Molecules, 23*(7). <https://doi.org/10.3390/molecules23071589>

Nhiem, N. X., Van Kiem, P., Van Minh, C., Ban, N. K., Cuong, N. X., Tung, N. H., Le, M. H., Do, T. H., Tai, B. H., Quang, T. H. J. C., & Bulletin, P. (2010). α-Glucosidase inhibition properties of cucurbitane-type triterpene glycosides from the fruits of Momordica charantia. *Chemical and Pharmaceutical Bulletin, 58*(5), 720-724.

Nile, S. H., & Park, S. W. (2014, Jan). Antioxidant, α-glucosidase and xanthine oxidase inhibitory activity of bioactive compounds from maize (Zea mays L.). *Chem Biol Drug Des, 83*(1), 119-125. <https://doi.org/10.1111/cbdd.12205>

Noipha, K., Thongthoom, T., Songsiang, U., Boonyarat, C., & Yenjai, C. (2010, Dec). Carbazoles and coumarins from Clausena harmandiana stimulate glucose uptake in L6 myotubes. *Diabetes Res Clin Pract, 90*(3), e67-71. <https://doi.org/10.1016/j.diabres.2010.09.005>

Nojima, H., Kimura, I., Chen, F. J., Sugihara, Y., Haruno, M., Kato, A., & Asano, N. (1998, Mar). Antihyperglycemic effects of N-containing sugars from Xanthocercis zambesiaca, Morus bombycis, Aglaonema treubii, and Castanospermum australe in streptozotocin-diabetic mice. *J Nat Prod, 61*(3), 397-400. <https://doi.org/10.1021/np970277l>

NoorShahida, A., Wong, T. W., & Choo, C. Y. (2009, Jul 30). Hypoglycemic effect of quassinoids from Brucea javanica (L.) Merr (Simaroubaceae) seeds. *J Ethnopharmacol, 124*(3), 586-591. <https://doi.org/10.1016/j.jep.2009.04.058>

Nurul Islam, M., Jung, H. A., Sohn, H. S., Kim, H. M., & Choi, J. S. (2013, May). Potent α-glucosidase and protein tyrosine phosphatase 1B inhibitors from Artemisia capillaris. *Arch Pharm Res, 36*(5), 542-552. <https://doi.org/10.1007/s12272-013-0069-7>

Ogawa, A., Miyamae, Y., Honma, A., Koyama, T., Yazawa, K., & Shigemori, H. (2011). Pycnalin, a new α-glucosidase inhibitor from Acer pycnanthum. *Chem Pharm Bull (Tokyo), 59*(5), 672-675. <https://doi.org/10.1248/cpb.59.672>

Oh, W. K., Lee, C., Seo, J. H., Chung, M. Y., Cui, L., Fomum, Z. T., Kang, J. S., & Lee, H. S. (2009, Jan). Diacylglycerol acyltransferase-inhibitory compounds from Erythrina senegalensis. *Arch Pharm Res, 32*(1), 43-47. <https://doi.org/10.1007/s12272-009-1116-2>

Ohnishi, M., Matuo, T., Tsuno, T., Hosoda, A., Nomura, E., Taniguchi, H., Sasaki, H., & Morishita, H. J. B. (2004). Antioxidant activity and hypoglycemic effect of ferulic acid in STZ-induced diabetic mice and KK-A^{y} mice. *21*(1-4), 315-319.

Ohta, M., Fujinami, A., Kobayashi, N., Amano, A., Ishigami, A., Tokuda, H., Suzuki, N., Ito, F., Mori, T., Sawada, M., Iwasa, K., Kitawaki, J., Ohnishi, K., Tsujikawa, M., & Obayashi, H. (2015, Jul). Two chalcones, 4-hydroxyderricin and xanthoangelol, stimulate GLUT4-dependent glucose uptake through the LKB1/AMP-activated protein kinase signaling pathway in 3T3-L1 adipocytes. *Nutr Res, 35*(7), 618-625. <https://doi.org/10.1016/j.nutres.2015.05.010>

Omar, R., Li, L., Yuan, T., & Seeram, N. P. (2012, Aug 24). α-Glucosidase inhibitory hydrolyzable tannins from Eugenia jambolana seeds. *J Nat Prod, 75*(8), 1505-1509. <https://doi.org/10.1021/np300417q>

Orhan, N., Aslan, M., Pekcan, M., Orhan, D. D., Bedir, E., & Ergun, F. (2012, Jan 6). Identification of hypoglycaemic compounds from berries of Juniperus oxycedrus subsp. oxycedrus through bioactivity guided isolation technique. *J Ethnopharmacol, 139*(1), 110-118. <https://doi.org/10.1016/j.jep.2011.10.027>

Osigwe, C. C., Akah, P. A., Nworu, C. S., & Okoye, F. B. (2017). Apigenin: A methanol fraction component of Newbouldia laevis leaf, as a potential antidiabetic agent. *Journal of Phytopharmacology, 6*, 38-44.

Park, H. G., Bak, E. J., Woo, G. H., Kim, J. M., Quan, Z., Kim, J. M., Yoon, H. K., Cheon, S. H., Yoon, G., Yoo, Y. J., Na, Y., & Cha, J. H. (2012, Jul). Licochalcone E has an antidiabetic effect. *J Nutr Biochem, 23*(7), 759-767. <https://doi.org/10.1016/j.jnutbio.2011.03.021>

Park, J.-H., Baek, M.-R., Lee, B.-H., Yon, G.-H., Ryu, S.-Y., Kim, Y.-S., Park, S.-U., & Hong, K.-S. J. K. J. o. M. C. S. (2009). $\alpha $-Glucosidase and $\alpha $-Amylase Inhibitory Activity of Compounds from Roots Extract of Pueraria thunbergiana. *17*(5), 357-362.

Park, J.-H., & Lee, H.-S. J. J. o. A. B. C. (2015). Inhibitory effects of quinoline isolated from Ruta chalepensis and its structurally related derivatives against α-amylase or α-glucosidase. *Journal of Applied Biological Chemistry 58*(1), 5-8.

Park, S., Ahn, I. S., Kim, J. H., Lee, M. R., Kim, J. S., & Kim, H. J. (2010, Feb 10). Glyceollins, one of the phytoalexins derived from soybeans under fungal stress, enhance insulin sensitivity and exert insulinotropic actions. *J Agric Food Chem, 58*(3), 1551-1557. <https://doi.org/10.1021/jf903432b>

Patel, M. B., & Mishra, S. M. J. J. o. F. F. (2012). Magnoflorine from Tinospora cordifolia stem inhibits α-glucosidase and is antiglycemic in rats. *Journal of Functional Foods, 4*(1), 79-86.

Patil, S. B., Takalikar, S. S., Joglekar, M. M., Haldavnekar, V. S., & Arvindekar, A. U. (2013, Oct). Insulinotropic and β-cell protective action of cuminaldehyde, cuminol and an inhibitor isolated from Cuminum cyminum in streptozotocin-induced diabetic rats. *Br J Nutr, 110*(8), 1434-1443. <https://doi.org/10.1017/s0007114513000627>

Peng, J., Yuan, J. P., Wu, C. F., & Wang, J. H. (2011). Fucoxanthin, a marine carotenoid present in brown seaweeds and diatoms: metabolism and bioactivities relevant to human health. *Mar Drugs, 9*(10), 1806-1828. <https://doi.org/10.3390/md9101806>

Pereira, A. C., Arruda, M. S., da Silva, E. A., da Silva, M. N., Lemos, V. S., & Cortes, S. F. (2012). Inhibition of α-glucosidase and hypoglycemic effect of stilbenes from the Amazonian plant Deguelia rufescens var. urucu (Ducke) AMG Azevedo (Leguminosae). *Planta medica, 78*(01), 36-38.

Peungvicha, P., Temsiririrkkul, R., Prasain, J. K., Tezuka, Y., Kadota, S., Thirawarapan, S. S., & Watanabe, H. (1998, Aug). 4-Hydroxybenzoic acid: a hypoglycemic constituent of aqueous extract of Pandanus odorus root. *J Ethnopharmacol, 62*(1), 79-84. <https://doi.org/10.1016/s0378-8741(98)00061-0>

Pham, A. T., Malterud, K. E., Paulsen, B. S., Diallo, D., & Wangensteen, H. J. P. B. (2014). α-Glucosidase inhibition, 15-lipoxygenase inhibition, and brine shrimp toxicity of extracts and isolated compounds from Terminalia macroptera leaves. *Pharmaceutical Biology, 52*(9), 1166-1169.

Phukhatmuen, P., Raksat, A., Laphookhieo, S., Charoensup, R., Duangyod, T., & Maneerat, W. (2020, Apr). Bioassay-guided isolation and identification of antidiabetic compounds from Garcinia cowa leaf extract. *Heliyon, 6*(4), e03625. <https://doi.org/10.1016/j.heliyon.2020.e03625>

Phuwapraisirisan, P., Puksasook, T., Jong-Aramruang, J., & Kokpol, U. (2008, Sep 15). Phenylethyl cinnamides: a new series of alpha-glucosidase inhibitors from the leaves of Aegle marmelos. *Bioorg Med Chem Lett, 18*(18), 4956-4958. <https://doi.org/10.1016/j.bmcl.2008.08.024>

Ponnulakshmi, R., Shyamaladevi, B., Vijayalakshmi, P., Selvaraj, J. J. T. m., & methods. (2019). In silico and in vivo analysis to identify the antidiabetic activity of beta sitosterol in adipose tissue of high fat diet and sucrose induced type-2 diabetic experimental rats. *29*(4), 276-290.

Ponnusamy, S., Haldar, S., Mulani, F., Zinjarde, S., Thulasiram, H., & RaviKumar, A. (2015). Gedunin and Azadiradione: Human Pancreatic Alpha-Amylase Inhibiting Limonoids from Neem (Azadirachta indica) as Anti-Diabetic Agents. *PloS one, 10*(10), e0140113. <https://doi.org/10.1371/journal.pone.0140113>

Pritsas, A., Tomou, E. M., Tsitsigianni, E., Papaemmanouil, C. D., Diamantis, D. A., Chatzopoulou, P., Tzakos, A. G., & Skaltsa, H. (2020, Jul 31). Valorisation of stachysetin from cultivated Stachys iva Griseb. as anti-diabetic agent: a multi-spectroscopic and molecular docking approach. *J Biomol Struct Dyn*, 1-15. <https://doi.org/10.1080/07391102.2020.1799864>

Puri, D., Prabhu, K. M., & Murthy, P. S. (2002, Oct). Mechanism of action of a hypoglycemic principle isolated from fenugreek seeds. *Indian J Physiol Pharmacol, 46*(4), 457-462.

Qa'dan, F., Verspohl, E. J., Nahrstedt, A., Petereit, F., & Matalka, K. Z. (2009, Jul 15). Cinchonain Ib isolated from Eriobotrya japonica induces insulin secretion in vitro and in vivo. *J Ethnopharmacol, 124*(2), 224-227. <https://doi.org/10.1016/j.jep.2009.04.023>

Quang, T. H., Ngan, N. T., Minh, C. V., Kiem, P. V., Thao, N. P., Tai, B. H., Nhiem, N. X., Song, S. B., & Kim, Y. H. (2011, Nov 29). Effect of triterpenes and triterpene saponins from the stem bark of Kalopanax pictus on the transactivational activities of three PPAR subtypes. *Carbohydr Res, 346*(16), 2567-2575. <https://doi.org/10.1016/j.carres.2011.08.029>

Ramadhan, R., & Phuwapraisirisan, P. (2015, Feb). Arylalkanones from Horsfieldia macrobotrys are effective antidiabetic agents achieved by α-glucosidase inhibition and radical scavenging. *Nat Prod Commun, 10*(2), 325-328.

Rani, R., Singh, A., & Singh, A. (2021, 09/15). MORONIC ACID: A REVIEW. *International Journal of Current Pharmaceutical Research*, 28-31. <https://doi.org/10.22159/ijcpr.2021v13i5.1903>

Rao, Y. K., Lee, M. J., Chen, K., Lee, Y. C., Wu, W. S., & Tzeng, Y. M. (2011). Insulin-Mimetic Action of Rhoifolin and Cosmosiin Isolated from Citrus grandis (L.) Osbeck Leaves: Enhanced Adiponectin Secretion and Insulin Receptor Phosphorylation in 3T3-L1 Cells. *Evid Based Complement Alternat Med, 2011*, 624375. <https://doi.org/10.1093/ecam/nep204>

Reddy, S. V., Tiwari, A. K., Kumar, U. S., Rao, R. J., & Rao, J. M. (2005, Apr). Free radical scavenging, enzyme inhibitory constituents from antidiabetic Ayurvedic medicinal plant Hydnocarpus wightiana Blume. *Phytother Res, 19*(4), 277-281. <https://doi.org/10.1002/ptr.1491>

Rojo, L. E., Ribnicky, D., Logendra, S., Poulev, A., Rojas-Silva, P., Kuhn, P., Dorn, R., Grace, M. H., Lila, M. A., & Raskin, I. (2012, Mar 15). In Vitro and in Vivo Anti-Diabetic Effects of Anthocyanins from Maqui Berry (Aristotelia chilensis). *Food Chem, 131*(2), 387-396. <https://doi.org/10.1016/j.foodchem.2011.08.066>

Ruan, C. T., Lam, S. H., Lee, S. S., & Su, M. J. (2013, Jun 15). Hypoglycemic action of borapetoside A from the plant Tinospora crispa in mice. *Phytomedicine, 20*(8-9), 667-675. <https://doi.org/10.1016/j.phymed.2013.02.009>

Ryu, H. W., Cho, J. K., Curtis-Long, M. J., Yuk, H. J., Kim, Y. S., Jung, S., Kim, Y. S., Lee, B. W., & Park, K. H. (2011, Dec). α-Glucosidase inhibition and antihyperglycemic activity of prenylated xanthones from Garcinia mangostana. *Phytochemistry, 72*(17), 2148-2154. <https://doi.org/10.1016/j.phytochem.2011.08.007>

Saleem, S., Jafri, L., ul Haq, I., Chang, L. C., Calderwood, D., Green, B. D., & Mirza, B. (2014, Oct 28). Plants Fagonia cretica L. and Hedera nepalensis K. Koch contain natural compounds with potent dipeptidyl peptidase-4 (DPP-4) inhibitory activity. *J Ethnopharmacol, 156*, 26-32. <https://doi.org/10.1016/j.jep.2014.08.017>

Saltos, M. B. V., Puente, B. F. N., Faraone, I., Milella, L., De Tommasi, N., & Braca, A. J. P. L. (2015). Inhibitors of α-amylase and α-glucosidase from Andromachia igniaria Humb. & Bonpl. *Phytochemistry Letters, 14*, 45-50.

Sengupta, S., Mukherjee, A., Goswami, R., Basu, S. J. J. o. e. i., & chemistry, m. (2009). Hypoglycemic activity of the antioxidant saponarin, characterized as α-glucosidase inhibitor present in Tinospora cordifolia. *24*(3), 684-690.

Seo, K.-H., Ra, J.-E., Lee, S.-J., Lee, J. H., Kim, S. R., Lee, J. H., & Seo, W. D. (2015a, 2015/08/01). Anti-hyperglycemic activity of polyphenols isolated from barnyard millet (Echinochloa utilis L.) and their role inhibiting α-glucosidase. *Journal of the Korean Society for Applied Biological Chemistry, 58*(4), 571-579. <https://doi.org/10.1007/s13765-015-0070-6>

Seo, K.-H., Ra, J.-E., Lee, S.-J., Lee, J. H., Kim, S. R., Lee, J. H., & Seo, W. D. J. J. o. t. K. S. f. A. B. C. (2015b). Anti-hyperglycemic activity of polyphenols isolated from barnyard millet (Echinochloa utilis L.) and their role inhibiting α-glucosidase. *Applied Biological Chemistry, 58*(4), 571-579.

Seo, K.-H., Ra, J.-E., Lee, S.-J., Lee, J. H., Kim, S. R., Lee, J. H., & Seo, W. D. J. J. o. t. K. S. f. A. B. C. (2015c). Anti-hyperglycemic activity of polyphenols isolated from barnyard millet (Echinochloa utilis L.) and their role inhibiting α-glucosidase. *Journal of the Korean Society for Applied Biological Chemistry, 58*(4), 571-579.

Seong, S. H., Roy, A., Jung, H. A., Jung, H. J., & Choi, J. S. J. J. o. e. (2016). Protein tyrosine phosphatase 1B and α-glucosidase inhibitory activities of Pueraria lobata root and its constituents. *194*, 706-716.

Serra-Barcellona, C., Coll Aráoz, M. V., Cabrera, W. M., Habib, N. C., Honoré, S. M., Catalán, C. A., Grau, A., Genta, S. B., & Sánchez, S. S. (2014, Feb 25). Smallanthus macroscyphus: a new source of antidiabetic compounds. *Chem Biol Interact, 209*, 35-47. <https://doi.org/10.1016/j.cbi.2013.11.015>

Sezik, E., Aslan, M., Yesilada, E., & Ito, S. (2005, Jan 28). Hypoglycaemic activity of Gentiana olivieri and isolation of the active constituent through bioassay-directed fractionation techniques. *Life Sci, 76*(11), 1223-1238. <https://doi.org/10.1016/j.lfs.2004.07.024>

Shalaby, N. M., Abd-Alla, H. I., Aly, H. F., Albalawy, M. A., Shaker, K. H., & Bouajila, J. (2014). Preliminary in vitro and in vivo evaluation of antidiabetic activity of Ducrosia anethifolia Boiss. and its linear furanocoumarins. *Biomed Res Int, 2014*, 480545. <https://doi.org/10.1155/2014/480545>

Sharma, B., Salunke, R., Srivastava, S., Majumder, C., & Roy, P. (2009, Oct). Effects of guggulsterone isolated from Commiphora mukul in high fat diet induced diabetic rats. *Food Chem Toxicol, 47*(10), 2631-2639. <https://doi.org/10.1016/j.fct.2009.07.021>

Sharma, B., Salunke, R., Srivastava, S., Majumder, C., Roy, P. J. F., & Toxicology, C. (2009). Effects of guggulsterone isolated from Commiphora mukul in high fat diet induced diabetic rats. *47*(10), 2631-2639.

Sharma, K. R., Adhikari, A., Hafizur, R. M., Hameed, A., Raza, S. A., Kalauni, S. K., Miyazaki, J., & Choudhary, M. I. (2015, Oct). Potent Insulin Secretagogue from Scoparia dulcis Linn of Nepalese Origin. *Phytother Res, 29*(10), 1672-1675. <https://doi.org/10.1002/ptr.5412>

Shi, D., Guo, S., Jiang, B., Guo, C., Wang, T., Zhang, L., & Li, J. (2013, Jan 30). HPN, a synthetic analogue of bromophenol from red alga Rhodomela confervoides: synthesis and anti-diabetic effects in C57BL/KsJ-db/db mice. *Mar Drugs, 11*(2), 350-362. <https://doi.org/10.3390/md11020350>

Shi, Y., Meng, F., Liu, J., Wang, B. J. J. o. T., & Chemistry, C. (2019). In silico modeling and in vitro activity of vitexin and isovitexin against SGLT2. *18*(07), 1950035.

Shieh, J. P., Cheng, K. C., Chung, H. H., Kerh, Y. F., Yeh, C. H., & Cheng, J. T. (2011, Apr 27). Plasma glucose lowering mechanisms of catalpol, an active principle from roots of Rehmannia glutinosa, in streptozotocin-induced diabetic rats. *J Agric Food Chem, 59*(8), 3747-3753. <https://doi.org/10.1021/jf200069t>

Shirosaki, M., Goto, Y., Hirooka, S., Masuda, H., Koyama, T., & Yazawa, K. (2012). Peach leaf contains multiflorin a as a potent inhibitor of glucose absorption in the small intestine in mice. *Biol Pharm Bull, 35*(8), 1264-1268. <https://doi.org/10.1248/bpb.b12-00058>

Singh, J., Cumming, E., Manoharan, G., Kalasz, H., & Adeghate, E. (2011). Medicinal chemistry of the anti-diabetic effects of momordica charantia: active constituents and modes of actions. *Open Med Chem J, 5*(Suppl 2), 70-77. <https://doi.org/10.2174/1874104501105010070>

Singh, P., Jayaramaiah, R. H., Agawane, S. B., Vannuruswamy, G., Korwar, A. M., Anand, A., Dhaygude, V. S., Shaikh, M. L., Joshi, R. S., & Boppana, R. J. S. r. (2016). Potential dual role of eugenol in inhibiting advanced glycation end products in diabetes: proteomic and mechanistic insights. *6*(1), 1-13.

Srisurichan, S., & Pornpakakul, S. J. P. L. (2015). Triterpenoids from the seedpods of Holarrhena curtisii King and Gamble. *Phytochemistry Letters, 12*, 282-286.

Sriyatep, T., Siridechakorn, I., Maneerat, W., Pansanit, A., Ritthiwigrom, T., Andersen, R. J., & Laphookhieo, S. (2015, Feb 27). Bioactive prenylated xanthones from the young fruits and flowers of Garcinia cowa. *J Nat Prod, 78*(2), 265-271. <https://doi.org/10.1021/np5008476>

Subash-Babu, P., Ignacimuthu, S., Agastian, P., & Varghese, B. (2009, Apr 1). Partial regeneration of beta-cells in the islets of Langerhans by Nymphayol a sterol isolated from Nymphaea stellata (Willd.) flowers. *Bioorg Med Chem, 17*(7), 2864-2870. <https://doi.org/10.1016/j.bmc.2009.02.021>

Sunil, C., Duraipandiyan, V., Agastian, P., & Ignacimuthu, S. (2012, Dec). Antidiabetic effect of plumbagin isolated from Plumbago zeylanica L. root and its effect on GLUT4 translocation in streptozotocin-induced diabetic rats. *Food Chem Toxicol, 50*(12), 4356-4363. <https://doi.org/10.1016/j.fct.2012.08.046>

Susanti, D., Amiroudine, M. Z. A. M., Rezali, M. F., & Taher, M. J. N. p. r. (2013). Friedelin and lanosterol from Garcinia prainiana stimulated glucose uptake and adipocytes differentiation in 3T3-L1 adipocytes. *27*(4-5), 417-424.

Taleb-Senouci, D., Ghomari, H., Krouf, D., Bouderbala, S., Prost, J., Lacaille-Dubois, M. A., & Bouchenak, M. (2009, Jun). Antioxidant effect of Ajuga iva aqueous extract in streptozotocin-induced diabetic rats. *Phytomedicine, 16*(6-7), 623-631. <https://doi.org/10.1016/j.phymed.2008.12.004>

Tamrakar, A. K., Yadav, P. P., Tiwari, P., Maurya, R., & Srivastava, A. K. (2008, Aug 13). Identification of pongamol and karanjin as lead compounds with antihyperglycemic activity from Pongamia pinnata fruits. *J Ethnopharmacol, 118*(3), 435-439. <https://doi.org/10.1016/j.jep.2008.05.008>

Tan, M. J., Ye, J. M., Turner, N., Hohnen-Behrens, C., Ke, C. Q., Tang, C. P., Chen, T., Weiss, H. C., Gesing, E. R., Rowland, A., James, D. E., & Ye, Y. (2008, Mar). Antidiabetic activities of triterpenoids isolated from bitter melon associated with activation of the AMPK pathway. *Chem Biol, 15*(3), 263-273. <https://doi.org/10.1016/j.chembiol.2008.01.013>

Tanaka, M., Misawa, E., Ito, Y., Habara, N., Nomaguchi, K., Yamada, M., Toida, T., Hayasawa, H., Takase, M., Inagaki, M., & Higuchi, R. (2006, Jul). Identification of five phytosterols from Aloe vera gel as anti-diabetic compounds. *Biol Pharm Bull, 29*(7), 1418-1422. <https://doi.org/10.1248/bpb.29.1418>

Tasnuva, S., Qamar, U., Ghafoor, K., Sahena, F., Jahurul, M., Rukshana, A., Juliana, M., Al-Juhaimi, F. Y., Jalifah, L., & Jalal, K. J. N. p. r. (2019). α-glucosidase inhibitors isolated from Mimosa pudica L. *33*(10), 1495-1499.

Thao, N. P., Luyen, B. T., Jo, S. H., Hung, T. M., Cuong, N. X., Nam, N. H., Kwon, Y. I., Minh, C. V., & Kim, Y. H. (2014, Oct). Triterpenoid saponins from the roots of Rosa rugosa Thunb. as rat intestinal sucrase inhibitors. *Arch Pharm Res, 37*(10), 1280-1285. <https://doi.org/10.1007/s12272-014-0384-7>

Thao, N. P., Luyen, B. T., Tai, B. H., Yang, S. Y., Jo, S. H., Cuong, N. X., Nam, N. H., Kwon, Y. I., Minh, C. V., & Kim, Y. H. (2014, Feb 15). Rat intestinal sucrase inhibition of constituents from the roots of Rosa rugosa Thunb. *Bioorg Med Chem Lett, 24*(4), 1192-1196. <https://doi.org/10.1016/j.bmcl.2013.12.098>

Tian, L. Y., Bai, X., Chen, X. H., Fang, J. B., Liu, S. H., & Chen, J. C. (2010, Jun). Anti-diabetic effect of methylswertianin and bellidifolin from Swertia punicea Hemsl. and its potential mechanism. *Phytomedicine, 17*(7), 533-539. <https://doi.org/10.1016/j.phymed.2009.10.007>

Tiong, S. H., Looi, C. Y., Hazni, H., Arya, A., Paydar, M., Wong, W. F., Cheah, S. C., Mustafa, M. R., & Awang, K. (2013, Aug 15). Antidiabetic and antioxidant properties of alkaloids from Catharanthus roseus (L.) G. Don. *Molecules, 18*(8), 9770-9784. <https://doi.org/10.3390/molecules18089770>

Tran, H. H., Nguyen, M. C., Le, H. T., Nguyen, T. L., Pham, T. B., Chau, V. M., Nguyen, H. N., & Nguyen, T. D. (2014, Jan). Inhibitors of α-glucosidase and α-amylase from Cyperus rotundus. *Pharm Biol, 52*(1), 74-77. <https://doi.org/10.3109/13880209.2013.814692>

Tundis, R., Loizzo, M. R., Statti, G. A., & Menichini, F. (2007, Jun). Inhibitory effects on the digestive enzyme alpha-amylase of three Salsola species (Chenopodiaceae) in vitro. *Pharmazie, 62*(6), 473-475.

Umezawa, K., Kojima, I., Simizu, S., Lin, Y., Fukatsu, H., Koide, N., Nakade, Y., & Yoneda, M. (2018). Therapeutic activity of plant-derived alkaloid conophylline on metabolic syndrome and neurodegenerative disease models. *Human cell, 31*(2), 95-101.

Vaidya, H., Goyal, R. K., & Cheema, S. K. J. P. R. (2013). Anti‐diabetic activity of swertiamarin is due to an active metabolite, gentianine, that upregulates PPAR‐γ gene expression in 3T3‐L1 cells. *27*(4), 624-627.

Vieira, A. J., Beserra, F. P., Souza, M., Totti, B., & Rozza, A. J. C.-b. i. (2018). Limonene: Aroma of innovation in health and disease. *283*, 97-106.

Vinayagam, R., Xu, B. J. N., & metabolism. (2015). Antidiabetic properties of dietary flavonoids: a cellular mechanism review. *Nutrition & metabolism, 12*(1), 1-20.

Vo, Q. H., Nguyen, P. H., Zhao, B. T., Ali, M. Y., Choi, J. S., Min, B. S., Nguyen, T. H., & Woo, M. H. J. F. (2015). Protein tyrosine phosphatase 1B (PTP1B) inhibitory constituents from the aerial parts of Tradescantia spathacea Sw. *Fitoterapia, 103*, 113-121.

Waltner-Law, M. E., Wang, X. L., Law, B. K., Hall, R. K., Nawano, M., & Granner, D. K. (2002, Sep 20). Epigallocatechin gallate, a constituent of green tea, represses hepatic glucose production. *J Biol Chem, 277*(38), 34933-34940. <https://doi.org/10.1074/jbc.M204672200>

Wan, C., Yuan, T., Li, L., Kandhi, V., Cech, N. B., Xie, M., & Seeram, N. P. (2012, Jan 1). Maplexins, new α-glucosidase inhibitors from red maple (Acer rubrum) stems. *Bioorg Med Chem Lett, 22*(1), 597-600. <https://doi.org/10.1016/j.bmcl.2011.10.073>

Wang, H., Du, Y.-J., & Song, H.-C. J. F. c. (2010). α-Glucosidase and α-amylase inhibitory activities of guava leaves. *Food chemistry, 123*(1), 6-13.

Wang, J.-J., Zhao, R., Liang, J.-C., & Chen, Y. J. Y. x. x. b. A. p. S. (2014). The antidiabetic and hepatoprotective effects of magnolol on diabetic rats induced by high-fat diet and streptozotocin. *49*(4), 476-481.

Wang, Y., Xiang, L., Wang, C., Tang, C., & He, X. (2013). Antidiabetic and antioxidant effects and phytochemicals of mulberry fruit (Morus alba L.) polyphenol enhanced extract. *PLoS One, 8*(7), e71144. <https://doi.org/10.1371/journal.pone.0071144>

Watanabe, A., Kato, T., Ito, Y., Yoshida, I., Harada, T., Mishima, T., Fujita, K., Watai, M., Nakagawa, K., & Miyazawa, T. (2014, Oct 31). Aculeatin, a coumarin derived from Toddalia asiatica (L.) Lam., enhances differentiation and lipolysis of 3T3-L1 adipocytes. *Biochem Biophys Res Commun, 453*(4), 787-792. <https://doi.org/10.1016/j.bbrc.2014.10.027>

Weidner, C., de Groot, J. C., Prasad, A., Freiwald, A., Quedenau, C., Kliem, M., Witzke, A., Kodelja, V., Han, C.-T., & Giegold, S. (2012). Amorfrutins are potent antidiabetic dietary natural products. *Proceedings of the National Academy of Sciences of the United States of America, 109*(19), 7257-7262.

Xu, D., Wang, Q., Zhang, W., Hu, B., Zhou, L., Zeng, X., & Sun, Y. (2015, Apr 15). Inhibitory activities of caffeoylquinic acid derivatives from Ilex kudingcha C.J. Tseng on α-glucosidase from Saccharomyces cerevisiae. *J Agric Food Chem, 63*(14), 3694-3703. <https://doi.org/10.1021/acs.jafc.5b00420>

Xu, F., Wu, H., Wang, X., Yang, Y., Wang, Y., Qian, H., & Zhang, Y. (2014, Sep 9). RP-HPLC characterization of lupenone and β-sitosterol in rhizoma musae and evaluation of the anti-diabetic activity of lupenone in diabetic Sprague-Dawley rats. *Molecules, 19*(9), 14114-14127. <https://doi.org/10.3390/molecules190914114>

Yamamoto, N., Kawabata, K., Sawada, K., Ueda, M., Fukuda, I., Kawasaki, K., Murakami, A., & Ashida, H. (2011, Aug). Cardamonin stimulates glucose uptake through translocation of glucose transporter-4 in L6 myotubes. *Phytother Res, 25*(8), 1218-1224. <https://doi.org/10.1002/ptr.3416>

Yang, S., Na, M. K., Jang, J. P., Kim, K. A., Kim, B. Y., Sung, N. J., Oh, W. K., & Ahn, J. S. (2006, Aug). Inhibition of protein tyrosine phosphatase 1B by lignans from Myristica fragrans. *Phytother Res, 20*(8), 680-682. <https://doi.org/10.1002/ptr.1935>

Yang, T. C., Chao, H. F., Shi, L. S., Chang, T. C., Lin, H. C., & Chang, W. L. (2014, Mar). Alkaloids from Coptis chinensis root promote glucose uptake in C2C12 myotubes. *Fitoterapia, 93*, 239-244. <https://doi.org/10.1016/j.fitote.2014.01.008>

Yang, Y., Gu, L., Xiao, Y., Liu, Q., Hu, H., Wang, Z., & Chen, K. (2015). Rapid identification of α-glucosidase inhibitors from Phlomis tuberosa by Sepbox chromatography and thin-layer chromatography bioautography. *PloS one, 10*(2), e0116922. <https://doi.org/10.1371/journal.pone.0116922>

Yokozawa, T., Kim, H. Y., Cho, E. J., Choi, J. S., Chung, H. Y. J. J. o. a., & chemistry, f. (2002). Antioxidant effects of isorhamnetin 3, 7-di-O-β-D-glucopyranoside isolated from mustard leaf (Brassica juncea) in rats with streptozotocin-induced diabetes. *50*(19), 5490-5495.

Yoshida, J., Seino, H., Ito, Y., Nakano, T., Satoh, T., Ogane, Y., Suwa, S., Koshino, H., Kimura, K.-i. J. J. o. a., & chemistry, f. (2013). Inhibition of glycogen synthase kinase-3β by falcarindiol isolated from Japanese Parsley (Oenanthe javanica). *61*(31), 7515-7521.

Yoshinari, O., Sato, H., & Igarashi, K. (2009, May). Anti-diabetic effects of pumpkin and its components, trigonelline and nicotinic acid, on Goto-Kakizaki rats. *Biosci Biotechnol Biochem, 73*(5), 1033-1041. <https://doi.org/10.1271/bbb.80805>

Yu, B.-C., Chen, W.-C., & Cheng, J.-T. J. P. m. (2003). Antihyperglycemic effect of andrographolide in streptozotocin-induced diabetic rats. *Planta medica, 69*(12), 1075-1079.

Yu, H.-t., Zhen, J., Pang, B., Gu, J.-n., & Wu, S.-s. (2015). Ginsenoside Rg1 ameliorates oxidative stress and myocardial apoptosis in streptozotocin-induced diabetic rats. *Journal of Zhejiang University-Science B, 16*(5), 344-354.

Yuan, T., Ding, Y., Wan, C., Li, L., Xu, J., Liu, K., Slitt, A., Ferreira, D., Khan, I. A., & Seeram, N. P. (2012, Oct 19). Antidiabetic ellagitannins from pomegranate flowers: inhibition of α-glucosidase and lipogenic gene expression. *Org Lett, 14*(20), 5358-5361. <https://doi.org/10.1021/ol302548c>

Zanatta, L., Rosso, A., Folador, P., Figueiredo, M. S., Pizzolatti, M. G., Leite, L. D., & Silva, F. R. (2008, Apr). Insulinomimetic effect of kaempferol 3-neohesperidoside on the rat soleus muscle. *J Nat Prod, 71*(4), 532-535. <https://doi.org/10.1021/np070358>+

Zhang, H., Matsuda, H., Kumahara, A., Ito, Y., Nakamura, S., & Yoshikawa, M. (2007, Sep 1). New type of anti-diabetic compounds from the processed leaves of Hydrangea macrophylla var. thunbergii (Hydrangeae Dulcis Folium). *Bioorg Med Chem Lett, 17*(17), 4972-4976. <https://doi.org/10.1016/j.bmcl.2007.06.027>

Zhang, M., Chen, M., Zhang, H. Q., Sun, S., Xia, B., & Wu, F. H. (2009, Dec). In vivo hypoglycemic effects of phenolics from the root bark of Morus alba. *Fitoterapia, 80*(8), 475-477. <https://doi.org/10.1016/j.fitote.2009.06.009>

Zhang, X. S., Bi, X. L., Wan, X., Cao, J. Q., Xia, X. C., Diao, Y. P., & Zhao, Y. Q. (2013, Jan 1). Protein tyrosine phosphatase 1B inhibitory effect by dammarane-type triterpenes from hydrolyzate of total Gynostemma pentaphyllum saponins. *Bioorg Med Chem Lett, 23*(1), 297-300. <https://doi.org/10.1016/j.bmcl.2012.10.097>

Zhang, Y., Cai, J., Ruan, H., Pi, H., & Wu, J. (2007, Nov 1). Antihyperglycemic activity of kinsenoside, a high yielding constituent from Anoectochilus roxburghii in streptozotocin diabetic rats. *J Ethnopharmacol, 114*(2), 141-145. <https://doi.org/10.1016/j.jep.2007.05.022>

Zhang, Y., Li, Y., Guo, Y. W., Jiang, H. L., & Shen, X. (2009, Mar). A sesquiterpene quinone, dysidine, from the sponge Dysidea villosa, activates the insulin pathway through inhibition of PTPases. *Acta Pharmacol Sin, 30*(3), 333-345. <https://doi.org/10.1038/aps.2009.5>

Zhang, Y. L., Luo, J. G., Wan, C. X., Zhou, Z. B., & Kong, L. Y. (2014, Jan). Geranylated 2-arylbenzofurans from Morus alba var. tatarica and their α-glucosidase and protein tyrosine phosphatase 1B inhibitory activities. *Fitoterapia, 92*, 116-126. <https://doi.org/10.1016/j.fitote.2013.10.017>

Zhang, Y. L., Luo, J. G., Wan, C. X., Zhou, Z. B., & Kong, L. Y. (2015, Nov). Four New Flavonoids with α-Glucosidase Inhibitory Activities from Morus alba var. tatarica. *Chem Biodivers, 12*(11), 1768-1776. <https://doi.org/10.1002/cbdv.201500005>

Zhang, Y. L., Luo, J. G., Wan, C. X., Zhou, Z. B., Kong, L. Y. J. C., & biodiversity. (2015). Four New Flavonoids with α‐Glucosidase Inhibitory Activities from Morus alba var. tatarica. *Chemistry & biodiversity, 12*(11), 1768-1776.

Zhao, D. G., Zhou, A. Y., Du, Z., Zhang, Y., Zhang, K., & Ma, Y. Y. (2015, Dec). Coumarins with α-glucosidase and α-amylase inhibitory activities from the flower of Edgeworthia gardneri. *Fitoterapia, 107*, 122-127. <https://doi.org/10.1016/j.fitote.2015.10.012>

Zhou, H., Xing, J., Liu, S., Song, F., Cai, Z., Pi, Z., Liu, Z., & Liu, S. (2012, Jul-Aug). Screening and determination for potential α-glucosidase inhibitors from leaves of Acanthopanax senticosus harms by using UF-LC/MS and ESI-MS(n). *Phytochem Anal, 23*(4), 315-323. <https://doi.org/10.1002/pca.1360>

Zhou, P., Xie, W., He, S., Sun, Y., Meng, X., Sun, G., & Sun, X. (2019). Ginsenoside Rb1 as an anti-diabetic agent and its underlying mechanism analysis. *Cells, 8*(3), 204.

Zhu, Y., Zhang, Y., Liu, Y., Chu, H., & Duan, H. J. M. (2010). Synthesis and biological activity of trans-tiliroside derivatives as potent anti-diabetic agents. *15*(12), 9174-9183.
